# Supplementary material for: Associations of presenting symptoms and subsequent adverse clinical outcomes in people with unipolar depression: a prospective natural language processing (NLP), transdiagnostic, network analysis of electronic health record (EHR) data
Source: BMJ Open. 2022 Apr 29;12(4):e056541. doi: 10.1136/bmjopen-2021-056541 (PMC9058769; doi:10.1136/bmjopen-2021-056541)
Supplement: Supplementary data [file bmjopen-2021-056541supp001.pdf]

BMJ Publishing Group Limited (BMJ) disclaims all liability and responsibility arising from any reliance placed on this supplemental material which has been supplied by the author(s)

SUPPLEMENTARY MATERIAL

Associations of presenting symptoms and subsequent bipolar/psychotic disorder onset in people with unipolar depression: a prospective natural language processing (NLP), transdiagnostic, network analysis of electronic health record (EHR) data

Table of Contents

Table of Contents 1

eMethods 1: Network analysis 2

eTable 1: Diagnosis inclusion criteria 2

eTable 2: Ethnicity classification 3

eTable 3: Categorisation of symptoms 4

eTable 4: "TRANSd"iagnostic research recommendations 5

eTable 5: Centrality definitions[2] 6

eTable 6: Frequency of recorded ICD-10 unipolar depression diagnoses 7

eTable 7: Multivaribale Cox regression models examining factors associated with subsequent bipolar disorder diagnosis 8

eTable 8: Multivariable Cox regression models examining factors associated with subsequent psychotic disorder diagnosis 9

eTable 9: Multivariable Cox proportional hazards regression examining factors associated with compulsory psychiatric hospital admission 10

eTable 10: DSM-5 mixed features specifier of depression[3] 11

eFigure 1: Cohort definition and missing data 12

eFigure 2: Centrality indices for overall network 13

References 14

Patel R, *et al. BMJ Open* 2022; 12:e056541. doi: 10.1136/bmjopen-2021-056541

eMethods 1: Network analysis

The Enhanced Least Absolute Shrinkage and Selection Operator (eLASSO) procedure implemented via the *EstimateNetwork* package is based on the Ising model which is indicated for analysis of binary data. The method combines L1-regularised logistic regression with model selection using a tuning parameter selected by minimizing the Extended Bayesian Information Criterion (EBIC) to achieve sparsity. Sparse symptom networks have much fewer links between symptoms than the possible maximum number of links within that network (where every symptom would be connected). The use of LASSO regularization creates a more parsimonious network model that returns only the most prominent associations between symptoms, and excludes spurious associations. This in turn maximises model interpretability.[1]

In terms of centrality indices, we calculated *strength, closeness* and *betweenness* defined in eTable 4. Centrality indices measure how likely a symptom is to mediate associations between other symptoms or be strongly connected to other symptoms, regardless of that symptom's prevalence. In brief, nodes of high *strength* are likely to be associated with most members of the network, low *closeness* centrality indicates that a node is closely connected with most other nodes in the network (less likely to be peripheral) and *betweenness* centrality indicates the amount of influence a node has over the flow of information in a graph.[2]

We used a case-dropping bootstrapping procedure to assess network stability. In this procedure, the network is reconstructed, and symptom centrality indices are recalculated over multiple iterations in which cases are dropped in increasing proportions. This generates a Correlation Stability Coefficient, which indicates the maximum proportion of cases that can be dropped from the sample without causing the correlation between the original and bootstrapped centrality values to drop below r = 0.7. The network is deemed to be stable if the correlation stability coefficient survives above 0.5.[1]

eTable 1: Diagnosis inclusion criteria

ICD-10 code	Diagnosis
F32 – Depressive episode	F32.9 - Depressive episode, unspecified<br>F32.2 - Severe depressive episode without psychotic symptoms<br>F32.0 - Mild depressive episode<br>F32.1 - Moderate depressive episode<br>F32.8 - Other depressive episodes
F33 – Recurrent depressive disorder	F33.1 - Recurrent depressive disorder, current episode moderate<br>F33.4 - Recurrent depressive disorder, currently in remission<br>F33.0 - Recurrent depressive disorder, current episode mild<br>F33.8 - Other recurrent depressive disorders<br>F33.9 - Recurrent depressive disorder, unspecified
F41 – Anxiety disorder	F41.2 - Mixed anxiety and depressive disorder

Note: All diagnoses were drawn from the structured field Primary Diagnosis *in EHR*

eTable 2: Ethnicity classification

**Ethnic group**	**Ethnicity as recorded in patient electronic health records**
Black	Black or Black British - African
	Black or Black British - Caribbean
	Black or Black British - Any other Black background
White	White - British
	White - Irish
	White - Any other White background
Asian	Asian or Asian British - Bangladeshi
	Asian or Asian British - Indian
	Asian or Asian British - Pakistani
	Asian or Asian British - Any other Asian background
	Other Ethnic Groups – Chinese
Mixed	Mixed - White and Asian
	Mixed - White and Black African
	Mixed - White and Black Caribbean
	Mixed - Any other mixed background
Other	Other Ethnic Groups - Any other ethnic group
Missing	Not Stated
	Not Recorded

eTable 3: Categorisation of symptoms

Depressive symptoms	Overlapping symptoms	Mania symptoms
• Disturbed mood	• Poor concentration	• Irritability
• Anhedonia	• Insomnia	• Elation
• Guilt	• Agitation	• Grandiosity
• Weight loss	• Disturbed sleep	• Flight of ideas
• Reduced appetite	• Mood instability	• Pressured speech
• Hopelessness		
• Helplessness		
• Worthlessness		
• Tearfulness		
• Low energy		

Patel R, *et al. BMJ Open* 2022; 12:e056541. doi: 10.1136/bmjopen-2021-056541

eTable 4: “TRANSd”iagnostic research recommendations

Transparent definition of the gold standard (ICD, DSM, other), including specific diagnostic types, official codes, primary vs. secondary diagnoses, diagnostic assessment interviews.

Report the primary outcome of the study, the study design and the definition of the transdiagnostic construct in the abstract and main text.

Appraise the conceptual framework/approach of the transdiagnostic approach: across-diagnoses, beyond-diagnoses, other (explain).

Numerate the diagnostic categories, spectra and non-clinical samples in which the transdiagnostic construct is being tested and then validated.

Show the degree of improvement of the transdiagnostic approach against the specific diagnostic approach through specific comparative analyses.

Demonstrate the generalizability of the transdiagnostic construct through external validation studies.

Fusar-Poli P, Solmi M, Brondino N, et al. Transdiagnostic psychiatry: a systematic review. World Psychiatry 2019;18:192–207. doi:https://doi.org/10.1002/wps.20631

eTable 5: Centrality definitions[2]

Centrality	Calculation	Interpretation
**Betweenness**	The percentage of shortest paths that must go through that specific node.	Describes the amount of influence a node has over the flow of information in a graph. Useful to find nodes that bridge from one part of a graph to another.
**Closeness**	The average distance between one node and all other nodes in the network (by taking the inverse of each edge weight).	Low closeness centrality indicates that node is closely connected with most other nodes, therefore it can spread information most efficiently through a network.
**Strength**	The sum of all absolute edge weights a node is directly connected to.	Likely to be involved with most members of the network.

eTable 6: Frequency of recorded ICD-10 unipolar depression diagnoses

ICD-10 Code	ICD-10 Diagnosis	n
F32.1	Moderate depressive episode	5,242
F32	Depressive episode	3,998
F41.2	Mixed anxiety and depressive disorder	2,137
F32.0	Mild depressive episode	2,043
F32.9	Depressive episode, unspecified	1,424
F33.1	Recurrent depressive disorder, current episode moderate	1,257
F32.2	Severe depressive episode without psychotic symptoms	1,231
F33	Recurrent depressive disorder	1,123
F33.0	Recurrent depressive disorder, current episode mild	408
F32.8	Other depressive episodes	348
F33.4	Recurrent depressive disorder, currently in remission	326
F33.9	Recurrent depressive disorder, unspecified	276
F33.8	Other recurrent depressive disorders	67

Patel R, *et al. BMJ Open* 2022; 12:e056541. doi: 10.1136/bmjopen-2021-056541

eTable 7: Multivaribale Cox regression models examining factors associated with subsequent bipolar disorder diagnosis

Predictor	Risk of developing bipolar disorder (number of events = 986)			
	Univariate Hazard Ratio (95% CI)	p value	[a]Multivariable Hazard Ratio (95% CI)	p value
**Demographics**				
Age (years)	0.99 (0.98 to 0.99)	<0.001***	0.99 (0.98 to 0.99)	<0.001***
Male gender	0.73 (0.64 to 0.84)	<0.001***	0.77 (0.67 to 0.88)	<0.001***
Ethnicity				
White	*Ref*		*Ref*	*Ref*
Asian	0.86 (0.64 to 1.16)	0.356	0.82 (0.61 to 1.10)	0.229
Black	1.04 (0.88 to 1.22)	0.671	0.96 (0.82 to 1.14)	0.654
Mixed	1.35 (0.93 to 1.95)	0.137	1.21 (0.84 to 1.76)	0.331
Other	0.94 (0.75 to 1.18)	0.621	0.91 (0.72 to 1.14)	0.414
**Symptom groups**				
Mania (≥ 1 symptom)	2.38 (2.10 to 2.71)	<0.001***	2.39 (2.11 to 2.72)	<0.001***
Overlapping (≥ 1 symptom)	1.43 (1.19 to 1.71)	<0.001***	1.45 (1.21 to 1.74)	<0.001***
Depression (≥ 1 symptom)	1.55 (1.09 to 2.19)	0.021*	1.45 (1.02 to 2.05)	0.050*
**Individual symptoms**				
Irritability	1.99 (1.74 to 2.28)	<0.001***	1.99 (1.74 to 2.28)	<0.001***
Elation	4.21 (3.46 to 5.11)	<0.001***	4.21 (3.46 to 5.12)	<0.001***
Pressured speech	3.87 (2.97 to 5.05)	<0.001***	4.07 (3.12 to 5.31)	<0.001***
Flight of ideas	3.78 (2.75 to 5.20)	<0.001***	3.90 (2.84 to 5.36)	<0.001***
Grandiosity	4.14 (3.08 to 5.56)	<0.001***	4.36 (3.24 to 5.87)	<0.001***
Disturbed sleep	1.13 (0.99 to 1.28)	0.089	1.15 (1.00 to 1.31)	0.054
Poor concentration	1.42 (1.25 to 1.61)	<0.001***	1.43 (1.26 to 1.62)	<0.001***
Mood instability	2.08 (1.83 to 2.36)	<0.001***	2.03 (1.79 to 2.30)	<0.001***
Agitation	2.19 (1.93 to 2.49)	<0.001***	2.31 (2.03 to 2.63)	<0.001***
Insomnia	1.52 (1.32 to 1.77)	<0.001***	1.54 (1.33 to 1.78)	<0.001***

[a]Multivariable analysis adjusted for age, gender and ethnicity.
* p < 0.05, ** p < 0.001, *** p < 0.001

Patel R, *et al. BMJ Open* 2022; 12:e056541. doi: 10.1136/bmjopen-2021-056541

eTable 8: Multivariable Cox regression models examining factors associated with subsequent psychotic disorder diagnosis

**Predictor**	**Risk of developing psychotic disorder (number of events = 2,150)**			
	**Univariate Hazard Ratio (95% CI)**	**p value**	**[a]Multivariable Hazard Ratio (95% CI)**	**p value**
**Demographics**				
Age (years)	1.01 (1.00 to 1.01)	<0.001***	1.01 (1.01 to 1.01)	<0.001***
Male gender	1.10 (1.01 to 1.20)	0.036*	1.12 (1.03 to 1.23)	0.010*
Ethnicity				
White	*Ref*		*Ref*	*Ref*
Asian	1.73 (1.47 to 2.03)	<0.001***	1.80 (1.53 to 2.11)	<0.001***
Black	1.83 (1.65 to 2.02)	<0.001***	1.92 (1.73 to 2.12)	<0.001***
Mixed	0.93 (0.67 to 1.29)	0.671	1.00 (0.72 to 1.39)	0.999
Other	1.03 (0.89 to 1.20)	0.671	1.06 (0.92 to 1.23)	0.448
**Symptom groups**				
Mania (≥ 1 symptom)	1.87 (1.71 to 2.05)	<0.001***	1.89 (1.73 to 2.07)	<0.001***
Overlapping (≥ 1 symptom)	1.85 (1.62 to 2.11)	<0.001***	1.81 (1.59 to 2.07)	<0.001***
Depression (≥ 1 symptom)	1.29 (1.02 to 1.63)	0.038*	1.32 (1.04 to 1.67)	0.023*
**Individual symptoms**				
Irritability	1.80 (1.64 to 1.98)	<0.001***	1.82 (1.65 to 1.99)	<0.001***
Elation	2.04 (1.71 to 2.44)	<0.001***	2.17 (1.81 to 2.60)	<0.001***
Pressured speech	2.67 (2.16 to 3.32)	<0.001***	2.69 (2.17 to 3.33)	<0.001***
Flight of ideas	2.63 (2.08 to 3.32)	<0.001***	2.61 (2.07 to 3.30)	<0.001***
Grandiosity	2.59 (2.02 to 3.33)	<0.001***	2.61 (2.03 to 3.36)	<0.001***
Disturbed sleep	1.71 (1.56 to 1.88)	<0.001***	1.65 (1.50 to 1.81)	<0.001***
Poor concentration	1.56 (1.44 to 1.70)	<0.001***	1.54 (1.42 to 1.68)	<0.001***
Mood instability	1.62 (1.48 to 1.76)	<0.001***	1.70 (1.55 to 1.85)	<0.001***
Agitation	2.42 (2.22 to 2.63)	<0.001***	2.41 (2.21 to 2.63)	<0.001***
Insomnia	1.49 (1.35 to 1.65)	<0.001***	1.50 (1.35 to 1.65)	<0.001***

[a]Multivariable analysis adjusted for age, gender and ethnicity.
* p < 0.05, ** p <0.01, *** p < 0.001

Patel R, *et al. BMJ Open* 2022; 12:e056541. doi: 10.1136/bmjopen-2021-056541

eTable 9: Multivariable Cox proportional hazards regression examining factors associated with compulsory psychiatric hospital admission

Predictor	Risk of compulsory psychiatric hospital admission under the UK Mental Health Act (number of events = 516)			
	Univariate Hazard Ratio (95% CI)	p value	[a]Multivariable Hazard Ratio (95% CI)	p value
**Demographics**				
Age (years)	1.00 (0.99 to 1.01)	0.91	1.00 (0.99 to 1.01)	0.794
Male gender	1.23 (1.03 to 1.46)	0.029**	1.34 (1.12 to 1.60)	0.002**
Ethnicity				
White	*Ref*	*Ref*	*Ref*	*Ref*
Asian	1.63 (1.16 to 2.29)	0.007**	1.65 (1.17 to 2.32)	0.006**
Black	2.36 (1.95 to 2.86)	<0.001***	2.47 (2.03 to 3.00)	<0.001***
Mixed	1.22 (0.67 to 2.23)	0.560	1.27 (0.70 to 2.33)	0.465
Other	0.72 (0.51 to 1.02)	0.078	0.73 (0.51 to 1.03)	0.084
**Symptom groups**				
Mania (≥ 1 symptom)	2.29 (1.92 to 2.73)	<0.001***	2.28 (1.92 to 2.72)	<0.001***
Overlapping (≥ 1 symptom)	1.68 (1.29 to 2.19)	<0.001***	1.62 (1.25 to 2.11)	0.001**
Depression (≥ 1 symptom)	0.99 (0.63 to 1.54)	0.950	1.00 (0.64 to 1.57)	0.999
**Individual symptoms**				
Irritability	2.29 (1.91 to 2.74)	<0.001***	2.28 (1.90 to 2.73)	<0.001***
Elation	3.64 (2.75 to 4.83)	<0.001***	3.96 (2.98 to 5.25)	<0.001***
Pressured speech	4.41 (3.14 to 6.19)	<0.001***	4.37 (3.12 to 6.14)	<0.001***
Flight of ideas	3.91 (2.69 to 5.69)	<0.001***	3.80 (2.61 to 5.53)	<0.001***
Grandiosity	3.83 (2.54 to 5.77)	<0.001***	3.59 (2.38 to 5.41)	<0.001***
Disturbed sleep	1.55 (1.28 to 1.87)	<0.001***	1.47 (1.21 to 1.78)	<0.001***
Poor concentration	1.48 (1.24 to 1.76)	<0.001***	1.45 (1.22 to 1.73)	<0.001***
Mood instability	1.84 (1.54 to 2.19)	<0.001***	1.92 (1.61 to 2.29)	<0.001***
Agitation	3.75 (3.15 to 4.46)	<0.001***	3.69 (3.10 to 4.39)	<0.001***
Insomnia	1.50 (1.2 to 1.84)	<0.001***	1.51 (1.23 to 1.86)	<0.001***

[a]Multivariable analysis adjusted for age, gender and ethnicity.
* p < 0.05, ** p < 0.01, *** p < 0.001

Patel R, *et al. BMJ Open* 2022; 12:e056541. doi: 10.1136/bmjopen-2021-056541

eTable 10: DSM-5 mixed features specifier of depression[3]

**Depression with mixed features specifier**
A. At least three of the following manic/hypomanic symptoms are present nearly every day during the majority of days of a major depressive episode:<br>1. Elevated, expansive mood<br>2. Inflated self-esteem or grandiosity<br>3. More talkative than usual or pressure to keep talking<br>4. Flight of ideas or subjective experience that thoughts are racing<br>5. Increase in energy or goal-directed activity (either socially, at work or school, or sexually)<br>6. Increased or excessive involvement in activities that have a high potential for painful consequences<br>7. Decreased need for sleep (feeling rested despite sleeping less than usual; to be contrasted with insomnia)<br><br>B. Mixed symptoms are observable by others and represent a change from the person's usual behaviour.<br>C. For individuals whose symptoms meet full criteria for either mania or hypomania, the diagnosis should be bipolar I or bipolar II disorder.<br>D. The mixed symptoms are not attributable to the physiological effects of a substance (e.g., a drug of abuse, a medication or other treatment).<br><br>**Note:** Mixed features associated with a major depressive episode have been found to be a significant risk factor for the development of bipolar I or bipolar II disorder. As a result, it is clinically useful to note the presence of this specifier for treatment planning and monitoring of response to treatment.

eFigure 1: Cohort definition and missing data

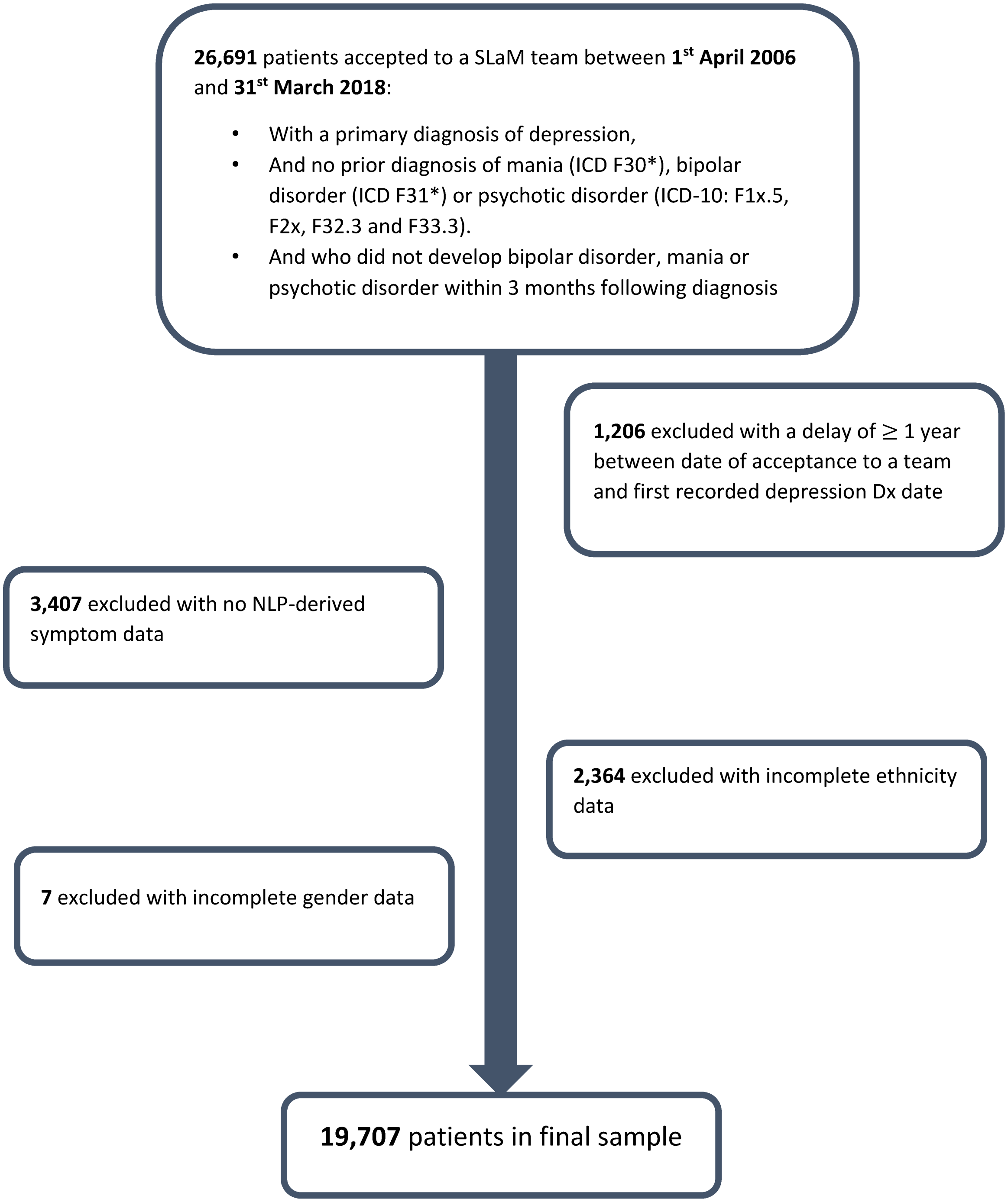

Patel R, *et al. BMJ Open* 2022; 12:e056541. doi: 10.1136/bmjopen-2021-056541

eFigure 2: Centrality indices for overall network

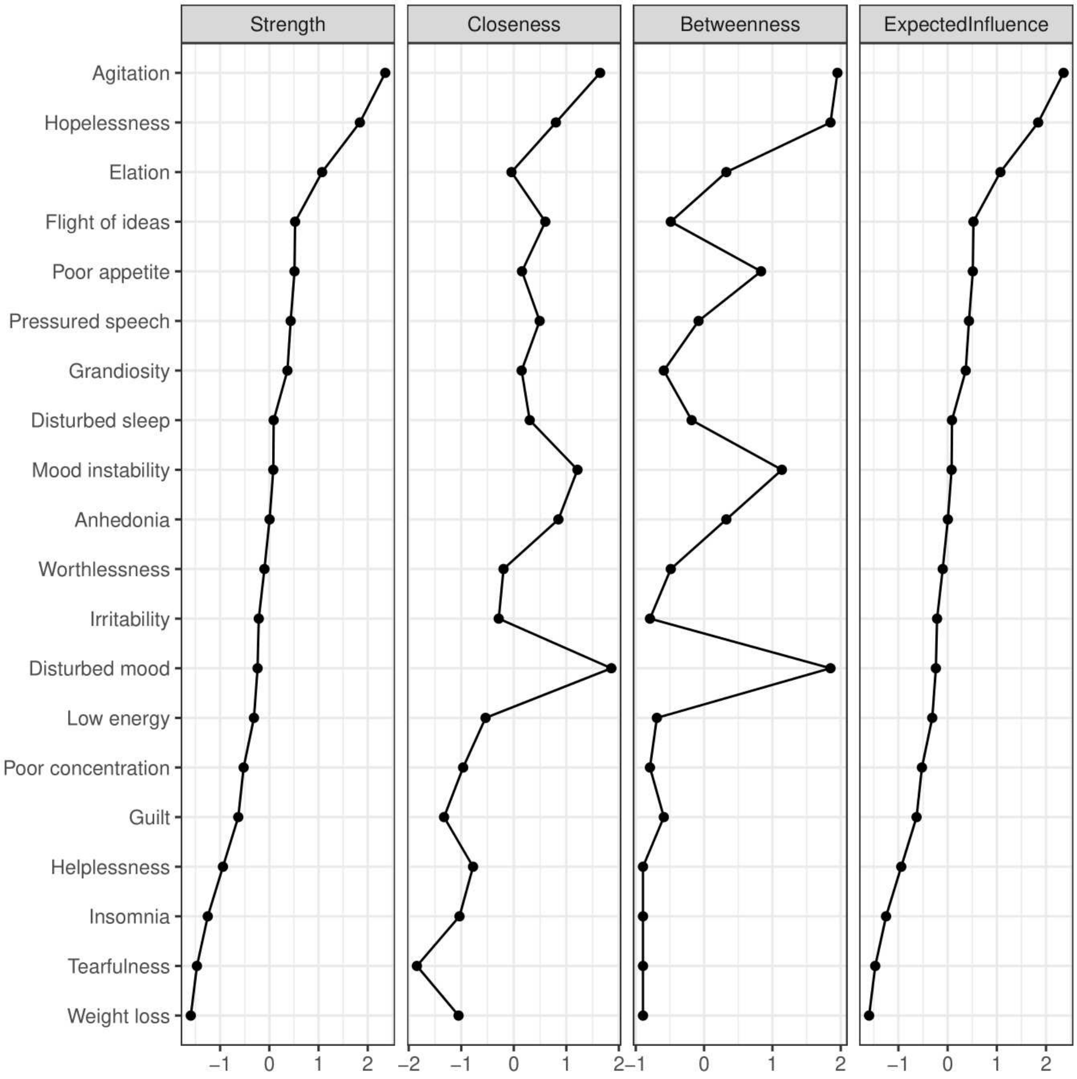

Note: Centrality indices are reported as standardised z scores to facilitate comparison.

References

1 Epskamp S, Borsboom D, Fried EI. Estimating psychological networks and their accuracy: A tutorial paper. *Behav Res Methods* 2018;**50**:195–212. doi:10.3758/s13428-017-0862-1

2 Golbeck J. *Analyzing the Social Web*. Elsevier Inc. 2013. doi:10.1016/C2012-0-00171-8

3 American Psychological Association. *Diagnostic and statistical manual of mental disorders : DSM-5*. Fifth edition. Arlington, VA : American Psychiatric Publishing, [2013] ©2013 2013. https://search.library.wisc.edu/catalog/9911111397702121
