# Supplementary material for: Associations of presenting symptoms and subsequent adverse clinical outcomes in people with unipolar depression: a prospective natural language processing (NLP), transdiagnostic, network analysis of electronic health record (EHR) data
Source: BMJ Open. 2022 Apr 29;12(4):e056541. doi: 10.1136/bmjopen-2021-056541 (PMC9058769; doi:10.1136/bmjopen-2021-056541)
Supplement: Author's manuscript [file bmjopen-2021-056541.draft_revisions.pdf]

BMJ Open is committed to open peer review. As part of this commitment we make the peer review history of every article we publish publicly available.

When an article is published we post the peer reviewers' comments and the authors' responses online. We also post the versions of the paper that were used during peer review. These are the versions that the peer review comments apply to.

The versions of the paper that follow are the versions that were submitted during the peer review process. They are not the versions of record or the final published versions. They should not be cited or distributed as the published version of this manuscript.

BMJ Open is an open access journal and the full, final, typeset and author-corrected version of record of the manuscript is available on our site with no access controls, subscription charges or pay-per-view fees (http://bmjopen.bmj.com).

If you have any questions on BMJ Open's open peer review process please email info.bmjopen@bmj.com

BMJ Open

Associations of presenting symptoms and subsequent bipolar/psychotic disorder onset in people with unipolar depression: a prospective natural language processing, transdiagnostic, network analysis of electronic health record data

Journal:	*BMJ Open*
Manuscript ID	bmjopen-2021-056541
Article Type:	Original research
Date Submitted by the Author:	17-Aug-2021

Complete List of Authors:	Patel, Rashmi; King's College London Institute of Psychiatry Psychology and Neuroscience, Department of Psychosis Studies; South London and Maudsley NHS Foundation Trust<br>Irving, Jessica; King's College London Institute of Psychiatry Psychology and Neuroscience, Department of Psychosis Studies<br>Brinn, Aimee; King's College London Institute of Psychiatry Psychology and Neuroscience, Department of Psychosis Studies<br>Taylor, Matthew; University of Oxford Department of Psychiatry<br>Shetty, Hitesh; South London and Maudsley NHS Foundation Trust<br>Pritchard, Megan; King's College London Institute of Psychiatry Psychology and Neuroscience, Department of Psychological Medicine; South London and Maudsley NHS Foundation Trust<br>Stewart, Robert; King's College London Institute of Psychiatry Psychology and Neuroscience, Department of Psychological Medicine; South London and Maudsley NHS Foundation Trust<br>Fusar-Poli, Paolo; King's College London Institute of Psychiatry Psychology and Neuroscience, Department of Psychosis Studies; South London and Maudsley NHS Foundation Trust<br>McGuire, Philip; King's College London Institute of Psychiatry Psychology and Neuroscience, Department of Psychosis Studies; South London and Maudsley NHS Foundation Trust

Keywords:	Depression & mood disorders < PSYCHIATRY, Schizophrenia & psychotic disorders < PSYCHIATRY, Health informatics < BIOTECHNOLOGY & BIOINFORMATICS, EPIDEMIOLOGY

SCHOLARONE™
Manuscripts

I, the Submitting Author has the right to grant and does grant on behalf of all authors of the Work (as defined in the below author licence), an exclusive licence and/or a non-exclusive licence for contributions from authors who are: i) UK Crown employees; ii) where BMJ has agreed a CC-BY licence shall apply, and/or iii) in accordance with the terms applicable for US Federal Government officers or employees acting as part of their official duties; on a worldwide, perpetual, irrevocable, royalty-free basis to BMJ Publishing Group Ltd ("BMJ") its licensees and where the relevant Journal is co-owned by BMJ to the co-owners of the Journal, to publish the Work in this journal and any other BMJ products and to exploit all rights, as set out in our licence.

The Submitting Author accepts and understands that any supply made under these terms is made by BMJ to the Submitting Author unless you are acting as an employee on behalf of your employer or a postgraduate student of an affiliated institution which is paying any applicable article publishing charge ("APC") for Open Access articles. Where the Submitting Author wishes to make the Work available on an Open Access basis (and intends to pay the relevant APC), the terms of reuse of such Open Access shall be governed by a Creative Commons licence – details of these licences and which Creative Commons licence will apply to this Work are set out in our licence referred to above.

Other than as permitted in any relevant BMJ Author's Self Archiving Policies, I confirm this Work has not been accepted for publication elsewhere, is not being considered for publication elsewhere and does not duplicate material already published. I confirm all authors consent to publication of this Work and authorise the granting of this licence.

For peer review only

Associations of presenting symptoms and subsequent bipolar/psychotic disorder onset in people with unipolar depression: a prospective natural language processing, transdiagnostic, network analysis of electronic health record data

Rashmi Patel*[1,2], Jessica Irving*[1], Aimee Brinn*[1], Matthew Taylor[3], Hitesh Shetty[2], Megan Pritchard[1,2], Robert Stewart[1,2], Paolo Fusar-Poli[1,2], Philip McGuire[1,2]

*Equal contributions to authorship

1. King's College London (Institute of Psychiatry, Psychology and Neuroscience), London, UK

2. South London and Maudsley NHS Foundation Trust, London, UK

3. University of Oxford (Department of Psychiatry), Oxford, UK

Correspondence to:

Rashmi Patel

E-mail: rashmi.patel@kcl.ac.uk

Manuscript word count (excluding references): 4,615

Abstract word count (max 300): 300

Keywords: transdiagnostic, mixed features, depression, bipolar disorder, EHR, NLP

For peer review only - http://bmjopen.bmj.com/site/about/guidelines.xhtml

Competing interests: All authors have completed the ICMJE uniform disclosure form at www.icmje.org/coi_disclosure.pdf and declare: RS has received funding from Janssen, GSK and Takeda outside the submitted work. RP has received funding from Janssen, Induction Healthcare and Holmusk outside the submitted work. The other authors declare no competing interests.

Author contributions: The study was conceived by RP. Data extraction and statistical analysis were performed by JI supervised by RP. Reporting of findings were carried out by RP, JI and AB. All authors (RP, JI, AB, MT, HS, MP, RS, PFP, PM) contributed to study design, manuscript preparation and approved the final version.

Ethics approval: The CRIS data resource received ethical approval as a de-identified dataset for secondary mental health research analyses from Oxfordshire REC C (Ref: 18/SC/0372). The study was approved by the SLaM BRC CRIS oversight committee. Consent is not required to analyse the de-identified dataset for approved research studies. Patients may opt-out of inclusion in the de-identified dataset.

Source of funding: HS, MP, RS and PM have received funding from the National Institute for Health Research (NIHR) Biomedical Research Centre at South London and Maudsley NHS Foundation Trust and King's College London, which also supports the development and maintenance of the BRC Case Register. RP has received funding from a Medical Research Council (MRC) Health Data Research UK Fellowship (MR/S003118/1) and a Starter Grant for Clinical Lecturers (SGL015/1020) supported by the Academy of Medical Sciences, The Wellcome Trust, MRC, British Heart Foundation, Arthritis Research UK, the Royal College of Physicians and Diabetes UK. RS has received funding from a Medical Research Council (MRC) Mental Health Data Pathfinder Award to King's College London, an NIHR Senior Investigator Award and the National Institute for Health Research (NIHR) Applied Research Collaboration South London (NIHR ARC South London) at King's College Hospital NHS Foundation Trust.

Role of funder: The views expressed are those of the authors and not necessarily those of the funders. The funders had no role in the design and conduct of the study; collection, management, analysis, and interpretation of the data; preparation, review, or approval of the manuscript; and decision to submit the manuscript for publication.

Data sharing: The data accessed by CRIS remain within an NHS firewall and governance is provided by a patient-led oversight committee. Access to data is restricted to honorary or substantive employees of the South London and Maudsley NHS Foundation Trust and governed by a local oversight committee who review and approve applications to extract and analyse data for research. Subject to these conditions, data access is encouraged and those interested should contact RS (robert.stewart@kcl.ac.uk), CRIS academic lead.

For peer review only - http://bmjopen.bmj.com/site/about/guidelines.xhtml

For peer review only

Abstract

Objective: To investigate the associations of symptoms of mania and depression with clinical outcomes in people with unipolar depression.

Design: A natural language processing electronic health record study. We used network analysis to determine symptom network structure and multivariable Cox regression to investigate associations with clinical outcomes.

Setting: The South London and Maudsley (SLaM) Clinical Record Interactive Search (CRIS) database.

Participants: All patients presenting with unipolar depression between 1st April 2006 and 31st March 2018.

Exposure:

(i) Symptoms of mania: Elation; Grandiosity; Flight of ideas; Irritability; Pressured speech.
(ii) Symptoms of depression: Disturbed mood; Anhedonia; Guilt; Hopelessness; Helplessness; Worthlessness; Tearfulness; Low energy; Reduced appetite; Weight loss.
(iii) Symptoms of mania or depression (overlapping symptoms): Poor concentration; Insomnia; Disturbed sleep; Agitation; Mood instability.

Main Outcomes:

(i) Psychiatric hospital admission.
(ii) Bipolar or psychotic disorder diagnosis.

Results: Out of 19,707 patients, at least one depression, overlapping or mania symptom was present in 18,998 (96.4%), 15,954 (81.0%), and 4,671 (23.7%) patients respectively. 2,772 (14.1%) patients subsequently developed bipolar or psychotic disorder during the follow-up period. The presence of at least one mania (HR: 2.00, 95% CI 1.85 to 2.16), overlapping symptom (HR: 1.71, 95% CI 1.52 to 1.92) or symptom of depression (HR: 1.31, 95% CI 1.07 to 1.61) were associated with significantly increased risk of onset of a bipolar or psychotic disorder. Mania (HR: 1.95, 95% CI 1.77 to 2.15) and overlapping symptoms (HR: 1.76, 95% CI 1.52 to 2.04) were associated with greater risk for psychiatric hospital admission than symptoms of depression (HR: 1.41, 95% CI 1.06 to 1.88).

Conclusions: The presence of mania or overlapping symptoms in people with unipolar depression is associated with worse clinical outcomes. Symptom-based approaches to defining clinical phenotype may facilitate a more personalised treatment approach and better predict subsequent clinical outcomes than psychiatric diagnosis alone.

For peer review only - http://bmjopen.bmj.com/site/about/guidelines.xhtml

Article summary

Strengths and limitations

- By applying natural language processing (NLP) techniques to electronic health records, we were able to obtain symptom data routinely collected by clinicians for a large sample of 19,707 individuals, permitting investigation into associations between specific symptom domains and clinical outcomes.
- NLP enabled us to detect subgroups of patients with unipolar depression also experiencing symptoms of mania (i.e. depression with mixed features) that cannot be discretely identified through ICD-10 diagnostic classification.
- Network analysis allowed us to identify the degree to which certain symptoms co-occur and influence each other as part of the clinical presentation.
- NLP techniques are unable to achieve 100% precision and recall and so the presence of symptoms in electronic health records may not perfectly reflect the true prevalence of symptoms within the cohort.
- Our outcome data derive from a secondary mental healthcare setting and cannot be generalised to people with unipolar depression that are managed within primary care or in the community.

For peer review only - http://bmjopen.bmj.com/site/about/guidelines.xhtml

Introduction

Unipolar depression is one of the most prevalent mental disorders and can lead to impaired functioning and considerable illness burden.[1] A proportion of people with unipolar depression present with concurrent subthreshold manic/hypomanic symptoms known as "mixed features". These can include elevated mood, grandiosity, pressure of speech, flight of ideas, increased energy, increased risk taking and decreased need for sleep. The estimated prevalence of at least one manic symptom and at least three manic systems among people with unipolar depression is 22-50% and 7-23% respectively.[2] Evidence indicates that unipolar depression with mixed features requires only two or three concomitant manic symptoms to be associated with a more severe and disabling course of illness,[3,4] increased relapse frequency,[3,5] increased risk for suicidality,[5] poorer antidepressant response,[6,7] and greater functional difficulties.[4,5] More recently, attenuated manic and cyclothymic features in young people with unipolar depression have been operationalised by the Semistructured Interview for Bipolar At Risk States. This psychometric interview defines subgroup of clinical high-risk states for developing a first episode of bipolar disorder; pilot findings suggest a promising prognostic accuracy.[8] The clustering of symptoms indicated by the 'SIBARS suggest that aggregation of specific *symptoms* may be useful to detect early phases preceding the onset of bipolar disorder and other severe clinical outcomes. This could theoretically allow early identification of those more likely to develop bipolar disorders and therefore implement preventive approaches that could benefit their outcomes.

Investigating the earlier phases preceding the onset of a bipolar disorder is complicated by the need of prospective designs that represent real world clinical course of this disorder. Electronic health records (EHRs) offer empirical advantages tackling these issues by allowing large scale investigation of pattern of symptoms that may preceded the onset of the disorder. Natural language processing (NLP) is an innovative, automated information extraction technique to investigate the associations of specific symptoms of mental disorders with clinical outcomes using EHR data from mental healthcare.[9–12]

These associations could be further elucidated by adopting network analyses, which investigate the co-occurrence and cross-influence of individual symptoms as part of the clinical presentation. Network analysis has previously demonstrated that the presence of mixed features in people with unipolar depression is associated with poorer response to lurasidone compared to people without mixed features (n=208).[13] A study applying network analysis techniques to mood symptom data obtained through online questionnaires (n=647) and outpatient clinical assessments (n=1,370) demonstrated that symptoms of mania and depression are transdiagnostic and can occur in either unipolar depression or bipolar disorder.[14] However, to the best of our knowledge, there have been no studies which have investigated the relationship between symptoms of mania and depression through network analysis applied to large volumes of EHR data.

We aimed to identify how often symptoms of mania are recorded in the EHR of patients who initially present with unipolar depression, and the association of symptoms of mania with subsequent diagnosis of bipolar or psychotic disorder, and with psychiatric hospital admissions. We represent symptoms of depression and mania graphically using network analysis in a sample of 19,707 patients presenting to a secondary mental healthcare service in South London.

For peer review only - http://bmjopen.bmj.com/site/about/guidelines.xhtml

Methods

Participants and setting

We extracted data from de-identified EHRs for individuals accessing secondary mental health care provided by the South London and Maudsley NHS Foundation Trust (SLaM; London, UK) between 1st April 2006 to 31st March 2018. SLaM holds EHRs for over 500,000 people in receipt of mental healthcare since 2007.[15] The Trust provides community and inpatient mental healthcare within its catchment area covering the London boroughs of Lambeth, Southwark, Croydon and Lewisham. We used the Clinical Record Interactive Search tool (CRIS) to extract and analyse de-identified EHR data from SLaM, with full access to the database population used to create the study population.[15]

Inclusion criterion: all individuals with a primary diagnosis of unipolar depression (eTable 1) recorded in a structured diagnosis EHR field who presented to a SLaM community or inpatient service between 1st April 2006 and 31st March 2018. Follow-up data were obtained from the date of acceptance to the community or inpatient service in which the first recorded diagnosis of unipolar depression was made (the *index date*).

Exclusion criteria: patients with a prior diagnosis of bipolar disorder (ICD-10: F31*), mania (ICD-10: F30*) or psychotic disorder (ICD-10: F1x.5, F2x, F32.3 and F33.3) were excluded. We further excluded patients who went on to receive a diagnosis of psychotic disorder, bipolar disorder or mania within 3 months following their first depression diagnosis. This exclusion criterion was employed to exclude individuals whose first presentation diagnosed as unipolar depression could have represented the initial phase of a bipolar disorder. We excluded patients with a delay of one year or more between the index date and first unipolar depression diagnosis date as the symptoms recorded around the time of presentation may not have represented symptoms associated with the recorded diagnosis of unipolar depression for individuals with a long delay between initial referral and subsequent unipolar depression diagnosis. Patients with no recorded symptoms or missing ethnicity data were also excluded.

Measures

Data were extracted on age at index date, gender, ethnicity, symptoms of depression or mania (including those which could be a feature of either depression or mania), psychiatric hospital admissions (voluntary and involuntary under the UK Mental Health Act) and diagnoses of bipolar disorder (ICD-10: F30/31) or psychotic disorders (ICD-10: F1x.5, F2x, F32.3 and F33.3). Ethnicity was recoded according to categories defined by the UK Office of National Statistics (ONS) as Asian, Black, Mixed, Other and White (eTable 2).

We obtained data for 20 symptoms using NLP tools developed for use on EHR data obtained using CRIS (eTable 3). These symptoms were chosen based on their inclusion in either ICD-10 or DSM-5 diagnostic criteria for unipolar depression and/or mania with the exception of mood instability which is not present in diagnostic criteria for either disorder but was included because it has been previously shown to be an important clinical feature in people with unipolar depression and bipolar disorder.[9] The CRIS NLP tools extract symptom data from unstructured free text clinical documents which record clinical assessment, mental state examination and the agreed treatment plan. Their performance is mainly measured through precision and recall. Precision (positive predictive value) refers to the percentage of true positive sentences identified by the NLP tool of the total positive

For peer review only - http://bmjopen.bmj.com/site/about/guidelines.xhtml

sentences identified by the NLP tool. Recall (sensitivity) refers to the percentage of true positive sentences identified by the NLP tool of the total number of positive sentences. Precision and recall statistics for the NLP applications employed in this as well as further details on their derivation, are provided in the CRIS Natural Language Processing Applications Library.[16]

Symptom data were drawn from a period within three months either side of the index date, as symptoms presenting in this time are more likely to inform the initial diagnosis. We converted each symptom data into dichotomous variables of *present* vs *absent* in a given patient's record. This approach was chosen instead of quantifying the number of times each symptom was recorded in order to minimise confounding by the volume of documentation within a patient's record which may vary depending on illness severity and mode of clinical presentation (e.g. outpatient vs inpatient). We classified symptoms *a priori* into three groups according to ICD-10/DSM-5 diagnostic criteria (see eTable 3): depressive, mania and overlapping symptoms (i.e. symptoms present in ICD-10/DSM-5 classification criteria for both unipolar depression and bipolar disorder).

Follow-up data were obtained from 3 months following the index date for each patient up to 31st March 2021 to ensure predictor symptom data were temporally separate from outcome data. Psychiatric hospitalisation data were obtained as the date of first recorded admission to a SLaM psychiatric hospital during the follow-up period and categorised as (a) any psychiatric hospital admission, and (b) compulsory psychiatric hospital admission under section 2 or 3 of the UK Mental Health Act (MHA). Data on subsequent diagnosis of psychotic disorder or bipolar disorder were obtained from structured diagnostic fields and unstructured free text EHR documents using NLP.[16] We chose to obtain follow-up diagnosis data from both structured and unstructured fields to maximise early identification of onset of these disorders as clinicians typically document emerging diagnoses in unstructured free text prior to recording the diagnosis in the structured EHR fields.

Statistical analysis

We followed the TRANSD ("TRANSD"iagnostic research recommendations in psychiatry) reporting guidelines.[17] All analyses were conducted in R (version 3.6.1). We obtained descriptive statistics on age, gender, ethnicity and the most commonly recorded symptoms and symptom combinations. To investigate demographic associations with recording of manic symptoms among patients diagnosed with unipolar depression, we assessed associations between the presence of at least once manic symptom and at least one overlapping symptom with age, gender and ethnicity through univariate logistic regression.

We used network analysis to visually examine connectivity between depressive and manic symptoms. In a network graph, circles represent nodes (symptom items) and lines represent edges. Each edge denotes a partial correlation between two symptoms (i.e., the correlation between two symptoms when controlling for all other symptoms in the network). Edge thickness represents correlation magnitude. We estimated the network using the Enhanced Least Absolute Shrinkage and Selection Operator (eLASSO) procedure implemented via the *EstimateNetwork* package. The *qgraph* package was used to visualise the network.

Node placement was determined using the Fruchterman-Reingold algorithm, which places the most central nodes visually centre in the network graph.[18] In terms of centrality indices, we calculated *strength, closeness* and *betweenness* defined in eTable 4. We used a case-dropping bootstrapping procedure to assess network stability. Further information on our network analysis approach can be found in the supplementary material (eMethods 1).

For peer review only - http://bmjopen.bmj.com/site/about/guidelines.xhtml

We created dichotomous predictor variables indicating where individuals had at least one reported symptom within each of the following symptom groups: mania, depression, overlapping. We chose to dichotomise these predictors (i.e. the presence of at least one symptom in each group was recorded as “1” and absence of any symptom within a group recorded as “0”) as symptom groups had unequal numbers of constituent symptoms, therefore treating these predictors as continuous would have weighted one symptom group over the other. Additionally, we examined the associations of each individual symptom within the mania and overlapping group with the outcomes described subsequently.

We included three demographic covariates in multivariable analyses to evaluate associations of symptom groups with clinical outcomes: age, gender and ethnicity. Using these covariates and the predictors described above, we estimated Cox proportional hazards regression models (censor date: 31st March 2021) to predict hazard ratios (HR) for risk of the following outcomes:

(i) Receiving a diagnosis of bipolar or psychotic disorder

(ii) Psychiatric hospital admission (any)

(iii) Compulsory psychiatric admission under the UK Mental Health Act (MHA)

(iv) Receiving a diagnosis of bipolar disorder

(v) Receiving a diagnosis of psychotic disorder

Bipolar disorder was defined according to the following ICD-10 diagnostic categories: ICD-10 F30 and F31.

Psychotic disorder was defined according to the following ICD-10 diagnostic categories: ICD-10 F1x.5, F2x, F32.3 and F33.3.

Where a patient was diagnosed with both bipolar disorder and psychotic disorder during the follow-up period, the first recorded diagnosis was considered when assessing outcome (i) listed above.

Patient and public involvement

The study was approved by the SLaM CRIS Oversight Committee which has service user representation. Researchers wishing to access CRIS data can make an application to the Oversight Committee via the CRIS website (https://projects.slam.nhs.uk/research/cris/cris-project-application). The design and completion of approved CRIS projects is supported by a service user advisory group (the CRIS SUCAG) whose members have experience of receiving mental healthcare (or providing care to someone receiving mental healthcare) from SLaM clinical services.[19]

For peer review only - http://bmjopen.bmj.com/site/about/guidelines.xhtml

Results

Descriptive statistics

The final sample comprised 19,707 patients, having excluded 1,206 patients with over a year delay between the index date and unipolar depression diagnosis, 3,407 patients with no documented symptom data, 2,364 patients with missing ethnicity data and a further 7 patients with missing gender data (see eFigure 1).

Demographic and clinical characteristics are reported in Table 1. Our sample was predominantly female (61.9%), of White ethnicity (59.5%), with a mean age (SD) of 38.0 (12.3) years. Recorded symptoms of depression were ascertained in 18,998 patients (96.4%), diagnostically overlapping symptoms in 15,954 patients (81.0%) and symptoms of mania in 4,671 patients (23.7%). Patients with at least one symptom of mania were significantly more likely to be male (OR: 1.15, 95% CI 1.08 to 1.23, $p < 0.001$) and significantly less likely to be of Other ethnicity relative to White ethnicity (OR: 0.85, 95% CI 0.76 to 0.94, $p = 0.006$). Patients with at least one diagnostically overlapping symptom were also significantly more likely to be male (OR: 1.20, 95% CI 1.11 to 1.29, $p < 0.001$), significantly more likely to be of Asian ethnicity relative to White ethnicity (OR: 1.26, 95% CI 1.07 to 1.49, $p = 0.019$).

We extracted a median of 11 (IQR: 5 to 24) symptoms per patient, reported in 6,910 different combinations. There were 642 combinations of symptoms of depression, 32 combinations of overlapping symptoms and 31 combinations of symptoms of mania. We found at least one overlapping symptom recorded for 15,954 patients (81.0%). At least two overlapping symptoms were recorded for 10,119 patients (51.3%) and at least three were recorded for 5,188 patients (26.3%). At least one mania symptom was recorded for 4671 (23.7%) patients. 634 (3.2%) patients had at least two recorded symptoms of mania, whilst three or more symptoms were recorded for 144 (0.73%) patients.

The distribution of ICD-10 unipolar depression diagnoses across the cohort is reported in eTable 5. The most common index diagnosis was F32.1 Moderate depressive episode (n = 5,242, 26.6%).

Network analysis

Figure 1 presents the regularised partial correlation network of symptoms in people with unipolar depression. Symptoms of mania cluster between themselves, and symptoms of depression and some diagnostically overlapping symptoms cluster together (including within-group clustering). Visual inspection of the network graph revealed two diagnostically overlapping symptoms (agitation and mood instability) with strong associations to the principal symptoms of mania.

The resulting network remained stable after dropping up to 33% of cases from the sample. The strongest edges for each symptom are presented in Table 2. The strongest association was between pressured speech and flight of ideas; the second strongest association was between hopelessness and helplessness. Symptom centrality indices (strength, closeness, betweenness and expected influence) are provided in eFigure 2. Agitation was the most central symptom in terms of strength, betweenness and expected influence. Disturbed mood was the most central symptom in terms of closeness. Stability analysis of the centrality indices suggests correlation stability coefficients of 0.67 for Strength, 0.67 for Betweenness and 0.67 for Closeness.[20]

For peer review only - http://bmjopen.bmj.com/site/about/guidelines.xhtml

Diagnostic outcomes

During 166,686.5 person-years of follow-up (mean: 8.45 years, std dev: 15.0 years), 2,772 (14.07%) individuals received a subsequent diagnosis of a psychotic or bipolar disorder; 2,150 (10.91%) received a psychotic disorder diagnosis and 986 (5.00%) received a bipolar disorder diagnosis. The incidence rate (cases per 10,000 person-years risk) was 166.4 (95% CI: 160.2 to 172.7) for diagnosis of psychotic or bipolar disorder, 128.6 (95% CI: 123.2 to 134.2) for diagnosis of psychotic disorder and 59.0 (95% CI: 55.4 to 62.8) for diagnosis of bipolar disorder.

Results of multivariable Cox proportional hazard analyses are reported in Table 3. In decreasing order of risk, individuals with at least one symptom of mania, at least one overlapping symptom had a significantly increased risk of onset of psychotic or bipolar disorder or at least one symptom of depression significantly increased risk for bipolar or psychotic disorder. Pressured speech (HR: 3.07, 95% CI 2.56 to 3.69), grandiosity (HR: 3.04, 95% CI: 2.46 to 3.75), and flight of ideas (HR: 2.83, 95% CI: 2.30 to 3.48) were the individual symptoms most strongly associated with subsequent onset of psychotic or bipolar disorder. Black or Asian ethnicity (relative to White ethnicity) and age were also significant predictors of risk of subsequent diagnosis of bipolar or psychotic disorder.

Cox proportion hazard analyses for risk of subsequent diagnosis of bipolar disorder are reported in eTable 6. In a multivariable analysis, individuals with at least one symptom of mania or at least one overlapping symptom had a significantly increased risk of subsequent bipolar disorder diagnosis. Individual symptoms: grandiosity (HR: 4.36, 95% CI 3.24 to 5.87), elation (HR: 4.21, 95% CI 3.46 to 5.12) and pressured speech (HR: 4.07, 95% CI 3.12 to 5.31) were most strongly associated with subsequent onset of bipolar disorder.

Cox proportion hazard analyses for risk of subsequent diagnosis of psychotic disorder are reported in eTable 7. In decreasing order of risk, individuals with at least one mania symptom, at least one overlapping symptom or at least one depression symptom had a significantly increased risk of onset of psychotic disorder. All individual overlapping/mania symptoms were significantly associated with subsequent psychotic disorder diagnosis. Of these individual symptoms, pressured speech (HR: 2.69, 95% CI: 2.17 to 3.33), flight of ideas (HR: 2.61, 95% CI 2.07 to 3.30), and grandiosity (HR: 2.61, 95% CI: 2.03 to 3.36), were most strongly associated with the onset of psychotic disorder.

Psychiatric hospital admission

There were 1,719 psychiatric admissions, of which 516 were compulsory under the UK MHA. Table 4 presents the results from the Cox regression analyses for risk of psychiatric hospital admission and eTable 8 for risk of compulsory psychiatric hospital admission under the UK MHA. Symptoms of depression, symptoms of mania and overlapping symptoms were all associated with non-compulsory hospitalisation risk. Mania and overlapping symptoms predicted compulsory hospitalisation, whilst depressive symptoms did not. Of the individual overlapping/mania symptoms, pressured speech (HR: 3.02, 95% CI: 2.41 to 3.77), agitation (HR: 2.91, 95% CI: 2.65 to 3.20), and flight of ideas (HR: 2.54, 95% CI: 1.98 to 3.25) were associated with the greatest risk of any psychiatric hospital admission.

Pressured speech (HR: 4.37, 95% CI 3.12 to 6.14), elation (HR: 3.96, 95% CI 2.98 to 5.25) and flight of ideas (HR: 3.80, 95% CI 2.61 to 5.53) were associated with the greatest risk of compulsory psychiatric hospital admission. Black and Asian ethnicity were associated with increased risk of compulsory

For peer review only - http://bmjopen.bmj.com/site/about/guidelines.xhtml

For peer review only

psychiatric hospital admission, compared to White ethnicity. Male gender was associated with increased risk of compulsory psychiatric hospital admission, compared to female gender.

For peer review only

For peer review only - http://bmjopen.bmj.com/site/about/guidelines.xhtml

Discussion

We found that people with unipolar depression have a heterogenous clinical phenotype with a significant number (2,772 during 166,686.5 person-years of follow-up) going on to develop a bipolar or psychotic disorder. In a large sample of 19,707 individuals with a diagnosis of unipolar depression, 23.7% had at least one reported symptom of mania (irritability, elation, grandiosity, pressured speech or flight of ideas), and this subset of patients was most likely to be White and male. Irritability was the most commonly documented symptom of mania. Overall, those with symptoms of mania (i.e. mixed features) had poorer clinical outcomes: symptoms of mania symptoms doubled the risk of psychotic or bipolar disorder and more than doubled the risk of compulsory psychiatric admission. Those with overlapping symptoms were also at increased risk of compulsory psychiatric admission, whilst having at least one symptom of depression did not increase the risk of compulsory psychiatric admission.

While we investigated a total of five symptoms shared by ICD-10 and DSM-5 criteria for unipolar depression and mania, our network analysis revealed that only agitation and mood instability co-occurred with mania symptoms of irritability, elation, pressured speech, flight of ideas and grandiosity. Symptoms of insomnia, disturbed sleep and poor concentration that are shared between unipolar depression and bipolar disorder were not significantly associated with the principal symptoms of mania. This concurs with the findings of a study that examined the joint structure of symptoms of mania and depression in two large samples totalling over 2,100 patients[14] which found symptoms such as increased energy, grandiosity and euphoria represented a latent specific positive activation dimension that was more specific to bipolar disorder. We found that our identified overlapping symptoms of agitation and mood instability were associated with subsequent psychotic or bipolar disorder diagnosis.

Highly central symptoms are most likely to sustain the rest of the network. Betweenness centrality is one of the most useful metrics for detecting nodes that connect one region of a network analysis graph to another. Removal of these nodes may therefore represent an opportunity to destabilise the network. Agitation and mood instability were the two overlapping symptoms that showed the highest betweenness centrality. However, betweenness and closeness show low stability in cross-sectional data, wide confidence intervals, and inconsistency across datasets.[21]

DSM-5 criteria for unipolar depression with mixed features (eTable 9) require, along with a major depressive episode, the presence of at least three of seven symptoms of mania including elation, grandiosity, pressured speech, flight of ideas, risk-taking behaviour and decreased need for sleep. However, this clinical picture is extremely rare in mixed depressive states.[2] Furthermore, the DSM-5 criteria for a diagnosis of unipolar depression with mixed features does not account for psychomotor agitation, irritability and mood instability, which Koukopoulos and Sani describe as ‘core features’ of this clinical presentation.[2] This concurs with our network analysis which finds symptoms of agitation and mood instability co-occur more commonly with symptoms of mania than other overlapping symptoms such as disturbed sleep and poor appetite. Therefore, the DSM-5 criteria for unipolar depression with mixed features exclude patients at risk of substantial functional impairment who may need enhanced support and more tailored invention.[2] Certain treatments such as SSRIs could prove inappropriate for these patients by worsening agitation and increasing the risk of onset of mania[22] and hospitalisation.[2]

Demographic factors also contributed to risk for poor outcomes. Patients of Black and Asian ethnicity were almost three times and two times more likely respectively than White patients to be

For peer review only - http://bmjopen.bmj.com/site/about/guidelines.xhtml

compulsorily admitted to a psychiatric hospital under the UK MHA. Previous studies have shown that Black and Minority Ethnic people are disproportionately likely to be admitted to a psychiatric hospital under the UK MHA.[23]

Strengths

Through our use of EHRs we were able to identify a sample of 19,707 individuals at a scale that would have been infeasible to curate through direct patient recruitment. Furthermore, while the diagnosis of *depressive disorder with mixed features* was introduced in the DSM-5 to capture the phenomenon of depressed patients who exhibit concurrent symptoms of mania or hypomania[3], no analogous diagnosis exists in the ICD-10.

Our approach used automated NLP tools to identify symptoms documented in the EHR allowing us to identify patients for further study. Here, we identified 4,888 patients diagnosed with unipolar depression with symptoms of mania reported in their EHRs. Grouping these patients together with all patients with unipolar depression would have inadequately captured the heterogeneity of the unipolar depression diagnostic construct.[24]

Our approach using EHRs provides sample sizes permissive of more granular analyses into specific symptom domains than in previous studies. The use of NLP allowed us to obtain symptom data that tend to be unrepresented in large scale administrative resources. The symptom NLP algorithms allow more fine-grained definition of item constructs than that of typical depression criteria. Mania symptoms are not routinely elicited in standard depression screening questionnaires, and classification systems sometimes combine opposing poles of disorder into unitary terms (like agitation/psychomotor retardation) when they are likely to reflect different underlying neurobiological processes.[24] Our approach allowed us to extract data on agitation and psychomotor retardation separately.

We followed the TRANSD guidelines for reporting transdiagnostic research in psychiatry.[17] We provided a transparent definition of specific ICD-10 diagnostic codes which were fully detailed and numerated to characterise the cohort of patients with unipolar depression and the outcomes of bipolar or psychotic disorders ("T" and "N" criteria). We reported the primary outcome and the study design and defined the transdiagnostic construct as symptoms overlapping unipolar depression and bipolar disorder and reported the primary outcome of the study (receiving a subsequent bipolar or psychotic disorder diagnosis) and study design (prospective natural language processing EHR study) fulfilling the "R" criterion. We appraised the conceptual framework as across-at least three diagnoses (unipolar depression, bipolar disorders, psychosis) and across two diagnostic spectra (mood disorders and psychotic disorders) fulfilling the "A" criterion.

The ability to identify subgroups of people with unipolar depression and symptoms of mania (as mixed features) would enable the development and evaluation of interventions specifically designed to treat mixed features such as agitation and mood instability which have a significant impact on outcomes and may not be well treated by traditional antidepressant or psychological therapy. Increasingly psychopharmacological products are measured in terms of their impacts on specific symptoms. For example, clinical trials evaluating the efficacy of a novel compound to treat agitation and aggression in emotionally unable personality disorder are currently underway.[25] Promising evidence is emerging of treatments able to target previously difficult to treat symptoms. Lurasidone demonstrates efficacy in the treatment of mixed features in people with bipolar disorder.[26] In a separate network analysis, presence of specific manic symptoms moderated the antidepressant and

For peer review only - http://bmjopen.bmj.com/site/about/guidelines.xhtml

antimanic effects of lurasidone in the treatment of depression with mixed features.[13] For example, patients with symptoms of elevated mood and agitation were less likely than patients without these symptoms to benefit from lurasidone in terms of their MADRS and CGI-S Scores. Conversely, presence of rapid/pressured speech predicted improvement in both mania and depression symptoms with lurasidone. These opposing effects would have been missed in standard approaches that group patients by diagnostic category, thereby treating all symptoms as equally important indicators of prognosis.

Furthermore, it would be useful to evaluate whether the presence of mixed features, and specifically mood instability and agitation, predict risk of onset of mania when treated with antidepressants. It is important to identify early warning signs for bipolar disorder as treatment approaches for bipolar disorder and unipolar depression differ considerably, and antidepressants can increase risk of mania.[22,27,28]

Limitations

NLP techniques are unable to achieve 100% precision and recall and therefore classify information imperfectly.[29] Therefore the prevalence of symptoms in this cohort cannot be taken as the true prevalence of symptoms within the cohort. However, the overall symptom network is unlikely to be affected by incorrect NLP classification as the network was stable even when dropping over 6,700 cases (33% of our sample). As we were limited by the availability of existing algorithms, we were unable to obtain symptom data available for some depression and mania criteria, for example increased appetite or hypersomnia (depression), or impulsivity and hypersexuality (mania).

We cannot infer temporal relations between symptoms as our symptom data derive from one timepoint only. Some nodes measure overlapping constructs such an insomnia and sleep disturbances; these variables were however not strongly correlated, suggesting that clinicians are using these terms to report separate, but related, phenomena. As we limited our study to people presenting with unipolar depression, we were unable to assess the degree to which a transdiagnostic approach using NLP-derived symptom data could be better or worse at predicting future clinical outcomes compared to assessing initial ICD-10 diagnoses (TRANSD criterion “S”).[12] Finally, our data derive from a secondary mental healthcare setting and cannot be generalised to cases of unipolar depression that are managed within primary care. Future external validation studies should be performed as recommended by the TRANSD reporting guidelines (TRANSD criterion “D”).[17]

Conclusions

Our findings suggest that clinicians should screen for mania and diagnostically overlapping symptoms in patients who present with unipolar depression as these could be associated with increased risk of subsequently developing a psychotic or bipolar disorder and be associated with increased likelihood of psychiatric hospital admission.[12] The DSM-5 criteria for depression with mixed features exclude patients at risk of developing a psychotic or bipolar disorder and who may need enhanced support and more tailored inventions to reduce risk of psychiatric hospital admission.[2] Symptom-level approaches to defining clinical phenotype may enable a better understanding of pathophysiology at the individual patient level, facilitate a more personalised treatment approach, and better predict subsequent clinical outcomes.[30]

For peer review only - http://bmjopen.bmj.com/site/about/guidelines.xhtml

For peer review only - http://bmjopen.bmj.com/site/about/guidelines.xhtml

References

1 Patel V, Chisholm D, Parikh R, *et al.* Addressing the burden of mental, neurological, and substance use disorders: key messages from Disease Control Priorities, 3rd edition. *Lancet (London, England)* 2016;**387**:1672–85. doi:10.1016/S0140-6736(15)00390-6

2 Koukopoulos A, Sani G. DSM-5 criteria for depression with mixed features: A farewell to mixed depression. *Acta Psychiatr Scand* Published Online First: 2014. doi:10.1111/acps.12140

3 McIntyre RS, Soczynska JK, Cha DS, *et al.* The prevalence and illness characteristics of DSM-5-defined 'mixed feature specifier' in adults with major depressive disorder and bipolar disorder: Results from the International Mood Disorders Collaborative Project. *J Affect Disord* 2015;**172**:259–64. doi:10.1016/j.jad.2014.09.026

4 Nusslock R, Frank E. Subthreshold bipolarity: diagnostic issues and challenges. *Bipolar Disord* 2011;**13**:587–603. doi:10.1111/j.1399-5618.2011.00957.x

5 Angst J, Cui L, Swendsen J, *et al.* Major depressive disorder with subthreshold bipolarity in the national comorbidity survey replication. *Am J Psychiatry* 2010;**167**:1194–201. doi:10.1176/appi.ajp.2010.09071011

6 Rosenblat JD, Mcintyre RS. Treatment recommendations for DSM-5-defined mixed features. CNS Spectr. 2017;**22**:147–54. doi:10.1017/S1092852916000432

7 Suppes T, Ostacher M. Mixed features in major depressive disorder: Diagnoses and treatments. CNS Spectr. 2017;**22**:155–60. doi:10.1017/S1092852917000256

8 Fusar-Poli P, De Micheli A, Rocchetti M, *et al.* Semistructured Interview for Bipolar At Risk States (SIBARS). *Psychiatry Res* 2018;**264**:302–9. doi:https://doi.org/10.1016/j.psychres.2018.03.074

9 Patel R, Lloyd T, Jackson R, *et al.* Mood instability is a common feature of mental health disorders and is associated with poor clinical outcomes. *BMJ Open* 2015;**5**. doi:10.1136/bmjopen-2014-007504

10 Patel R, Jayatilleke N, Broadbent M, *et al.* Negative symptoms in schizophrenia: a study in a large clinical sample of patients using a novel automated method. *BMJ Open* 2015;**5**. doi:10.1136/bmjopen-2015-007619

11 Jackson RG, Patel R, Jayatilleke N, *et al.* Natural language processing to extract symptoms of severe mental illness from clinical text: The Clinical Record Interactive Search Comprehensive Data Extraction (CRIS-CODE) project. *BMJ Open* 2017;**7**. doi:10.1136/bmjopen-2016-012012

12 Irving J, Patel R, Oliver D, *et al.* Using Natural Language Processing on Electronic Health Records to Enhance Detection and Prediction of Psychosis Risk. *Schizophr Bull* Published Online First: 7 October 2020. doi:10.1093/schbul/sbaa126

13 Goldberg JF, Siu C, Mao Y, *et al.* Major depressive disorder with mixed features and treatment response to lurasidone: A symptom network model. *J Affect Disord* 2020;**277**:1045–54. doi:10.1016/j.jad.2020.08.048

14 Stanton K, Khoo S, Watson D, *et al.* Unique and Transdiagnostic Symptoms of Hypomania/Mania and Unipolar Depression. *Clin Psychol Sci* 2019;**7**:471–87. doi:10.1177/2167702618812725

15 Perera G, Broadbent M, Callard F, *et al.* Cohort profile of the South London and Maudsley NHS Foundation Trust Biomedical Research Centre (SLaM BRC) Case Register: current status and recent enhancement of an Electronic Mental Health Record-derived data resource. *BMJ Open* 2016;**6**. doi:10.1136/bmjopen-2015-008721

For peer review only - http://bmjopen.bmj.com/site/about/guidelines.xhtml

16 CRIS Natural Language Processing Applications Library. https://www.maudsleybrc.nihr.ac.uk/facilities/clinical-record-interactive-search-cris/cris-natural-language-processing/

17 Fusar-Poli P, Solmi M, Brondino N, *et al.* Transdiagnostic psychiatry: a systematic review. *World Psychiatry* 2019;**18**:192–207. doi:https://doi.org/10.1002/wps.20631

18 Fruchterman TMJ, Reingold EM. Graph drawing by force-directed placement. *Softw Pract Exp* Published Online First: 1991. doi:10.1002/spe.4380211102

19 Jewell A, Pritchard M, Barrett K, *et al.* The maudsley biomedical research centre (Brc) data linkage service user and carer advisory group: Creating and sustaining a successful patient and public involvement group to guide research in a complex area. *Res Involv Engagem* 2019;**5**:20. doi:10.1186/s40900-019-0152-4

20 Epskamp S, Borsboom D, Fried EI. Estimating psychological networks and their accuracy: A tutorial paper. *Behav Res Methods* 2018;**50**:195–212. doi:10.3758/s13428-017-0862-1

21 Epskamp S, Kruis J, Marsman M. Estimating psychopathological networks: Be careful what you wish for. *PLoS One* 2017;**12**:e0179891. doi:10.1371/journal.pone.0179891

22 Patel R, Reiss P, Shetty H, *et al.* Do antidepressants increase the risk of mania and bipolar disorder in people with depression? A retrospective electronic case register cohort study. *BMJ Open* 2015;**5**. doi:10.1136/bmjopen-2015-008341

23 Singh SP, Burns T, Tyrer P, *et al.* Ethnicity as a predictor of detention under the Mental Health Act. *Psychol Med* 2014;**44**:997–1004. doi:10.1017/S003329171300086X

24 Fried EI, Nesse RM. Depression sum-scores don't add up: Why analyzing specific depression symptoms is essential. *BMC Med* 2015;**13**:72. doi:10.1186/s12916-015-0325-4

25 Clinical Trials Register.

26 McIntyre RS, Cucchiaro J, Pikalov A, *et al.* Lurasidone in the treatment of bipolar depression with mixed (subsyndromal hypomanic) features: Post hoc analysis of a randomized placebo-controlled trial. *J Clin Psychiatry* 2015;**76**:398–405. doi:10.4088/JCP.14m09410

27 Baldessarini RJ, Faedda GL, Offidani E, *et al.* Antidepressant-associated mood-switching and transition from unipolar major depression to bipolar disorder: A review. *J Affect Disord* 2013;**148**:129–35.

28 Geddes JR, Miklowitz DJ. Treatment of bipolar disorder. Lancet. 2013;**381**:1672–82. doi:10.1016/S0140-6736(13)60857-0

29 Maynard D, Bontcheva K. Natural language processing. In: *Perspectives on Ontology Learning*. 2014. doi:10.4018/ijssoe.2014010105

30 Insel TR, Cuthbert BN, Garvey MA, *et al.* Research domain criteria (RDoC): toward a new classification framework for research on mental disorders. *Am J Psychiatry* 2010;**167**:748–51.

For peer review only - http://bmjopen.bmj.com/site/about/guidelines.xhtml

Table 1: Demographic and clinical characteristics

Characteristic	Value
**Demographics**	
Age (Mean, SD)	38.0 (12.3) years
Female (n, (%))	12,196 (61.5%)
Ethnicity (n, (%))	
Asian	1,133 (5.7%)
Black	3,473 (17.6%)
Mixed	439 (2.2%)
Other	2,494 (12.6%)
White	12,168 (61.7%)
**Depressive symptoms (n (%))**	
Disturbed mood	17,188 (87.2%)
Tearfulness	10,196 (51.7%)
Reduced appetite	7,996 (40.6%)
Hopelessness	6,160 (31.3%)
Low energy	6,159 (31.3%)
Guilt	5,828 (29.6%)
Weight loss	3,778 (19.2%)
Anhedonia	3,514 (17.8%)
Worthlessness	2,560 (13.0%)
Helplessness	2,168 (11.0%)
**Diagnostically overlapping symptoms[1] (n (%))**	
Disturbed sleep	12,271 (62.3%)
Poor concentration	8,056 (40.9%)
Mood instability	5,315 (27.0%)
Agitation	5,074 (25.7%)
Insomnia	3,250 (16.5%)
**Manic symptoms (n (%))**	
Irritability	3,911 (19.8%)
Elation	642 (3.3%)
Pressured speech	348 (1.8%)
Flight of ideas	356 (1.8%)
Grandiosity	261 (1.3%)

For peer review only - http://bmjopen.bmj.com/site/about/guidelines.xhtml

[1] Symptoms that overlap ICD-10/DSM-5 criteria for both unipolar and bipolar depression or mania.

Table 2: Strongest associations for each symptom in decreasing order of strength

Node 1	Node 2	Mean edge weight (95% CI)
Pressured speech	Flight of ideas	1.76 (1.35 to 2.15)
Hopelessness	Helplessness	1.65 (1.52 to 1.75)
Elation	Grandiosity	1.50 (1.11 to 1.87)
Poor appetite	Weight loss	1.11 (0.99 to 1.17)
Grandiosity	Flight of ideas	1.06 (0.53 to 1.68)
Disturbed sleep	Poor appetite	1.08 (1.00 to 1.15)
Flight of ideas	Agitation	0.97 (0.72 to 1.26)
Irritability	Agitation	0.80 (0.72 to 0.89)
Mood instability	Disturbed mood	0.86 (0.74 to 1.01)
Guilt	Worthlessness	0.67 (0.58 to 0.77)
Low energy	Disturbed mood	0.62 (0.49 to 0.75)
Anhedonia	Disturbed mood	0.61 (0.41 to 0.80)
Concentration	Energy	0.57 (0.49 to 0.63)
Insomnia	Anhedonia	0.50 (0.39 to 0.60)
Helplessness	Worthlessness	0.53 (0.41 to 0.65)
Agitation	Disturbed sleep	0.43 (0.35 to 0.52)
Tearfulness	Disturbed mood	0.37 (0.26 to 0.46)
Worthlessness	Disturbed mood	0.31 (0.12 to 0.54)
Weightloss	Disturbed mood	0.02 (-0.09 to 0.09)

For peer review only

For peer review only - http://bmjopen.bmj.com/site/about/guidelines.xhtml

Table 3: Multivariable Cox regression models examining factors associated with subsequent psychotic or bipolar disorder diagnosis

Predictor	Risk of developing psychotic or bipolar disorder (number of events = 2,772)			
	Univariate Hazard Ratio (95% CI)	p value	[a]Multivariable Hazard Ratio (95% CI)	p value
**Demographics**				
Age	1.00 (1.00 to 1.01)	0.209	1.00 (1.00 to 1.01)	0.025
Male gender	1.01 (0.93 to 1.09)	0.843	1.03 (0.95 to 1.11)	0.489
Ethnicity				
White	*Ref*	*Ref*	*Ref*	*Ref*
Asian	1.42 (1.22 to 1.64)	<0.001***	1.44 (1.24 to 1.67)	<0.001***
Black	1.52 (1.39 to 1.67)	<0.001***	1.55 (1.41 to 1.70)	<0.001***
Mixed	1.07 (0.83 to 1.39)	0.644	1.10 (0.85 to 1.43)	0.495
Other	1.03 (0.90 to 1.17)	0.692	1.04 (0.91 to 1.18)	0.561
**Symptom groups**				
Mania (≥ 1 symptom)	1.98 (1.84 to 2.15)	<0.001***	2.00 (1.85 to 2.16)	<0.001***
Overlapping (≥ 1 symptom)	1.73 (1.54 to 1.94)	<0.001***	1.71 (1.52 to 1.92)	<0.001***
Depression (≥ 1 symptom)	1.31 (1.07 to 1.60)	0.012	1.31 (1.07 to 1.61)	0.01
**Individual symptoms**				
Irritability	1.85 (1.70 to 2.00)	<0.001***	1.85 (1.71 to 2.01)	<0.001***
Elation	2.55 (2.21 to 2.95)	<0.001***	2.65 (2.29 to 3.07)	<0.001***
Pressured speech	3.05 (2.54 to 3.66)	<0.001***	3.07 (2.56 to 3.69)	<0.001***
Flight of ideas	2.83 (2.31 to 3.48)	<0.001***	2.83 (2.30 to 3.48)	<0.001***
Grandiosity	2.99 (2.42 to 3.69)	<0.001***	3.04 (2.46 to 3.75)	<0.001***
Disturbed sleep	1.52 (1.40 to 1.65)	<0.001***	1.49 (1.37 to 1.62)	<0.001***
Poor concentration	1.50 (1.39 to 1.61)	<0.001***	1.48 (1.38 to 1.60)	<0.001***
Mood instability	1.74 (1.61 to 1.88)	<0.001***	1.79 (1.66 to 1.93)	<0.001***
Agitation	2.27 (2.11 to 2.45)	<0.001***	2.29 (2.12 to 2.47)	<0.001***
Insomnia	1.47 (1.35 to 1.61)	<0.001***	1.48 (1.35 to 1.62)	<0.001***

[a]Multivariable analysis adjusted for age, gender and ethnicity.

For peer review only - http://bmjopen.bmj.com/site/about/guidelines.xhtml

For peer review only

For peer review only - http://bmjopen.bmj.com/site/about/guidelines.xhtml

Table 4: Multivariable Cox proportional hazards regression examining factors associated with psychiatric hospital admission

Predictor	Risk of psychiatric hospital admission (number of events = 1,719)			
	Univariate Hazard Ratio (95% CI)	p value	[a]Multivariable Hazard Ratio (95% CI)	p value
**Demographics**				
Age	1.00 (1.00 to 1.00)	0.666	1.00 (1.00 to 1.00)	0.902
Male gender	1.28 (1.17 to 1.41)	<0.001***	1.32 (1.20 to 1.46)	<0.001***
Ethnicity				
White	*Ref*		*Ref*	*Ref*
Asian	0.98 (0.80 to 1.21)	<0.001***	0.99 (0.80 to 1.22)	0.902
Black	1.25 (1.11 to 1.40)	<0.001***	1.30 (1.15 to 1.46)	<0.001***
Mixed	1.02 (0.74 to 1.41)	0.884	1.06 (0.77 to 1.46)	0.769
Other	0.58 (0.48 to 0.69)	<0.001***	0.63 (0.52 to 0.75)	<0.001***
**Symptom groups**				
Mania (≥ 1 symptom)	1.97 (1.79 to 2.18)	<0.001***	1.95 (1.77 to 2.15)	<0.001***
Overlapping (≥ 1 symptom)	1.79 (1.55 to 2.07)	<0.001***	1.76 (1.52 to 2.04)	<0.001***
Depression (≥ 1 symptom)	1.38 (1.03 to 1.83)	0.033	1.41 (1.06 to 1.88)	0.021
**Individual symptoms**				
Irritability	1.92 (1.73 to 2.13)	<0.001***	1.90 (1.71 to 2.10)	<0.001***
Elation	2.51 (2.09 to 3.02)	<0.001***	2.56 (2.13 to 3.07)	<0.001***
Pressured speech	3.01 (2.40 to 3.76)	<0.001***	3.02 (2.41 to 3.77)	<0.001***
Flight of ideas	2.61 (2.04 to 3.35)	<0.001***	2.54 (1.98 to 3.25)	<0.001***
Grandiosity	2.73 (2.10 to 3.57)	<0.001***	2.58 (1.98 to 3.36)	<0.001***
Disturbed sleep	1.72 (1.55 to 1.92)	<0.001***	1.69 (1.52 to 1.88)	<0.001***
Poor concentration	1.55 (1.41 to 1.71)	<0.001***	1.54 (1.40 to 1.69)	<0.001***
Mood instability	1.86 (1.68 to 2.04)	<0.001***	1.88 (1.71 to 2.08)	<0.001***
Agitation	2.99 (2.72 to 3.29)	<0.001***	2.91 (2.65 to 3.20)	<0.001***
Insomnia	1.69 (1.52 to 1.89)	<0.001***	1.68 (1.51 to 1.88)	<0.001***

[a]Multivariable analysis adjusted for age, gender and ethnicity.

For peer review only

For peer review only

For peer review only - http://bmjopen.bmj.com/site/about/guidelines.xhtml

Figure legends

Figure 1

Circles represent nodes (symptom items) and lines represent edges. Each edge denotes a partial correlation between two symptoms (i.e., the correlation between two symptoms when controlling for all other symptoms in the network); edge thickness represents correlation magnitude. We used the Fruchterman-Reingold algorithm, which places the most central nodes visually centre in the network graph, to determine node placement.[18]

For peer review only

For peer review only - http://bmjopen.bmj.com/site/about/guidelines.xhtml

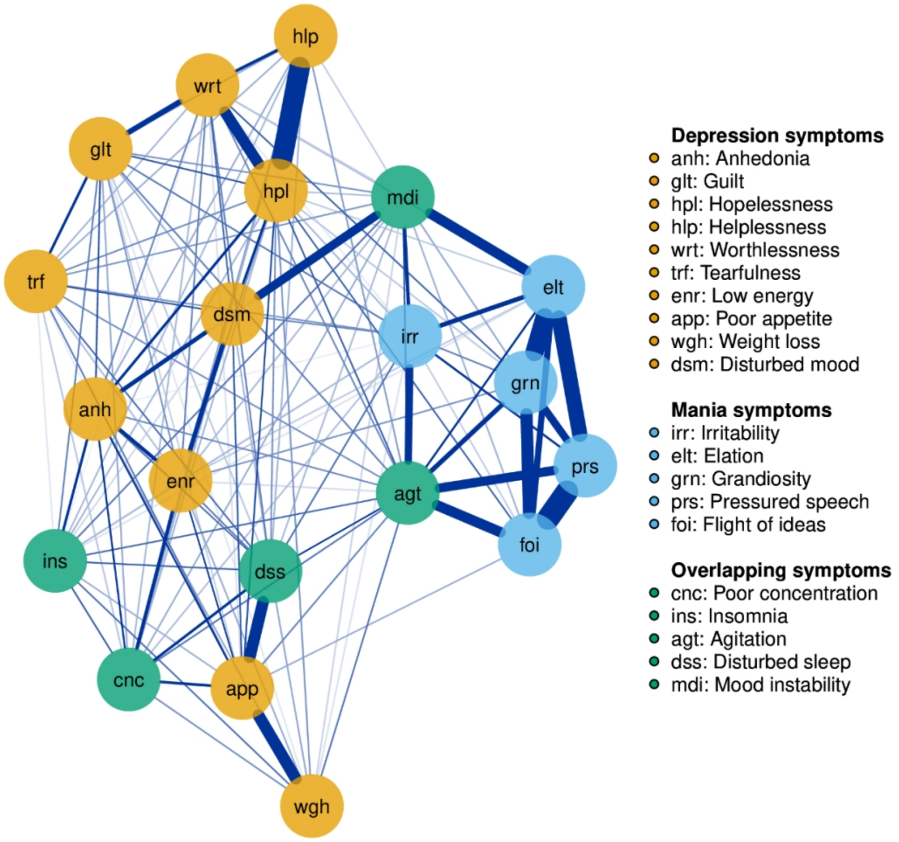

Circles represent nodes (symptom items) and lines represent edges. Each edge denotes a partial correlation between two symptoms (i.e., the correlation between two symptoms when controlling for all other symptoms in the network); edge thickness represents correlation magnitude. We used the Fruchterman-Reingold algorithm, which places the most central nodes visually centre in the network graph, to determine node placement.[18]

159x159mm (220 x 220 DPI)

SUPPLEMENTARY MATERIAL

Associations of presenting symptoms and subsequent bipolar/psychotic disorder onset in people with unipolar depression: a prospective natural language processing, transdiagnostic, network analysis of electronic health record data

Table of Contents

Table of Contents 1

eMethods 1: Network analysis 2

eTable 1: Diagnosis inclusion criteria 2

eTable 2: Ethnicity classification 3

eTable 3: Categorisation of symptoms 4

eTable 4: Centrality definitions[2] 4

eTable 5: Frequency of recorded ICD-10 unipolar depression diagnoses 5

eTable 6: Multivaribale Cox regression models examining factors associated with subsequent bipolar disorder diagnosis 6

eTable 7: Multivariable Cox regression models examining factors associated with subsequent psychotic disorder diagnosis 7

eTable 8: Multivariable Cox proportional hazards regression examining factors associated with compulsory psychiatric hospital admission 8

eTable 9: DSM-5 mixed features specifier of depression[3] 9

eFigure 1: Cohort definition and missing data 10

eFigure 2: Centrality indices for overall network 11

References 12

For peer review only - http://bmjopen.bmj.com/site/about/guidelines.xhtml

eMethods 1: Network analysis

The Enhanced Least Absolute Shrinkage and Selection Operator (eLASSO) procedure implemented via the *EstimateNetwork* package is based on the Ising model which is indicated for analysis of binary data. The method combines L1-regularised logistic regression with model selection using a tuning parameter selected by minimizing the Extended Bayesian Information Criterion (EBIC) to achieve sparsity. Sparse symptom networks have much fewer links between symptoms than the possible maximum number of links within that network (where every symptom would be connected). The use of LASSO regularization creates a more parsimonious network model that returns only the most prominent associations between symptoms, and excludes spurious associations. This in turn maximises model interpretability.[1]

In terms of centrality indices, we calculated *strength, closeness* and *betweenness* defined in eTable 4. Centrality indices measure how likely a symptom is to mediate associations between other symptoms or be strongly connected to other symptoms, regardless of that symptom's prevalence. In brief, nodes of high *strength* are likely to be associated with most members of the network, low *closeness* centrality indicates that a node is closely connected with most other nodes in the network (less likely to be peripheral) and *betweenness* centrality indicates the amount of influence a node has over the flow of information in a graph.[2]

We used a case-dropping bootstrapping procedure to assess network stability. In this procedure, the network is reconstructed, and symptom centrality indices are recalculated over multiple iterations in which cases are dropped in increasing proportions. This generates a Correlation Stability Coefficient, which indicates the maximum proportion of cases that can be dropped from the sample without causing the correlation between the original and bootstrapped centrality values to drop below r = 0.7. The network is deemed to be stable if the correlation stability coefficient survives above 0.5.[1]

eTable 1: Diagnosis inclusion criteria

ICD-10 code	Diagnosis
F32 – Depressive episode	F32.9 - Depressive episode, unspecified<br>F32.2 - Severe depressive episode without psychotic symptoms<br>F32.0 - Mild depressive episode<br>F32.1 - Moderate depressive episode<br>F32.8 - Other depressive episodes
F33 – Recurrent depressive disorder	F33.1 - Recurrent depressive disorder, current episode moderate<br>F33.4 - Recurrent depressive disorder, currently in remission<br>F33.0 - Recurrent depressive disorder, current episode mild<br>F33.8 - Other recurrent depressive disorders<br>F33.9 - Recurrent depressive disorder, unspecified
F41 – Anxiety disorder	F41.2 - Mixed anxiety and depressive disorder

Note: All diagnoses were drawn from the structured field Primary Diagnosis *in EHR*

For peer review only - http://bmjopen.bmj.com/site/about/guidelines.xhtml

eTable 2: Ethnicity classification

Ethnic group	Ethnicity as recorded in patient electronic health records
Black	Black or Black British - African
	Black or Black British - Caribbean
	Black or Black British - Any other Black background
White	White - British
	White - Irish
	White - Any other White background
Asian	Asian or Asian British - Bangladeshi
	Asian or Asian British - Indian
	Asian or Asian British - Pakistani
	Asian or Asian British - Any other Asian background
	Other Ethnic Groups – Chinese
Mixed	Mixed - White and Asian
	Mixed - White and Black African
	Mixed - White and Black Caribbean
	Mixed - Any other mixed background
Other	Other Ethnic Groups - Any other ethnic group
Missing	Not Stated
	Not Recorded

For peer review only

For peer review only - http://bmjopen.bmj.com/site/about/guidelines.xhtml

eTable 3: Categorisation of symptoms

Depressive symptoms	Overlapping symptoms	Mania symptoms
• Disturbed mood	• Poor concentration	• Irritability
• Anhedonia	• Insomnia	• Elation
• Guilt	• Agitation	• Grandiosity
• Weight loss	• Disturbed sleep	• Flight of ideas
• Reduced appetite	• Mood instability	• Pressured speech
• Hopelessness		
• Helplessness		
• Worthlessness		
• Tearfulness		
• Low energy		

eTable 4: Centrality definitions[2]

Centrality	Calculation	Interpretation
**Betweenness**	The percentage of shortest paths that must go through that specific node.	Describes the amount of influence a node has over the flow of information in a graph. Useful to find nodes that bridge from one part of a graph to another.
**Closeness**	The average distance between one node and all other nodes in the network (by taking the inverse of each edge weight).	Low closeness centrality indicates that node is closely connected with most other nodes, therefore it can spread information most efficiently through a network.
**Strength**	The sum of all absolute edge weights a node is directly connected to.	Likely to be involved with most members of the network.

For peer review only - http://bmjopen.bmj.com/site/about/guidelines.xhtml

eTable 5: Frequency of recorded ICD-10 unipolar depression diagnoses

ICD-10 Code	ICD-10 Diagnosis	n
F32.1	Moderate depressive episode	5,242
F32	Depressive episode	3,998
F41.2	Mixed anxiety and depressive disorder	2,137
F32.0	Mild depressive episode	2,043
F32.9	Depressive episode, unspecified	1,424
F33.1	Recurrent depressive disorder, current episode moderate	1,257
F32.2	Severe depressive episode without psychotic symptoms	1,231
F33	Recurrent depressive disorder	1,123
F33.0	Recurrent depressive disorder, current episode mild	408
F32.8	Other depressive episodes	348
F33.4	Recurrent depressive disorder, currently in remission	326
F33.9	Recurrent depressive disorder, unspecified	276
F33.8	Other recurrent depressive disorders	67

For peer review only - http://bmjopen.bmj.com/site/about/guidelines.xhtml

eTable 6: Multivaribale Cox regression models examining factors associated with subsequent bipolar disorder diagnosis

Predictor	Risk of developing bipolar disorder (number of events = 986)			
	**Univariate Hazard Ratio (95% CI)**	**p value**	**[a]Multivariable Hazard Ratio (95% CI)**	**p value**
**Demographics**				
Age (years)	0.99 (0.98 to 0.99)	<0.001***	0.99 (0.98 to 0.99)	<0.001***
Male gender	0.73 (0.64 to 0.84)	<0.001***	0.77 (0.67 to 0.88)	<0.001***
Ethnicity				
White	*Ref*		*Ref*	*Ref*
Asian	0.86 (0.64 to 1.16)	0.356	0.82 (0.61 to 1.10)	0.229
Black	1.04 (0.88 to 1.22)	0.671	0.96 (0.82 to 1.14)	0.654
Mixed	1.35 (0.93 to 1.95)	0.137	1.21 (0.84 to 1.76)	0.331
Other	0.94 (0.75 to 1.18)	0.621	0.91 (0.72 to 1.14)	0.414
**Symptom groups**				
Mania (≥ 1 symptom)	2.38 (2.10 to 2.71)	<0.001***	2.39 (2.11 to 2.72)	<0.001***
Overlapping (≥ 1 symptom)	1.43 (1.19 to 1.71)	<0.001***	1.45 (1.21 to 1.74)	<0.001***
Depression (≥ 1 symptom)	1.55 (1.09 to 2.19)	0.021	1.45 (1.02 to 2.05)	0.050
**Individual symptoms**				
Irritability	1.99 (1.74 to 2.28)	<0.001***	1.99 (1.74 to 2.28)	<0.001***
Elation	4.21 (3.46 to 5.11)	<0.001***	4.21 (3.46 to 5.12)	<0.001***
Pressured speech	3.87 (2.97 to 5.05)	<0.001***	4.07 (3.12 to 5.31)	<0.001***
Flight of ideas	3.78 (2.75 to 5.20)	<0.001***	3.90 (2.84 to 5.36)	<0.001***
Grandiosity	4.14 (3.08 to 5.56)	<0.001***	4.36 (3.24 to 5.87)	<0.001***
Disturbed sleep	1.13 (0.99 to 1.28)	0.089	1.15 (1.00 to 1.31)	0.054
Poor concentration	1.42 (1.25 to 1.61)	<0.001***	1.43 (1.26 to 1.62)	<0.001***
Mood instability	2.08 (1.83 to 2.36)	<0.001***	2.03 (1.79 to 2.30)	<0.001***
Agitation	2.19 (1.93 to 2.49)	<0.001***	2.31 (2.03 to 2.63)	<0.001***
Insomnia	1.52 (1.32 to 1.77)	<0.001***	1.54 (1.33 to 1.78)	<0.001***

[a]Multivariable analysis adjusted for age, gender and ethnicity.

For peer review only

eTable 7: Multivariable Cox regression models examining factors associated with subsequent psychotic disorder diagnosis

Predictor	Risk of developing psychotic disorder (number of events = 2,150)			
	Univariate Hazard Ratio (95% CI)	p value	[a]Multivariable Hazard Ratio (95% CI)	p value
**Demographics**				
Age (years)	1.01 (1.00 to 1.01)	<0.001***	1.01 (1.01 to 1.01)	<0.001***
Male gender	1.10 (1.01 to 1.20)	0.036	1.12 (1.03 to 1.23)	0.01
Ethnicity				
White	*Ref*		*Ref*	*Ref*
Asian	1.73 (1.47 to 2.03)	<0.001***	1.80 (1.53 to 2.11)	<0.001***
Black	1.83 (1.65 to 2.02)	<0.001***	1.92 (1.73 to 2.12)	<0.001***
Mixed	0.93 (0.67 to 1.29)	0.671	1.00 (0.72 to 1.39)	0.999
Other	1.03 (0.89 to 1.20)	0.671	1.06 (0.92 to 1.23)	0.448
**Symptom groups**				
Mania (≥ 1 symptom)	1.87 (1.71 to 2.05)	<0.001***	1.89 (1.73 to 2.07)	<0.001***
Overlapping (≥ 1 symptom)	1.85 (1.62 to 2.11)	<0.001***	1.81 (1.59 to 2.07)	<0.001***
Depression (≥ 1 symptom)	1.29 (1.02 to 1.63)	0.038	1.32 (1.04 to 1.67)	0.023
**Individual symptoms**				
Irritability	1.80 (1.64 to 1.98)	<0.001***	1.82 (1.65 to 1.99)	<0.001***
Elation	2.04 (1.71 to 2.44)	<0.001***	2.17 (1.81 to 2.60)	<0.001***
Pressured speech	2.67 (2.16 to 3.32)	<0.001***	2.69 (2.17 to 3.33)	<0.001***
Flight of ideas	2.63 (2.08 to 3.32)	<0.001***	2.61 (2.07 to 3.30)	<0.001***
Grandiosity	2.59 (2.02 to 3.33)	<0.001***	2.61 (2.03 to 3.36)	<0.001***
Disturbed sleep	1.71 (1.56 to 1.88)	<0.001***	1.65 (1.50 to 1.81)	<0.001***
Poor concentration	1.56 (1.44 to 1.70)	<0.001***	1.54 (1.42 to 1.68)	<0.001***
Mood instability	1.62 (1.48 to 1.76)	<0.001***	1.70 (1.55 to 1.85)	<0.001***
Agitation	2.42 (2.22 to 2.63)	<0.001***	2.41 (2.21 to 2.63)	<0.001***
Insomnia	1.49 (1.35 to 1.65)	<0.001***	1.50 (1.35 to 1.65)	<0.001***

[a]Multivariable analysis adjusted for age, gender and ethnicity.

For peer review only

eTable 8: Multivariable Cox proportional hazards regression examining factors associated with compulsory psychiatric hospital admission

Predictor	Risk of compulsory psychiatric hospital admission under the UK Mental Health Act (number of events = 516)			
	Univariate Hazard Ratio (95% CI)	p value	[a]Multivariable Hazard Ratio (95% CI)	p value
**Demographics**				
Age (years)	1.00 (0.99 to 1.01)	0.91	1.00 (0.99 to 1.01)	0.794
Male gender	1.23 (1.03 to 1.46)	0.029	1.34 (1.12 to 1.60)	0.002
Ethnicity				
White	*Ref*	*Ref*	*Ref*	*Ref*
Asian	1.63 (1.16 to 2.29)	0.007	1.65 (1.17 to 2.32)	0.006
Black	2.36 (1.95 to 2.86)	<0.001***	2.47 (2.03 to 3.00)	<0.001***
Mixed	1.22 (0.67 to 2.23)	0.560	1.27 (0.70 to 2.33)	0.465
Other	0.72 (0.51 to 1.02)	0.078	0.73 (0.51 to 1.03)	0.084
**Symptom groups**				
Mania (≥ 1 symptom)	2.29 (1.92 to 2.73)	<0.001***	2.28 (1.92 to 2.72)	<0.001***
Overlapping (≥ 1 symptom)	1.68 (1.29 to 2.19)	<0.001***	1.62 (1.25 to 2.11)	0.001
Depression (≥ 1 symptom)	0.99 (0.63 to 1.54)	0.950	1.00 (0.64 to 1.57)	0.999
**Individual symptoms**				
Irritability	2.29 (1.91 to 2.74)	<0.001***	2.28 (1.90 to 2.73)	<0.001***
Elation	3.64 (2.75 to 4.83)	<0.001***	3.96 (2.98 to 5.25)	<0.001***
Pressured speech	4.41 (3.14 to 6.19)	<0.001***	4.37 (3.12 to 6.14)	<0.001***
Flight of ideas	3.91 (2.69 to 5.69)	<0.001***	3.80 (2.61 to 5.53)	<0.001***
Grandiosity	3.83 (2.54 to 5.77)	<0.001***	3.59 (2.38 to 5.41)	<0.001***
Disturbed sleep	1.55 (1.28 to 1.87)	<0.001***	1.47 (1.21 to 1.78)	<0.001***
Poor concentration	1.48 (1.24 to 1.76)	<0.001***	1.45 (1.22 to 1.73)	<0.001***
Mood instability	1.84 (1.54 to 2.19)	<0.001***	1.92 (1.61 to 2.29)	<0.001***
Agitation	3.75 (3.15 to 4.46)	<0.001***	3.69 (3.10 to 4.39)	<0.001***
Insomnia	1.50 (1.2 to 1.84)	<0.001***	1.51 (1.23 to 1.86)	<0.001***

[a]Multivariable analysis adjusted for age, gender and ethnicity.

For peer review only

eTable 9: DSM-5 mixed features specifier of depression[3]

**Depression with mixed features specifier**
A. At least three of the following manic/hypomanic symptoms are present nearly every day during the majority of days of a major depressive episode:<br>1. Elevated, expansive mood<br>2. Inflated self-esteem or grandiosity<br>3. More talkative than usual or pressure to keep talking<br>4. Flight of ideas or subjective experience that thoughts are racing<br>5. Increase in energy or goal-directed activity (either socially, at work or school, or sexually)<br>6. Increased or excessive involvement in activities that have a high potential for painful consequences<br>7. Decreased need for sleep (feeling rested despite sleeping less than usual; to be contrasted with insomnia)<br><br>B. Mixed symptoms are observable by others and represent a change from the person's usual behaviour.<br>C. For individuals whose symptoms meet full criteria for either mania or hypomania, the diagnosis should be bipolar I or bipolar II disorder.<br>D. The mixed symptoms are not attributable to the physiological effects of a substance (e.g., a drug of abuse, a medication or other treatment).<br><br>**Note:** Mixed features associated with a major depressive episode have been found to be a significant risk factor for the development of bipolar I or bipolar II disorder. As a result, it is clinically useful to note the presence of this specifier for treatment planning and monitoring of response to treatment.

For peer review only - http://bmjopen.bmj.com/site/about/guidelines.xhtml

eFigure 1: Cohort definition and missing data

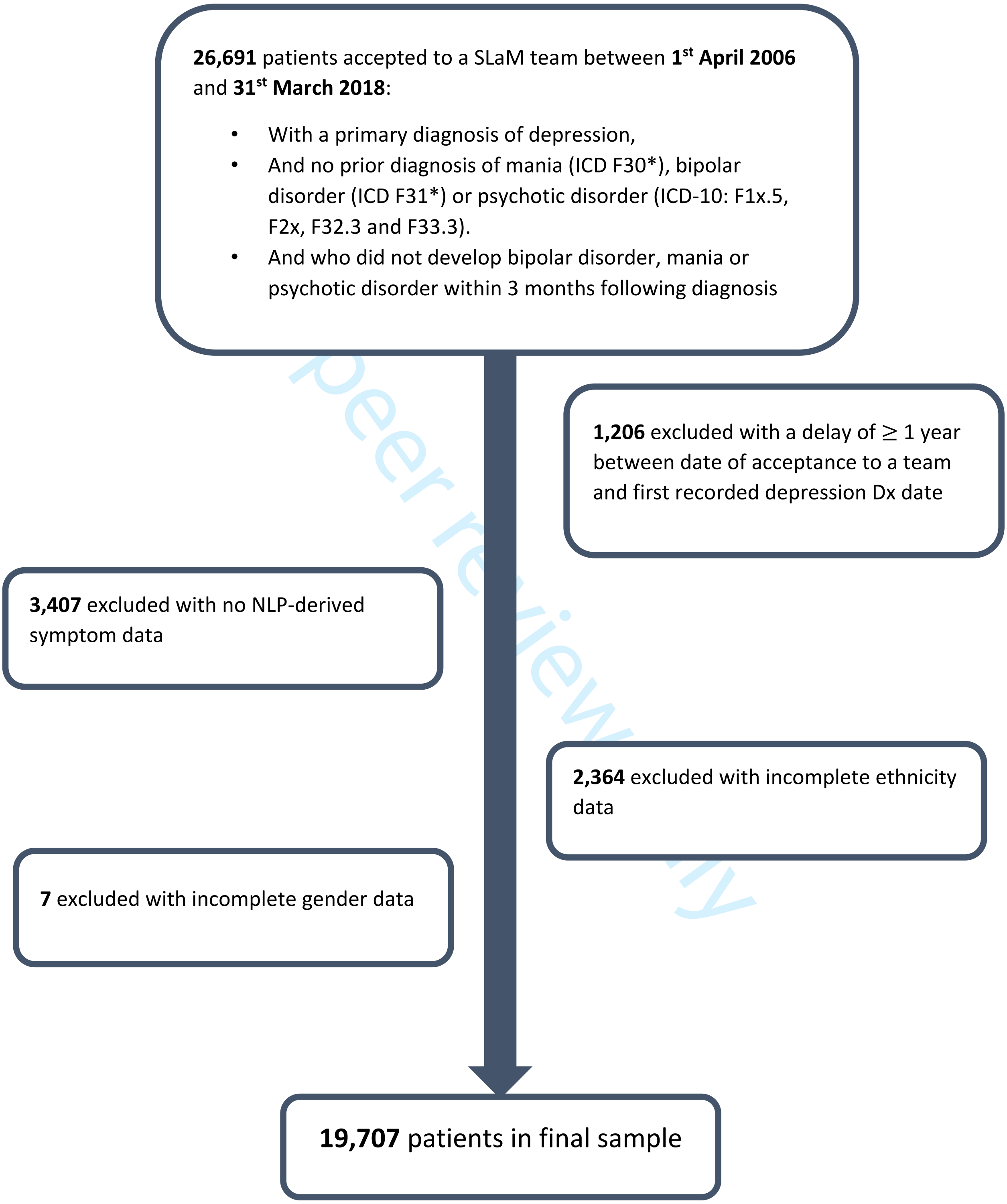

For peer review only - http://bmjopen.bmj.com/site/about/guidelines.xhtml

eFigure 2: Centrality indices for overall network

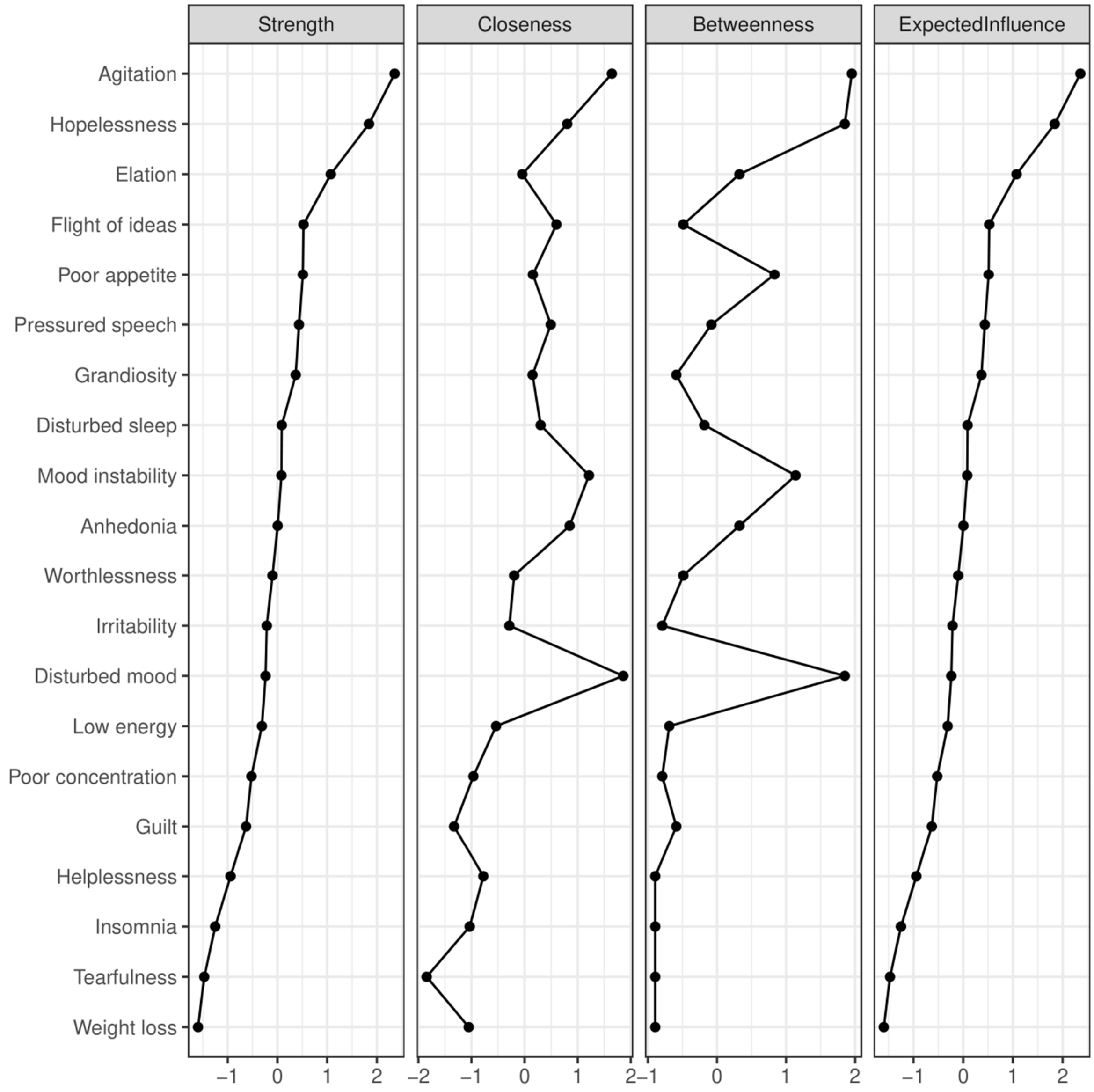

Note: Centrality indices are reported as standardised z scores to facilitate comparison.

References

1 Epskamp S, Borsboom D, Fried EI. Estimating psychological networks and their accuracy: A tutorial paper. *Behav Res Methods* 2018;**50**:195–212. doi:10.3758/s13428-017-0862-1

2 Golbeck J. *Analyzing the Social Web*. Elsevier Inc. 2013. doi:10.1016/C2012-0-00171-8

3 American Psychological Association. *Diagnostic and statistical manual of mental disorders : DSM-5*. Fifth edition. Arlington, VA : American Psychiatric Publishing, [2013] ©2013 2013. https://search.library.wisc.edu/catalog/9911111397702121

For peer review only

The RECORD statement – checklist of items, extended from the STROBE statement, that should be reported in observational studies using routinely collected health data.

	Item No.	STROBE items	Location in manuscript where items are reported	RECORD items	Location in manuscript where items are reported
**Title and abstract**					
	1	(a) Indicate the study's design with a commonly used term in the title or the abstract (b) Provide in the abstract an informative and balanced summary of what was done and what was found	Pages 1 and 3	RECORD 1.1: The type of data used should be specified in the title or abstract. When possible, the name of the databases used should be included.<br><br>RECORD 1.2: If applicable, the geographic region and timeframe within which the study took place should be reported in the title or abstract.<br><br>RECORD 1.3: If linkage between databases was conducted for the study, this should be clearly stated in the title or abstract.	Page 3<br><br>Page 3<br><br>N/A
**Introduction**					
Background rationale	2	Explain the scientific background and rationale for the investigation being reported	Page 5		
Objectives	3	State specific objectives, including any prespecified hypotheses	Page 5		
**Methods**					
Study Design	4	Present key elements of study design early in the paper	Page 6		
Setting	5	Describe the setting, locations, and relevant dates, including periods of recruitment, exposure, follow-up, and data collection	Page 6		

For peer review only

Participants	6	*(a) Cohort study* - Give the eligibility criteria, and the sources and methods of selection of participants. Describe methods of follow-up<br>*Case-control study* - Give the eligibility criteria, and the sources and methods of case ascertainment and control selection. Give the rationale for the choice of cases and controls<br>*Cross-sectional study* - Give the eligibility criteria, and the sources and methods of selection of participants<br><br>*(b) Cohort study* - For matched studies, give matching criteria and number of exposed and unexposed<br>*Case-control study* - For matched studies, give matching criteria and the number of controls per case	Page 6<br><br>N/A – No matching	RECORD 6.1: The methods of study population selection (such as codes or algorithms used to identify subjects) should be listed in detail. If this is not possible, an explanation should be provided.<br><br>RECORD 6.2: Any validation studies of the codes or algorithms used to select the population should be referenced. If validation was conducted for this study and not published elsewhere, detailed methods and results should be provided.<br><br>RECORD 6.3: If the study involved linkage of databases, consider use of a flow diagram or other graphical display to demonstrate the data linkage process, including the number of individuals with linked data at each stage.	Page 6<br><br>Page 7<br><br>N/A – No linkage
Variables	7	Clearly define all outcomes, exposures, predictors, potential confounders, and effect modifiers. Give diagnostic criteria, if applicable.	Pages 6 to 7	RECORD 7.1: A complete list of codes and algorithms used to classify exposures, outcomes, confounders, and effect modifiers should be provided. If these cannot be reported, an explanation should be provided.	Pages 6 to 7
Data sources/ measurement	8	For each variable of interest, give sources of data and details of methods of assessment (measurement).<br>Describe comparability of assessment methods if there is more than one group	Pages 6 to 7		

For peer review only

Bias	9	Describe any efforts to address potential sources of bias	Pages 6 to 7		
Study size	10	Explain how the study size was arrived at	Page 9		
Quantitative variables	11	Explain how quantitative variables were handled in the analyses. If applicable, describe which groupings were chosen, and why	Page 8		
Statistical methods	12	(a) Describe all statistical methods, including those used to control for confounding	Pages 7 to 8		
		(b) Describe any methods used to examine subgroups and interactions	Page 6		
		(c) Explain how missing data were addressed	Page 6		
		(d) *Cohort study* - If applicable, explain how loss to follow-up was addressed	N/A		
		Case-control study - If applicable, explain how matching of cases and controls was addressed	N/A		
		Cross-sectional study - If applicable, describe analytical methods taking account of sampling strategy	N/A		
		(e) Describe any sensitivity analyses	N/A		
Data access and cleaning methods		..		RECORD 12.1: Authors should describe the extent to which the investigators had access to the database population used to create the study population.	Page 6

For peer review only

				RECORD 12.2: Authors should provide information on the data cleaning methods used in the study.	Page 6
Linkage		..		RECORD 12.3: State whether the study included person-level, institutional-level, or other data linkage across two or more databases. The methods of linkage and methods of linkage quality evaluation should be provided.	N/A – No linkage
**Results**					
Participants	13	(a) Report the numbers of individuals at each stage of the study (*e.g.*, numbers potentially eligible, examined for eligibility, confirmed eligible, included in the study, completing follow-up, and analysed)<br>(b) Give reasons for non-participation at each stage.<br>(c) Consider use of a flow diagram	Page 9<br><br>Page 9<br><br>eFigure 1	RECORD 13.1: Describe in detail the selection of the persons included in the study (*i.e.,* study population selection) including filtering based on data quality, data availability and linkage. The selection of included persons can be described in the text and/or by means of the study flow diagram.	Page 9 and eFigure 1
Descriptive data	14	(a) Give characteristics of study participants (*e.g.*, demographic, clinical, social) and information on exposures and potential confounders<br>(b) Indicate the number of participants with missing data for each variable of interest<br>(c) *Cohort study* - summarise follow-up time (*e.g.*, average and total amount)	Page 9 and Table 1<br><br>Page 9 and eFigure 1<br><br>Page 9		
Outcome data	15	*Cohort study* - Report numbers of outcome events or summary measures over time<br>*Case-control study* - Report numbers in each exposure	Page 10<br><br>N/A		

For peer review only

		category, or summary measures of exposure<br>*Cross-sectional study* - Report numbers of outcome events or summary measures	 <br>N/A		
Main results	16	(a) Give unadjusted estimates and, if applicable, confounder-adjusted estimates and their precision (e.g., 95% confidence interval). Make clear which confounders were adjusted for and why they were included<br>(b) Report category boundaries when continuous variables were categorized<br>(c) If relevant, consider translating estimates of relative risk into absolute risk for a meaningful time period	Page 9 and 10<br><br>Tables 3 and 4<br><br>Tables 3 and 4		
Other analyses	17	Report other analyses done—e.g., analyses of subgroups and interactions, and sensitivity analyses	Page 10 and Supplementary Material		
**Discussion**					
Key results	18	Summarise key results with reference to study objectives	Page 11		
Limitations	19	Discuss limitations of the study, taking into account sources of potential bias or imprecision. Discuss both direction and magnitude of any potential bias	Page 13	RECORD 19.1: Discuss the implications of using data that were not created or collected to answer the specific research question(s). Include discussion of misclassification bias, unmeasured confounding, missing data, and changing eligibility over time, as they pertain to the study being reported.	Page 13
Interpretation	20	Give a cautious overall interpretation of results considering objectives,	Pages 11 to 13		

For peer review only - http://bmjopen.bmj.com/site/about/guidelines.xhtml

		limitations, multiplicity of analyses, results from similar studies, and other relevant evidence			
Generalisability	21	Discuss the generalisability (external validity) of the study results	Pages 12 to 13		
**Other Information**					
Funding	22	Give the source of funding and the role of the funders for the present study and, if applicable, for the original study on which the present article is based	Page 2		
Accessibility of protocol, raw data, and programming code		..		RECORD 22.1: Authors should provide information on how to access any supplemental information such as the study protocol, raw data, or programming code.	Page 2

*Reference: Benchimol EI, Smeeth L, Guttmann A, Harron K, Moher D, Petersen I, Sørensen HT, von Elm E, Langan SM, the RECORD Working Committee. The REporting of studies Conducted using Observational Routinely-collected health Data (RECORD) Statement. *PLoS Medicine* 2015; in press.

*Checklist is protected under Creative Commons Attribution (CC BY) license.

For peer review only

BMJ Open

Associations of presenting symptoms and subsequent adverse clinical outcomes in people with unipolar depression: a prospective natural language processing, transdiagnostic, network analysis of electronic health record data

Journal:	*BMJ Open*
Manuscript ID	bmjopen-2021-056541.R1
Article Type:	Original research
Date Submitted by the Author:	30-Jan-2022

Complete List of Authors:	Patel, Rashmi; King's College London Institute of Psychiatry Psychology and Neuroscience, Department of Psychosis Studies; South London and Maudsley NHS Foundation Trust<br>Irving, Jessica; King's College London Institute of Psychiatry Psychology and Neuroscience, Department of Psychosis Studies<br>Brinn, Aimee; King's College London Institute of Psychiatry Psychology and Neuroscience, Department of Psychosis Studies<br>Taylor, Matthew; University of Oxford Department of Psychiatry<br>Shetty, Hitesh; South London and Maudsley NHS Foundation Trust<br>Pritchard, Megan; King's College London Institute of Psychiatry Psychology and Neuroscience, Department of Psychological Medicine; South London and Maudsley NHS Foundation Trust<br>Stewart, Robert; King's College London Institute of Psychiatry Psychology and Neuroscience, Department of Psychological Medicine; South London and Maudsley NHS Foundation Trust<br>Fusar-Poli, Paolo; King's College London Institute of Psychiatry Psychology and Neuroscience, Department of Psychosis Studies; South London and Maudsley NHS Foundation Trust<br>McGuire, Philip; King's College London Institute of Psychiatry Psychology and Neuroscience, Department of Psychosis Studies; South London and Maudsley NHS Foundation Trust

**Primary Subject Heading**:	Mental health
Secondary Subject Heading:	Health informatics
Keywords:	Depression & mood disorders < PSYCHIATRY, Schizophrenia & psychotic disorders < PSYCHIATRY, Health informatics < BIOTECHNOLOGY & BIOINFORMATICS, EPIDEMIOLOGY

I, the Submitting Author has the right to grant and does grant on behalf of all authors of the Work (as defined in the below author licence), an exclusive licence and/or a non-exclusive licence for contributions from authors who are: i) UK Crown employees; ii) where BMJ has agreed a CC-BY licence shall apply, and/or iii) in accordance with the terms applicable for US Federal Government officers or employees acting as part of their official duties; on a worldwide, perpetual, irrevocable, royalty-free basis to BMJ Publishing Group Ltd ("BMJ") its licensees and where the relevant Journal is co-owned by BMJ to the co-owners of the Journal, to publish the Work in this journal and any other BMJ products and to exploit all rights, as set out in our licence.

The Submitting Author accepts and understands that any supply made under these terms is made by BMJ to the Submitting Author unless you are acting as an employee on behalf of your employer or a postgraduate student of an affiliated institution which is paying any applicable article publishing charge ("APC") for Open Access articles. Where the Submitting Author wishes to make the Work available on an Open Access basis (and intends to pay the relevant APC), the terms of reuse of such Open Access shall be governed by a Creative Commons licence – details of these licences and which Creative Commons licence will apply to this Work are set out in our licence referred to above.

Other than as permitted in any relevant BMJ Author's Self Archiving Policies, I confirm this Work has not been accepted for publication elsewhere, is not being considered for publication elsewhere and does not duplicate material already published. I confirm all authors consent to publication of this Work and authorise the granting of this licence.

For peer review only

Associations of presenting symptoms and subsequent adverse clinical outcomes in people with unipolar depression: a prospective natural language processing, transdiagnostic, network analysis of electronic health record data

Rashmi Patel*[1,2], Jessica Irving*[1], Aimee Brinn*[1], Matthew Taylor[3], Hitesh Shetty[2], Megan Pritchard[1,2], Robert Stewart[1,2], Paolo Fusar-Poli[1,2], Philip McGuire[1,2]

*Equal contributions to authorship

1. King's College London (Institute of Psychiatry, Psychology and Neuroscience), London, UK

2. South London and Maudsley NHS Foundation Trust, London, UK

3. University of Oxford (Department of Psychiatry), Oxford, UK

Correspondence to:

Rashmi Patel

King's College London (Institute of Psychiatry, Psychology and Neuroscience), London, UK

E-mail: bmj@rpatel.co.uk

Manuscript word count (excluding references): 5,052

Abstract word count (max 300): 300

Keywords: transdiagnostic, mixed features, depression, bipolar disorder, EHR, NLP

For peer review only - http://bmjopen.bmj.com/site/about/guidelines.xhtml

Competing interests: All authors have completed the ICMJE uniform disclosure form at www.icmje.org/coi_disclosure.pdf and declare: RS has received funding from Janssen, GSK and Takeda outside the submitted work. RP has received funding from Janssen, Induction Healthcare and Holmusk outside the submitted work. The other authors declare no competing interests.

Author contributions: The study was conceived by RP. Data extraction and statistical analysis were performed by JI supervised by RP. Reporting of findings were carried out by RP, JI and AB. All authors (RP, JI, AB, MT, HS, MP, RS, PFP, PM) contributed to study design, manuscript preparation and approved the final version.

Ethics approval: The CRIS data resource received ethical approval as a de-identified dataset for secondary mental health research analyses from Oxfordshire REC C (Ref: 18/SC/0372). The study was approved by the SLaM BRC CRIS oversight committee. Consent is not required to analyse the de-identified dataset for approved research studies. Patients may opt-out of inclusion in the de-identified dataset.

Source of funding: HS, MP, RS and PM have received funding from the National Institute for Health Research (NIHR) Biomedical Research Centre at South London and Maudsley NHS Foundation Trust and King's College London, which also supports the development and maintenance of the BRC Case Register RP has received funding from an NIHR Advanced Fellowship (NIHR301690), a Medical Research Council (MRC) Health Data Research UK Fellowship (MR/S003118/1) and a Starter Grant for Clinical Lecturers (SGL015/1020) supported by the Academy of Medical Sciences, The Wellcome Trust, MRC, British Heart Foundation, Arthritis Research UK, the Royal College of Physicians and Diabetes UK. RS has received funding from a Medical Research Council (MRC) Mental Health Data Pathfinder Award to King's College London, an NIHR Senior Investigator Award and the National Institute for Health Research (NIHR) Applied Research Collaboration South London (NIHR ARC South London) at King's College Hospital NHS Foundation Trust.

Role of funder: The views expressed are those of the authors and not necessarily those of the funders. The funders had no role in the design and conduct of the study; collection, management, analysis, and interpretation of the data; preparation, review, or approval of the manuscript; and decision to submit the manuscript for publication.

Data sharing: The data accessed by CRIS remain within an NHS firewall and governance is provided by a patient-led oversight committee. Access to data is restricted to honorary or substantive employees of the South London and Maudsley NHS Foundation Trust and governed by a local oversight committee who review and approve applications to extract and analyse data for research. Subject to these conditions, data access is encouraged and those interested should contact RS (robert.stewart@kcl.ac.uk), CRIS academic lead.

For peer review only

For peer review only - http://bmjopen.bmj.com/site/about/guidelines.xhtml

Abstract

Objective: To investigate the associations of symptoms of mania and depression with clinical outcomes in people with unipolar depression.

Design: A natural language processing electronic health record study. We used network analysis to determine symptom network structure and multivariable Cox regression to investigate associations with clinical outcomes.

Setting: The South London and Maudsley (SLaM) Clinical Record Interactive Search (CRIS) database.

Participants: All patients presenting with unipolar depression between 1st April 2006 and 31st March 2018.

Exposure:

- (i) Symptoms of mania: Elation; Grandiosity; Flight of ideas; Irritability; Pressured speech.
- (ii) Symptoms of depression: Disturbed mood; Anhedonia; Guilt; Hopelessness; Helplessness; Worthlessness; Tearfulness; Low energy; Reduced appetite; Weight loss.
- (iii) Symptoms of mania or depression (overlapping symptoms): Poor concentration; Insomnia; Disturbed sleep; Agitation; Mood instability.

Main Outcomes:

- (i) Bipolar or psychotic disorder diagnosis.
- (ii) Psychiatric hospital admission.

Results: Out of 19,707 patients, at least one depression, overlapping or mania symptom was present in 18,998 (96.4%), 15,954 (81.0%), and 4,671 (23.7%) patients respectively. 2,772 (14.1%) patients subsequently developed bipolar or psychotic disorder during the follow-up period. The presence of at least one mania (HR: 2.00, 95% CI 1.85 to 2.16), overlapping symptom (HR: 1.71, 95% CI 1.52 to 1.92) or symptom of depression (HR: 1.31, 95% CI 1.07 to 1.61) were associated with significantly increased risk of onset of a bipolar or psychotic disorder. Mania (HR: 1.95, 95% CI 1.77 to 2.15) and overlapping symptoms (HR: 1.76, 95% CI 1.52 to 2.04) were associated with greater risk for psychiatric hospital admission than symptoms of depression (HR: 1.41, 95% CI 1.06 to 1.88).

Conclusions: The presence of mania or overlapping symptoms in people with unipolar depression is associated with worse clinical outcomes. Symptom-based approaches to defining clinical phenotype may facilitate a more personalised treatment approach and better predict subsequent clinical outcomes than psychiatric diagnosis alone.

For peer review only

For peer review only - http://bmjopen.bmj.com/site/about/guidelines.xhtml

Article summary

Strengths and limitations

- By applying natural language processing (NLP) techniques to electronic health records, we were able to obtain symptom data routinely collected by clinicians for a large sample of 19,707 individuals, permitting investigation into associations between specific symptom domains and clinical outcomes.
- NLP enabled us to detect subgroups of patients with unipolar depression also experiencing symptoms of mania (i.e. depression with mixed features) that cannot be discretely identified through ICD-10 diagnostic classification.
- Network analysis allowed us to identify the degree to which certain symptoms co-occur and influence each other as part of the clinical presentation.
- NLP techniques are unable to achieve 100% precision and recall and so the presence of symptoms in electronic health records may not perfectly reflect the true prevalence of symptoms within the cohort.
- Our outcome data derive from a secondary mental healthcare setting and cannot be generalised to people with unipolar depression that are managed within primary care or in the community.

For peer review only

For peer review only - http://bmjopen.bmj.com/site/about/guidelines.xhtml

Introduction

Unipolar depression is one of the most prevalent mental disorders and can lead to impaired functioning and considerable illness burden.[1] A proportion of people with unipolar depression present with concurrent subthreshold manic/hypomanic symptoms known as "mixed features". These can include elevated mood, grandiosity, pressure of speech, flight of ideas, increased energy, increased risk taking and decreased need for sleep. The estimated prevalence of at least one manic symptom and at least three manic systems among people with unipolar depression is 22-50% and 7-23% respectively.[2] Evidence indicates that unipolar depression with mixed features requires only two or three concomitant manic symptoms to be associated with a more severe and disabling course of illness,[3,4] increased relapse frequency,[3,5] increased risk for suicidality,[5] poorer antidepressant response,[6,7] and greater functional difficulties.[4,5] More recently, attenuated manic and cyclothymic features in young people with unipolar depression have been operationalised by the Semistructured Interview for Bipolar At Risk States. This psychometric interview defines subgroup of clinical high-risk states for developing a first episode of bipolar disorder; pilot findings suggest a promising prognostic accuracy.[8] The clustering of symptoms indicated by the 'SIBARS' suggest that aggregation of specific *symptoms* may be useful to detect early phases preceding the onset of bipolar disorder and other severe clinical outcomes. This could theoretically allow early identification of those more likely to develop bipolar disorders and therefore implement preventive approaches that could benefit their outcomes.

Investigating the earlier phases preceding the onset of a bipolar disorder is complicated by the need of prospective designs that represent real world clinical course of this disorder. Electronic health records (EHRs) offer empirical advantages tackling these issues by allowing large scale investigation of pattern of symptoms that may preceded the onset of the disorder. Natural language processing (NLP) is an innovative, automated information extraction technique to investigate the associations of specific symptoms of mental disorders with clinical outcomes using EHR data from mental healthcare.[9–12]

These associations could be further elucidated by adopting network analyses, which investigate the co-occurrence and cross-influence of individual symptoms as part of the clinical presentation. Network analysis has previously demonstrated that the presence of mixed features in people with unipolar depression is associated with poorer response to lurasidone compared to people without mixed features (n=208).[13] A study applying network analysis techniques to mood symptom data obtained through online questionnaires (n=647) and outpatient clinical assessments (n=1,370) demonstrated that symptoms of mania and depression are transdiagnostic and can occur in either unipolar depression or bipolar disorder.[14] However, to the best of our knowledge, there have been no studies which have investigated the relationship between symptoms of mania and depression through network analysis applied to large volumes of EHR data.

We aimed to identify how often symptoms of mania are recorded in the EHR of patients who initially present with unipolar depression, and the association of symptoms of mania with subsequent diagnosis of bipolar or psychotic disorder, and with psychiatric hospital admissions. We represent symptoms of depression and mania graphically using network analysis in a sample of 19,707 patients presenting to a secondary mental healthcare service in South London.

For peer review only - http://bmjopen.bmj.com/site/about/guidelines.xhtml

Methods

Participants and setting

We extracted data from de-identified EHRs for individuals accessing secondary mental health care provided by the South London and Maudsley NHS Foundation Trust (SLaM; London, UK) between 1st April 2006 to 31st March 2018. SLaM holds EHRs for over 500,000 people in receipt of mental healthcare since 2007.[15] The Trust provides community and inpatient mental healthcare within its catchment area covering the London boroughs of Lambeth, Southwark, Croydon and Lewisham. We used the Clinical Record Interactive Search tool (CRIS) to extract and analyse de-identified EHR data from SLaM, with full access to the database population used to create the study population.[15]

Inclusion criterion: all individuals with a primary diagnosis of unipolar depression (eTable 1) recorded in a structured diagnosis EHR field who presented to a SLaM community or inpatient service between 1st April 2006 and 31st March 2018. Follow-up data were obtained from the date of acceptance to the community or inpatient service in which the first recorded diagnosis of unipolar depression was made (the *index date*).

Exclusion criteria: patients with a prior diagnosis of bipolar disorder (ICD-10: F31*), mania (ICD-10: F30*) or psychotic disorder (ICD-10: F1x.5, F2x, F32.3 and F33.3) were excluded. We further excluded patients who went on to receive a diagnosis of psychotic disorder, bipolar disorder or mania within 3 months following their first depression diagnosis. This exclusion criterion was employed to exclude individuals whose first presentation diagnosed as unipolar depression could have represented the initial phase of a bipolar disorder. We excluded patients with a delay of one year or more between the index date and first unipolar depression diagnosis date as the symptoms recorded around the time of presentation may not have represented symptoms associated with the recorded diagnosis of unipolar depression for individuals with a long delay between initial referral and subsequent unipolar depression diagnosis. Patients with no recorded symptoms or missing ethnicity data were also excluded.

Measures

Data were extracted on age at index date, gender, ethnicity, symptoms of depression or mania (including those which could be a feature of either depression or mania), psychiatric hospital admissions (voluntary and involuntary under the UK Mental Health Act) and diagnoses of bipolar disorder (ICD-10: F30/31) or psychotic disorders (ICD-10: F1x.5, F2x, F32.3 and F33.3). Ethnicity was recoded according to categories defined by the UK Office of National Statistics (ONS) as Asian, Black, Mixed, Other and White (eTable 2).

We obtained data for 20 symptoms using NLP tools developed for use on EHR data obtained using CRIS (eTable 3). These symptoms were chosen based on their inclusion in either ICD-10 or DSM-5 diagnostic criteria for unipolar depression and/or mania with the exception of mood instability which is not present in diagnostic criteria for either disorder but was included because it has been previously shown to be an important clinical feature in people with unipolar depression and bipolar disorder.[9] The CRIS NLP tools extract symptom data from unstructured free text clinical documents which record clinical assessment, mental state examination and the agreed treatment plan. Their performance is measured through precision and recall. In this case, precision refers to the number of true positive instances that the NLP application has identified, divided by the total number of

For peer review only - http://bmjopen.bmj.com/site/about/guidelines.xhtml
For peer review only

instances retrieved by the NLP application (both true positive and false positive instances). Recall refers to the number of true positive instances identified by the NLP application, divided by the total number of existing true positives in the entire sample. Precision and recall statistics for the NLP applications employed in this as well as further details on their derivation, are provided in the CRIS Natural Language Processing Applications Library.[16]

Symptom data were drawn from a period within three months either side of the index date, as symptoms presenting in this time are more likely to inform initial presentation. We converted each symptom into dichotomous variables of *present* vs *absent* in a given patient's record. This approach was chosen instead of quantifying the number of times each symptom was recorded in order to minimise confounding by the volume of documentation within a patient's record which may vary depending on illness severity and mode of clinical presentation (e.g. outpatient vs inpatient). We classified symptoms *a priori* into three groups according to ICD-10/DSM-5 diagnostic criteria (see eTable 3): depressive, mania and overlapping symptoms (i.e. symptoms present in ICD-10/DSM-5 classification criteria for both unipolar depression and bipolar disorder).

Follow-up data were obtained from 3 months following the index date for each patient up to 31st March 2021 to ensure predictor symptom data were temporally separate from outcome data. Psychiatric hospitalisation data were obtained as the date of first recorded admission to a SLaM psychiatric hospital during the follow-up period and categorised as (a) any psychiatric hospital admission, and (b) compulsory psychiatric hospital admission under section 2 or 3 of the UK Mental Health Act (MHA). Data on subsequent diagnosis of psychotic disorder or bipolar disorder were obtained from structured diagnostic fields and unstructured free text EHR documents using NLP.[16] We chose to obtain follow-up diagnosis data from both structured and unstructured fields to maximise early identification of onset of these disorders as clinicians typically document emerging diagnoses in unstructured free text prior to recording the diagnosis in the structured EHR fields.

Statistical analysis

We followed the TRANSD ("TRANSD"iagnostic research recommendations in psychiatry) reporting guidelines (eTable 4).[17] All analyses were conducted in R (version 3.6.1). We obtained descriptive statistics on age, gender, ethnicity and the most commonly recorded symptoms and symptom combinations. To investigate demographic associations with recording of manic symptoms among patients diagnosed with unipolar depression, we assessed associations between the presence of at least once manic symptom and at least one overlapping symptom with age, gender and ethnicity through univariate logistic regression.

We used network analysis to visually examine connectivity between depressive and manic symptoms. In a network graph, circles represent nodes (symptom items) and lines represent edges. Each edge denotes a partial correlation between two symptoms (i.e., the correlation between two symptoms when controlling for all other symptoms in the network). Edge thickness represents correlation magnitude. We estimated the network using the Enhanced Least Absolute Shrinkage and Selection Operator (eLASSO) procedure implemented via the *EstimateNetwork* package. The *qgraph* package was used to visualise the network.

Node placement was determined using the Fruchterman-Reingold algorithm, which places the most central nodes visually centre in the network graph.[18] In terms of centrality indices, we calculated *strength, closeness* and *betweenness* defined in eTable 5. We used a case-dropping bootstrapping

For peer review only - http://bmjopen.bmj.com/site/about/guidelines.xhtml

procedure to assess network stability. Further information on our network analysis approach can be found in the supplementary material (eMethods 1).

We created dichotomous predictor variables indicating where individuals had at least one reported symptom within each of the following symptom groups: mania, depression, overlapping. We chose to dichotomise these predictors (i.e. the presence of at least one symptom in each group was recorded as "1" and absence of any symptom within a group recorded as "0") as symptom groups had unequal numbers of constituent symptoms, therefore treating these predictors as continuous would have weighted one symptom group over the other. Additionally, we examined the associations of each individual symptom within the mania and overlapping group with the outcomes described subsequently.

We included three demographic covariates in multivariable analyses to evaluate associations of symptom groups with clinical outcomes: age, gender and ethnicity. Using these covariates and the predictors described above, we estimated Cox proportional hazards regression models (censor date: 31st March 2021) to predict hazard ratios (HR) for risk of the following outcomes:

(i) Receiving a diagnosis of bipolar or psychotic disorder

(ii) Psychiatric hospital admission (any)

(iii) Compulsory psychiatric admission under the UK Mental Health Act (MHA)

(iv) Receiving a diagnosis of bipolar disorder

(v) Receiving a diagnosis of psychotic disorder

Multivariable analyses were corrected for multiple-testing using the Bonferroni method.

Bipolar disorder was defined according to the following ICD-10 diagnostic categories: ICD-10 F30 and F31.

Psychotic disorder was defined according to the following ICD-10 diagnostic categories: ICD-10 F1x.5, F2x, F32.3 and F33.3.

Where a patient was diagnosed with both bipolar disorder and psychotic disorder during the follow-up period, the first recorded diagnosis was considered when assessing outcome (i) listed above.

Patient and public involvement

The study was approved by the SLaM CRIS Oversight Committee which has service user representation. Researchers wishing to access CRIS data can make an application to the Oversight Committee via the CRIS website (https://projects.slam.nhs.uk/research/cris/cris-project-application). The design and completion of approved CRIS projects is supported by a service user advisory group (the CRIS SUCAG) whose members have experience of receiving mental healthcare (or providing care to someone receiving mental healthcare) from SLaM clinical services.[19]

For peer review only - http://bmjopen.bmj.com/site/about/guidelines.xhtml

For peer review only

Results

Descriptive statistics

The final sample comprised 19,707 patients, having excluded 1,206 patients with over a year delay between the index date and unipolar depression diagnosis, 3,407 patients with no documented symptom data, 2,364 patients with missing ethnicity data and a further 7 patients with missing gender data (see eFigure 1).

Demographic and clinical characteristics are reported in Table 1. Our sample was predominantly female (61.9%), of White ethnicity (59.5%), with a mean age (SD) of 38.0 (12.3) years. Recorded symptoms of depression were ascertained in 18,998 patients (96.4%), diagnostically overlapping symptoms in 15,954 patients (81.0%) and symptoms of mania in 4,671 patients (23.7%). Patients with at least one symptom of mania were significantly more likely to be male (OR: 1.15, 95% CI 1.08 to 1.23, $p < 0.001$) and significantly less likely to be of Other ethnicity relative to White ethnicity (OR: 0.85, 95% CI 0.76 to 0.94, $p = 0.006$). Patients with at least one diagnostically overlapping symptom were also significantly more likely to be male (OR: 1.20, 95% CI 1.11 to 1.29, $p < 0.001$), significantly more likely to be of Asian ethnicity relative to White ethnicity (OR: 1.26, 95% CI 1.07 to 1.49, $p = 0.019$).

We extracted a median of 11 (IQR: 5 to 24) symptoms per patient, reported in 6,910 different combinations. There were 642 combinations of symptoms of depression, 32 combinations of overlapping symptoms and 31 combinations of symptoms of mania. We found at least one overlapping symptom recorded for 15,954 patients (81.0%). At least two overlapping symptoms were recorded for 10,119 patients (51.3%) and at least three were recorded for 5,188 patients (26.3%). At least one mania symptom was recorded for 4671 (23.7%) patients. 634 (3.2%) patients had at least two recorded symptoms of mania, whilst three or more symptoms were recorded for 144 (0.73%) patients.

The distribution of ICD-10 unipolar depression diagnoses across the cohort is reported in eTable 6 . The most common index diagnosis was F32.1 Moderate depressive episode (n = 5,242, 26.6%).

Network analysis

Figure 1 presents the regularised partial correlation network of symptoms in people with unipolar depression. Symptoms of mania cluster between themselves, and symptoms of depression and some diagnostically overlapping symptoms cluster together (including within-group clustering). Visual inspection of the network graph revealed two diagnostically overlapping symptoms (agitation and mood instability) with strong associations to the principal symptoms of mania.

The resulting network remained stable after dropping up to 33% of cases from the sample. The strongest edges for each symptom are presented in Table 2. The strongest association was between pressured speech and flight of ideas; the second strongest association was between hopelessness and helplessness. Symptom centrality indices (strength, closeness, betweenness and expected influence) are provided in eFigure 2. Agitation was the most central symptom in terms of strength, betweenness and expected influence. Disturbed mood was the most central symptom in terms of closeness. Stability analysis of the centrality indices suggests correlation stability coefficients of 0.67 for Strength, 0.67 for Betweenness and 0.67 for Closeness.[20]

For peer review only - http://bmjopen.bmj.com/site/about/guidelines.xhtml

For peer review only

Diagnostic outcomes

During 166,686.5 person-years of follow-up (mean: 8.45 years, std dev: 15.0 years), 2,772 (14.07%) individuals received a subsequent diagnosis of a psychotic or bipolar disorder; 2,150 (10.91%) received a psychotic disorder diagnosis and 986 (5.00%) received a bipolar disorder diagnosis. The incidence rate (cases per 10,000 person-years risk) was 166.4 (95% CI: 160.2 to 172.7) for diagnosis of psychotic or bipolar disorder, 128.6 (95% CI: 123.2 to 134.2) for diagnosis of psychotic disorder and 59.0 (95% CI: 55.4 to 62.8) for diagnosis of bipolar disorder.

Results of multivariable Cox proportional hazard analyses, corrected for multiple-testing, are reported in Table 3. In decreasing order of risk, individuals with at least one symptom of mania, at least one overlapping symptom, or at least one symptom of depression had significantly increased risk for bipolar or psychotic disorder. Pressured speech (HR: 3.07, 95% CI 2.56 to 3.69), grandiosity (HR: 3.04, 95% CI: 2.46 to 3.75), and flight of ideas (HR: 2.83, 95% CI: 2.30 to 3.48) were the individual symptoms most strongly associated with subsequent onset of psychotic or bipolar disorder. Black or Asian ethnicity (relative to White ethnicity) and age were also significant predictors of risk of subsequent diagnosis of bipolar or psychotic disorder.

Cox proportion hazard analyses for risk of subsequent diagnosis of bipolar disorder are reported in eTable 7. In a multivariable analysis, individuals with at least one symptom of mania or at least one overlapping symptom had a significantly increased risk of subsequent bipolar disorder diagnosis. Individual symptoms: grandiosity (HR: 4.36, 95% CI 3.24 to 5.87), elation (HR: 4.21, 95% CI 3.46 to 5.12) and pressured speech (HR: 4.07, 95% CI 3.12 to 5.31) were most strongly associated with subsequent onset of bipolar disorder.

Cox proportion hazard analyses for risk of subsequent diagnosis of psychotic disorder are reported in eTable 8. In decreasing order of risk, individuals with at least one mania symptom, at least one overlapping symptom or at least one depression symptom had a significantly increased risk of onset of psychotic disorder. All individual overlapping/mania symptoms were significantly associated with subsequent psychotic disorder diagnosis. Of these individual symptoms, pressured speech (HR: 2.69, 95% CI: 2.17 to 3.33), flight of ideas (HR: 2.61, 95% CI 2.07 to 3.30), and grandiosity (HR: 2.61, 95% CI: 2.03 to 3.36), were most strongly associated with the onset of psychotic disorder.

Psychiatric hospital admission

There were 1,719 psychiatric admissions, of which 516 were compulsory under the UK MHA. Table 4 presents the results from the Cox regression analyses for risk of psychiatric hospital admission and eTable 9 for risk of compulsory psychiatric hospital admission under the UK MHA. Symptoms of depression, symptoms of mania and overlapping symptoms were all associated with non-compulsory hospitalisation risk. Mania and overlapping symptoms predicted compulsory hospitalisation, whilst depressive symptoms did not. Of the individual overlapping/mania symptoms, pressured speech (HR: 3.02, 95% CI: 2.41 to 3.77), agitation (HR: 2.91, 95% CI: 2.65 to 3.20), and flight of ideas (HR: 2.54, 95% CI: 1.98 to 3.25) were associated with the greatest risk of any psychiatric hospital admission.

Pressured speech (HR: 4.37, 95% CI 3.12 to 6.14), elation (HR: 3.96, 95% CI 2.98 to 5.25) and flight of ideas (HR: 3.80, 95% CI 2.61 to 5.53) were associated with the greatest risk of compulsory psychiatric hospital admission. Black and Asian ethnicity were associated with increased risk of compulsory

For peer review only - http://bmjopen.bmj.com/site/about/guidelines.xhtml

psychiatric hospital admission, compared to White ethnicity. Male gender was associated with increased risk of compulsory psychiatric hospital admission, compared to female gender.

For peer review only

For peer review only - http://bmjopen.bmj.com/site/about/guidelines.xhtml

Discussion

We found that people with unipolar depression have a heterogenous clinical phenotype with a significant number (2,772 during 166,686.5 person-years of follow-up) going on to develop a bipolar or psychotic disorder. In a large sample of 19,707 individuals with a diagnosis of unipolar depression, 23.7% had at least one reported symptom of mania (irritability, elation, grandiosity, pressured speech or flight of ideas), and this subset of patients was most likely to be White and male. Irritability was the most commonly documented symptom of mania.

Associations of symptoms with psychotic or bipolar disorder diagnosis

Overall, those with symptoms of mania (i.e. mixed features) had the greatest rates of subsequent diagnosis of a psychotic or bipolar disorder. Symptoms of mania were associated with double the risk of psychotic or bipolar disorder. These results are consistent with previous studies where mixed features or subsyndromal hypomanic symptoms during a depressive episode have been associated with subsequent bipolar disorder diagnosis [21–25]. The current study replicates these previous findings in the largest sample size to date.

Association of symptoms with subsequent hospital admissions

Patients with symptoms of mania were admitted to hospital at the greatest rate, compared to patients with overlapping or depression symptoms. Dudek et al found that patients whose diagnosis evolved from unipolar depression to bipolar disorder experienced more hospitalisations than those who remained with a diagnosis of unipolar depression [23]. Moreover, evidence has suggested that highly recurrent depression (defined as more than 3 episodes) shares some clinical characteristics that are nearer to bipolar disorder [26], such as hypomanic switches while on antidepressant drugs [27] and presence of a family history of bipolar disorder [28]. Those with highly recurrent depression may be expected to be admitted to hospital at greater rates, which may account for the association between mixed features and hospitalisation observed in this study. Overlapping symptoms and depression symptoms were associated with increased risk of subsequent psychiatric hospital admissions. However, symptoms of depression were not associated with increased risk of compulsory psychiatric hospital admission.

The relationship between symptoms of mania and depression

While we investigated a total of five symptoms shared by ICD-10 and DSM-5 criteria for unipolar depression and mania, our network analysis revealed that only agitation and mood instability co-occurred with mania symptoms of irritability, elation, pressured speech, flight of ideas and grandiosity. Symptoms of insomnia, disturbed sleep and poor concentration that are shared between unipolar depression and bipolar disorder were not significantly associated with the principal symptoms of mania. This concurs with the findings of a study that examined the joint structure of symptoms of mania and depression in two large samples totalling over 2,100 patients[14] which found symptoms such as increased energy, grandiosity and euphoria represented a latent specific positive activation dimension that was more specific to bipolar disorder. We found that our identified overlapping symptoms of agitation and mood instability were associated with subsequent psychotic or bipolar disorder diagnosis.

Highly central symptoms are most likely to sustain the rest of the network. Betweenness centrality is one of the most useful metrics for detecting nodes that connect one region of a network analysis graph to another. Removal of these nodes may therefore represent an opportunity to destabilise the

For peer review only - http://bmjopen.bmj.com/site/about/guidelines.xhtml

network. Agitation and mood instability were the two overlapping symptoms that showed the highest betweenness centrality. In theory, changes in nodes with the highest betweenness centrality should have the greatest effect on other nodes, in this case – symptoms of depression and mania. This finding may suggest that clinicians should be especially vigilant in identifying symptoms of agitation or mood instability in patients with unipolar depression, as they could be associated with of subsequent diagnosis of bipolar disorder and/or increased risk of subsequent psychiatric hospitalisation. However in cross-sectional datasets like this one, betweenness and closeness have been found to show low stability and wide confidence intervals .[29]

DSM-5 criteria for unipolar depression with mixed features (eTable 10) require, along with a major depressive episode, the presence of at least three of seven symptoms of mania including elevated mood, grandiosity, pressured speech, flight of ideas, increased energy, risk-taking behaviour and decreased need for sleep. However, this clinical picture is extremely rare in mixed depressive states.[2] Furthermore, the DSM-5 criteria for a diagnosis of unipolar depression with mixed features does not account for psychomotor agitation, irritability and mood instability, which Koukopoulos and Sani describe as 'core features' of this clinical presentation.[2] This concurs with our network analysis which finds symptoms of agitation and mood instability co-occur more commonly with symptoms of mania than other overlapping symptoms such as disturbed sleep and poor appetite. Therefore, the DSM-5 criteria for unipolar depression with mixed features exclude patients at risk of substantial functional impairment who may need enhanced support and more tailored invention.[2] Certain treatments such as SSRIs could prove inappropriate for these patients by worsening agitation and increasing the risk of onset of mania[30] and hospitalisation.[2]

Demographic factors also contributed to risk for poor outcomes. Patients of Black and Asian ethnicity were almost three times and two times more likely respectively than White patients to be compulsorily admitted to a psychiatric hospital under the UK MHA. Previous studies have shown that Black and Minority Ethnic people are disproportionately likely to be admitted to a psychiatric hospital under the UK MHA.[31]

Strengths

Through our use of EHRs we were able to identify a sample of 19,707 individuals at a scale that would have been infeasible to curate through direct patient recruitment. Furthermore, while the diagnosis of *depressive disorder with mixed features* was introduced in the DSM-5 to capture the phenomenon of depressed patients who exhibit concurrent symptoms of mania or hypomania[3], no analogous diagnosis exists in the ICD-10.

Our approach used automated NLP tools to identify symptoms documented in the EHR allowing us to identify patients for further study. Here, we identified 4,888 patients diagnosed with unipolar depression with symptoms of mania reported in their EHRs. We were able to stratify these patients into a separate group and represent the heterogeneity of unipolar depression. As a result, we were able to identify differences in clinical outcomes between patients diagnosed with the same heterogeneous diagnostic construct.

Our approach using EHRs provides sample sizes permissive of more granular analyses into specific symptom domains than in previous studies. The use of NLP allowed us to obtain symptom data that tend to be unrepresented in large scale administrative resources. The symptom NLP algorithms allow more fine-grained definition of item constructs than that of typical depression criteria. Mania symptoms are not routinely elicited in standard depression screening questionnaires, and

For peer review only - http://bmjopen.bmj.com/site/about/guidelines.xhtml

classification systems sometimes combine opposing poles of disorder into unitary terms (like agitation/psychomotor retardation) when they are likely to reflect different underlying neurobiological processes.[32] Our approach allowed us to extract data on agitation and psychomotor retardation separately.

We followed the TRANSD guidelines for reporting transdiagnostic research in psychiatry.[17] We provided a transparent definition of specific ICD-10 diagnostic codes which were fully detailed and numerated to characterise the cohort of patients with unipolar depression and the outcomes of bipolar or psychotic disorders ("T" and "N" criteria). We reported the primary outcome and the study design and defined the transdiagnostic construct as symptoms overlapping unipolar depression and bipolar disorder and reported the primary outcome of the study (receiving a subsequent bipolar or psychotic disorder diagnosis) and study design (prospective natural language processing EHR study) fulfilling the "R" criterion. We appraised the conceptual framework as across-at least three diagnoses (unipolar depression, bipolar disorders, psychosis) and across two diagnostic spectra (mood disorders and psychotic disorders) fulfilling the "A" criterion.

The ability to identify subgroups of people with unipolar depression and symptoms of mania (as mixed features) would enable the development and evaluation of interventions specifically designed to treat mixed features such as agitation and mood instability which have a significant impact on outcomes and may not be well treated by traditional antidepressant or psychological therapy. For example, patients with symptoms of elevated mood and agitation were less likely than patients without these symptoms to benefit from lurasidone in terms of their MADRS and CGI-S Scores.[13] Conversely, presence of rapid/pressured speech predicted improvement in both mania and depression symptoms with lurasidone. These opposing effects would have been missed in standard approaches that group patients by diagnostic category, thereby treating all symptoms as equally important indicators of prognosis.

Limitations

NLP techniques are unable to achieve 100% precision and recall and therefore classify information imperfectly.[33] Therefore the prevalence of symptoms in this cohort cannot be taken as the true prevalence of symptoms within the cohort. However, the overall symptom network is unlikely to be affected by incorrect NLP classification as the network was stable even when dropping over 6,700 cases (33% of our sample). As we were limited by the availability of existing algorithms, we were unable to obtain symptom data available for some depression and mania criteria, for example increased appetite or hypersomnia (depression), or impulsivity and hypersexuality (mania).

We cannot infer temporal relations between symptoms as our symptom data derive from one timepoint only. Some nodes measure overlapping constructs such an insomnia and sleep disturbances; these variables were however not strongly correlated, suggesting that clinicians are using these terms to report separate, but related, phenomena. As we limited our study to people presenting with unipolar depression, we were unable to assess the degree to which a transdiagnostic approach using NLP-derived symptom data could be better or worse at predicting future clinical outcomes compared to assessing initial ICD-10 diagnoses (TRANSD criterion "S").[12] Our data derive from a secondary mental healthcare setting and cannot be generalised to cases of unipolar depression that are managed exclusively within primary care. Future external validation studies should be performed as recommended by the TRANSD reporting guidelines (TRANSD criterion "D").[17]

For peer review only - http://bmjopen.bmj.com/site/about/guidelines.xhtml

This study is also limited by the provenance of our data which restricts access to important clinical covariates. As our data is derived from a secondary mental healthcare case register, we do not have access to primary healthcare records. We therefore are unable to include comprehensive data on medications for people treated in primary care prior to referral to secondary care. Medication data is an important covariate because they can cause certain side effects such as agitation, and can increase risk of mania in some patients. [30,34,35]

Finally, we were also unable to extract data on substance use, symptoms of anxiety, and the setting in which patients received their diagnosis (inpatient vs community). Substance use is a predictor of bipolar disorder [36], whilst 'mixed anxiety and depressive disorder' is a common unipolar depression diagnosis, so they are important covariates to be included in future studies. Diagnosis setting may be a significant covariate when predicting rates of hospitalisation; patients who receive their diagnosis in inpatient settings may be more unwell and therefore hospitalised at greater rates. The majority of our sample is likely to have received their diagnosis in primary care settings.

Conclusions

Our findings suggest that clinicians should screen for mania and diagnostically overlapping symptoms in patients who present with unipolar depression as these could be associated with increased risk of subsequently developing a psychotic or bipolar disorder and be associated with increased likelihood of psychiatric hospital admission.[12] The DSM-5 criteria for depression with mixed features does not include the symptoms 'agitation', 'irritability', or 'mood lability'. [2] Our study suggests patients diagnosed with MDD who present with these symptoms which 'overlap' both clinical depression and mania diagnoses may be at greater risk of developing a psychotic or bipolar disorder or at greater risk of psychiatric hospital admission. Such patients may require enhanced support and more tailored interventions to reduce risk of psychiatric admission. Symptom-level approaches to defining clinical phenotype may enable a better understanding of pathophysiology at the individual patient level, facilitate a more personalised treatment approach, and better predict subsequent clinical outcomes.[37]

Future studies could evaluate whether the presence of mixed features, specifically mood instability and agitation, predict risk of onset of mania when treated with antidepressants.It is important to identify early warning signs for bipolar disorder as treatment approaches for bipolar disorder and unipolar depression differ considerably, and antidepressants can increase risk of mania.[30,34,35]

For peer review only - http://bmjopen.bmj.com/site/about/guidelines.xhtml

References

1 Patel V, Chisholm D, Parikh R, *et al.* Addressing the burden of mental, neurological, and substance use disorders: key messages from Disease Control Priorities, 3rd edition. *Lancet (London, England)* 2016;**387**:1672–85. doi:10.1016/S0140-6736(15)00390-6

2 Koukopoulos A, Sani G. DSM-5 criteria for depression with mixed features: A farewell to mixed depression. *Acta Psychiatr Scand* Published Online First: 2014. doi:10.1111/acps.12140

3 McIntyre RS, Soczynska JK, Cha DS, *et al.* The prevalence and illness characteristics of DSM-5-defined 'mixed feature specifier' in adults with major depressive disorder and bipolar disorder: Results from the International Mood Disorders Collaborative Project. *J Affect Disord* 2015;**172**:259–64. doi:10.1016/j.jad.2014.09.026

4 Nusslock R, Frank E. Subthreshold bipolarity: diagnostic issues and challenges. *Bipolar Disord* 2011;**13**:587–603. doi:10.1111/j.1399-5618.2011.00957.x

5 Angst J, Cui L, Swendsen J, *et al.* Major depressive disorder with subthreshold bipolarity in the national comorbidity survey replication. *Am J Psychiatry* 2010;**167**:1194–201. doi:10.1176/appi.ajp.2010.09071011

6 Rosenblat JD, Mcintyre RS. Treatment recommendations for DSM-5-defined mixed features. CNS Spectr. 2017;**22**:147–54. doi:10.1017/S1092852916000432

7 Suppes T, Ostacher M. Mixed features in major depressive disorder: Diagnoses and treatments. CNS Spectr. 2017;**22**:155–60. doi:10.1017/S1092852917000256

8 Fusar-Poli P, De Micheli A, Rocchetti M, *et al.* Semistructured Interview for Bipolar At Risk States (SIBARS). *Psychiatry Res* 2018;**264**:302–9. doi:https://doi.org/10.1016/j.psychres.2018.03.074

9 Patel R, Lloyd T, Jackson R, *et al.* Mood instability is a common feature of mental health disorders and is associated with poor clinical outcomes. *BMJ Open* 2015;**5**. doi:10.1136/bmjopen-2014-007504

10 Patel R, Jayatilleke N, Broadbent M, *et al.* Negative symptoms in schizophrenia: a study in a large clinical sample of patients using a novel automated method. *BMJ Open* 2015;**5**. doi:10.1136/bmjopen-2015-007619

11 Jackson RG, Patel R, Jayatilleke N, *et al.* Natural language processing to extract symptoms of severe mental illness from clinical text: the Clinical Record Interactive Search Comprehensive Data Extraction (CRIS-CODE) project. *BMJ Open* 2017;**7**.

12 Irving J, Patel R, Oliver D, *et al.* Using Natural Language Processing on Electronic Health Records to Enhance Detection and Prediction of Psychosis Risk. *Schizophr Bull* Published Online First: October 2020. doi:10.1093/schbul/sbaa126

13 Goldberg JF, Siu C, Mao Y, *et al.* Major depressive disorder with mixed features and treatment response to lurasidone: A symptom network model. *J Affect Disord* 2020;**277**:1045–54. doi:10.1016/j.jad.2020.08.048

14 Stanton K, Khoo S, Watson D, *et al.* Unique and Transdiagnostic Symptoms of Hypomania/Mania and Unipolar Depression. *Clin Psychol Sci* 2019;**7**:471–87. doi:10.1177/2167702618812725

15 Perera G, Broadbent M, Callard F, *et al.* Cohort profile of the South London and Maudsley NHS Foundation Trust Biomedical Research Centre (SLaM BRC) Case Register: current status and recent enhancement of an Electronic Mental Health Record-derived data resource. *BMJ Open* 2016;**6**:e008721. doi:10.1136/bmjopen-2015-008721

For peer review only - http://bmjopen.bmj.com/site/about/guidelines.xhtml

16 CRIS Natural Language Processing Applications Library.

17 Fusar-Poli P, Solmi M, Brondino N, *et al.* Transdiagnostic psychiatry: a systematic review. *World Psychiatry* 2019;**18**:192–207. doi:https://doi.org/10.1002/wps.20631

18 Fruchterman TMJ, Reingold EM. Graph drawing by force-directed placement. *Softw Pract Exp* Published Online First: 1991. doi:10.1002/spe.4380211102

19 Jewell A, Pritchard M, Barrett K, *et al.* The maudsley biomedical research centre (Brc) data linkage service user and carer advisory group: Creating and sustaining a successful patient and public involvement group to guide research in a complex area. *Res Involv Engagem* 2019;**5**:20. doi:10.1186/s40900-019-0152-4

20 Epskamp S, Borsboom D, Fried EI. Estimating psychological networks and their accuracy: A tutorial paper. *Behav Res Methods* 2018;**50**:195–212. doi:10.3758/s13428-017-0862-1

21 Mazzarini L, Kotzalidis GD, Piacentino D, *et al.* Is recurrence in major depressive disorder related to bipolarity and mixed features? Results from the BRIDGE-II-Mix study. *J Affect Disord* 2018;**229**:164–70. doi:10.1016/J.JAD.2017.12.062

22 Zimmermann P, Brückl T, Nocon A, *et al.* Heterogeneity of DSM-IV Major Depressive Disorder as a Consequence of Subthreshold Bipolarity. *Arch Gen Psychiatry* 2009;**66**:1341–52. doi:10.1001/ARCHGENPSYCHIATRY.2009.158

23 Rybakowski J, Dudek D, Rybakowski JK, *et al.* Risk factors of treatment resistance in major depression: Association with bipolarity. Published Online First: 2010. doi:10.1016/j.jad.2010.03.001

24 Fiedorowicz JG, Endicott J, Leon AC, *et al.* Subthreshold Hypomanic Symptoms in Progression From Unipolar Major Depression to Bipolar Disorder -- Fiedorowicz et al., 10.1176/appi.ajp.2010.10030328 -- Am J Psychiatry. *Am J Psychiatry* 2011;**168**:40–8.http://ajp.psychiatryonline.org/cgi/content/short/appi.ajp.2010.10030328v1?rss=1

25 Faedda GL, Marangoni C, Serra G, *et al.* Precursors of Bipolar Disorders: A Systematic Literature Review of Prospective Studies. *J Clin Psychiatry* 2015;**76**:14973. doi:10.4088/JCP.13R08900

26 Benazzi F. Highly recurrent unipolar may be related to bipolar II. *Compr Psychiatry* 2002;**43**:263–8. doi:10.1053/comp.2002.33496

27 Kupfer DJ, Carpenter LL, Frank E. Possible role of antidepressants in precipitating mania and hypomania in recurrent depression. *https://doi.org/101176/ajp1457804* 2006;**145**:804–8. doi:10.1176/AJP.145.7.804

28 Akiskal HS, Benazzi F. The DSM-IV and ICD-10 categories of recurrent [major] depressive and bipolar II disorders: Evidence that they lie on a dimensional spectrum. *J Affect Disord* 2006;**92**:45–54. doi:10.1016/j.jad.2005.12.035

29 Epskamp S, Kruis J, Marsman M. Estimating psychopathological networks: Be careful what you wish for. *PLoS One* 2017;**12**:e0179891. doi:10.1371/journal.pone.0179891

30 Patel R, Reiss P, Shetty H, *et al.* Do antidepressants increase the risk of mania and bipolar disorder in people with depression? A retrospective electronic case register cohort study. *BMJ Open* 2015;**5**. doi:10.1136/bmjopen-2015-008341

31 Singh SP, Burns T, Tyrer P, *et al.* Ethnicity as a predictor of detention under the Mental Health Act. *Psychol Med* 2014;**44**:997–1004. doi:10.1017/S003329171300086X

32 Fried EI, Nesse RM. Depression sum-scores don't add up: Why analyzing specific depression symptoms is essential. *BMC Med* 2015;**13**:72. doi:10.1186/s12916-015-0325-4

For peer review only - http://bmjopen.bmj.com/site/about/guidelines.xhtml

33 Maynard D, Bontcheva K. Natural language processing. In: *Perspectives on Ontology Learning*. 2014. doi:10.4018/ijssoe.2014010105

34 Baldessarini RJ, Faedda GL, Offidani E, *et al.* Antidepressant-associated mood-switching and transition from unipolar major depression to bipolar disorder: A review. *J Affect Disord* 2013;**148**:129–35.

35 Geddes JR, Miklowitz DJ. Treatment of bipolar disorder. Lancet. 2013;**381**:1672–82. doi:10.1016/S0140-6736(13)60857-0

36 Corponi F, Fabbri C, Bitter I, *et al.* Novel antipsychotics specificity profile: A clinically oriented review of lurasidone, brexpiprazole, cariprazine and lumateperone. *Eur Neuropsychopharmacol* 2019;**29**:971–85. doi:10.1016/j.euroneuro.2019.06.008

37 Insel TR, Cuthbert BN, Garvey MA, *et al.* Research domain criteria (RDoC): toward a new classification framework for research on mental disorders. *Am J Psychiatry* 2010;**167**:748–51.

For peer review only

For peer review only - http://bmjopen.bmj.com/site/about/guidelines.xhtml

Table 1: Demographic and clinical characteristics

Characteristic	Value
**Demographics**	
Age (Mean, SD)	38.0 (12.3) years
Female (n, (%))	12,196 (61.5%)
Ethnicity (n, (%))	
Asian	1,133 (5.7%)
Black	3,473 (17.6%)
Mixed	439 (2.2%)
Other	2,494 (12.6%)
White	12,168 (61.7%)
**Depressive symptoms (n (%))**	
Disturbed mood	17,188 (87.2%)
Tearfulness	10,196 (51.7%)
Reduced appetite	7,996 (40.6%)
Hopelessness	6,160 (31.3%)
Low energy	6,159 (31.3%)
Guilt	5,828 (29.6%)
Weight loss	3,778 (19.2%)
Anhedonia	3,514 (17.8%)
Worthlessness	2,560 (13.0%)
Helplessness	2,168 (11.0%)
**Diagnostically overlapping symptoms[1] (n (%))**	
Disturbed sleep	12,271 (62.3%)
Poor concentration	8,056 (40.9%)
Mood instability	5,315 (27.0%)
Agitation	5,074 (25.7%)
Insomnia	3,250 (16.5%)
**Manic symptoms (n (%))**	
Irritability	3,911 (19.8%)
Elation	642 (3.3%)
Pressured speech	348 (1.8%)
Flight of ideas	356 (1.8%)
Grandiosity	261 (1.3%)

For peer review only - http://bmjopen.bmj.com/site/about/guidelines.xhtml

[1] Symptoms that overlap ICD-10/DSM-5 criteria for both unipolar and bipolar depression or mania.

Table 2: Strongest associations for each symptom in decreasing order of strength

Node 1	Node 2	Mean edge weight (95% CI)
Pressured speech	Flight of ideas	1.76 (1.35 to 2.15)
Hopelessness	Helplessness	1.65 (1.52 to 1.75)
Elation	Grandiosity	1.50 (1.11 to 1.87)
Poor appetite	Weight loss	1.11 (0.99 to 1.17)
Grandiosity	Flight of ideas	1.06 (0.53 to 1.68)
Disturbed sleep	Poor appetite	1.08 (1.00 to 1.15)
Flight of ideas	Agitation	0.97 (0.72 to 1.26)
Irritability	Agitation	0.80 (0.72 to 0.89)
Mood instability	Disturbed mood	0.86 (0.74 to 1.01)
Guilt	Worthlessness	0.67 (0.58 to 0.77)
Low energy	Disturbed mood	0.62 (0.49 to 0.75)
Anhedonia	Disturbed mood	0.61 (0.41 to 0.80)
Concentration	Energy	0.57 (0.49 to 0.63)
Insomnia	Anhedonia	0.50 (0.39 to 0.60)
Helplessness	Worthlessness	0.53 (0.41 to 0.65)
Agitation	Disturbed sleep	0.43 (0.35 to 0.52)
Tearfulness	Disturbed mood	0.37 (0.26 to 0.46)
Worthlessness	Disturbed mood	0.31 (0.12 to 0.54)
Weightloss	Disturbed mood	0.02 (-0.09 to 0.09)

For peer review only

For peer review only - http://bmjopen.bmj.com/site/about/guidelines.xhtml

Table 3: Multivariable Cox regression models examining factors associated with subsequent psychotic or bipolar disorder diagnosis

Predictor	Risk of developing psychotic or bipolar disorder (number of events = 2,772)			
	Univariate Hazard Ratio (95% CI)	p value	[a]Multivariable Hazard Ratio (95% CI)	p value
**Demographics**				
Age	1.00 (1.00 to 1.01)	0.209	1.00 (1.00 to 1.01)	0.025
Male gender	1.01 (0.93 to 1.09)	0.843	1.03 (0.95 to 1.11)	0.489
Ethnicity				
White	*Ref*	*Ref*	*Ref*	*Ref*
Asian	1.42 (1.22 to 1.64)	<0.001***	1.44 (1.24 to 1.67)	<0.001***
Black	1.52 (1.39 to 1.67)	<0.001***	1.55 (1.41 to 1.70)	<0.001***
Mixed	1.07 (0.83 to 1.39)	0.644	1.10 (0.85 to 1.43)	0.495
Other	1.03 (0.90 to 1.17)	0.692	1.04 (0.91 to 1.18)	0.561
**Symptom groups**				
Mania (≥ 1 symptom)	1.98 (1.84 to 2.15)	<0.001***	2.00 (1.85 to 2.16)	<0.001***
Overlapping (≥ 1 symptom)	1.73 (1.54 to 1.94)	<0.001***	1.71 (1.52 to 1.92)	<0.001***
Depression (≥ 1 symptom)	1.31 (1.07 to 1.60)	0.012*	1.31 (1.07 to 1.61)	0.010*
**Individual symptoms**				
Irritability	1.85 (1.70 to 2.00)	<0.001***	1.85 (1.71 to 2.01)	<0.001***
Elation	2.55 (2.21 to 2.95)	<0.001***	2.65 (2.29 to 3.07)	<0.001***
Pressured speech	3.05 (2.54 to 3.66)	<0.001***	3.07 (2.56 to 3.69)	<0.001***
Flight of ideas	2.83 (2.31 to 3.48)	<0.001***	2.83 (2.30 to 3.48)	<0.001***
Grandiosity	2.99 (2.42 to 3.69)	<0.001***	3.04 (2.46 to 3.75)	<0.001***
Disturbed sleep	1.52 (1.40 to 1.65)	<0.001***	1.49 (1.37 to 1.62)	<0.001***
Poor concentration	1.50 (1.39 to 1.61)	<0.001***	1.48 (1.38 to 1.60)	<0.001***
Mood instability	1.74 (1.61 to 1.88)	<0.001***	1.79 (1.66 to 1.93)	<0.001***
Agitation	2.27 (2.11 to 2.45)	<0.001***	2.29 (2.12 to 2.47)	<0.001***
Insomnia	1.47 (1.35 to 1.61)	<0.001***	1.48 (1.35 to 1.62)	<0.001***

[a]Multivariable analysis adjusted for age, gender and ethnicity.

For peer review only - http://bmjopen.bmj.com/site/about/guidelines.xhtml

* p < 0.05, ** p < 0.01, *** p <0.001

Table 4: Multivariable Cox proportional hazards regression examining factors associated with psychiatric hospital admission

Predictor	Risk of psychiatric hospital admission (number of events = 1,719)			
	Univariate Hazard Ratio (95% CI)	p value	[a]Multivariable Hazard Ratio (95% CI)	p value
**Demographics**				
Age	1.00 (1.00 to 1.00)	0.666	1.00 (1.00 to 1.00)	0.902
Male gender	1.28 (1.17 to 1.41)	<0.001***	1.32 (1.20 to 1.46)	<0.001***
Ethnicity				
White	*Ref*		*Ref*	*Ref*
Asian	0.98 (0.80 to 1.21)	0.884	0.99 (0.80 to 1.22)	0.902
Black	1.25 (1.11 to 1.40)	<0.001***	1.30 (1.15 to 1.46)	<0.001***
Mixed	1.02 (0.74 to 1.41)	0.884	1.06 (0.77 to 1.46)	0.769
Other	0.58 (0.48 to 0.69)	<0.001***	0.63 (0.52 to 0.75)	<0.001***
**Symptom groups**				
Mania (≥ 1 symptom)	1.97 (1.79 to 2.18)	<0.001***	1.95 (1.77 to 2.15)	<0.001***
Overlapping (≥ 1 symptom)	1.79 (1.55 to 2.07)	<0.001***	1.76 (1.52 to 2.04)	<0.001***
Depression (≥ 1 symptom)	1.38 (1.03 to 1.83)	0.033*	1.41 (1.06 to 1.88)	0.021*
**Individual symptoms**				
Irritability	1.92 (1.73 to 2.13)	<0.001***	1.90 (1.71 to 2.10)	<0.001***
Elation	2.51 (2.09 to 3.02)	<0.001***	2.56 (2.13 to 3.07)	<0.001***
Pressured speech	3.01 (2.40 to 3.76)	<0.001***	3.02 (2.41 to 3.77)	<0.001***
Flight of ideas	2.61 (2.04 to 3.35)	<0.001***	2.54 (1.98 to 3.25)	<0.001***
Grandiosity	2.73 (2.10 to 3.57)	<0.001***	2.58 (1.98 to 3.36)	<0.001***
Disturbed sleep	1.72 (1.55 to 1.92)	<0.001***	1.69 (1.52 to 1.88)	<0.001***
Poor concentration	1.55 (1.41 to 1.71)	<0.001***	1.54 (1.40 to 1.69)	<0.001***
Mood instability	1.86 (1.68 to 2.04)	<0.001***	1.88 (1.71 to 2.08)	<0.001***
Agitation	2.99 (2.72 to 3.29)	<0.001***	2.91 (2.65 to 3.20)	<0.001***
Insomnia	1.69 (1.52 to 1.89)	<0.001***	1.68 (1.51 to 1.88)	<0.001***

For peer review only

For peer review only - http://bmjopen.bmj.com/site/about/guidelines.xhtml

[a]Multivariable analysis adjusted for age, gender and ethnicity.

* $p < 0.05$, ** $p < 0.01$, *** $p < 0.001$

For peer review only

For peer review only - http://bmjopen.bmj.com/site/about/guidelines.xhtml

Figure legends

Figure 1

Circles represent nodes (symptom items) and lines represent edges. Each edge denotes a partial correlation between two symptoms (i.e., the correlation between two symptoms when controlling for all other symptoms in the network); edge thickness represents correlation magnitude. We used the Fruchterman-Reingold algorithm, which places the most central nodes visually centre in the network graph, to determine node placement.[18]

For peer review only

For peer review only - http://bmjopen.bmj.com/site/about/guidelines.xhtml

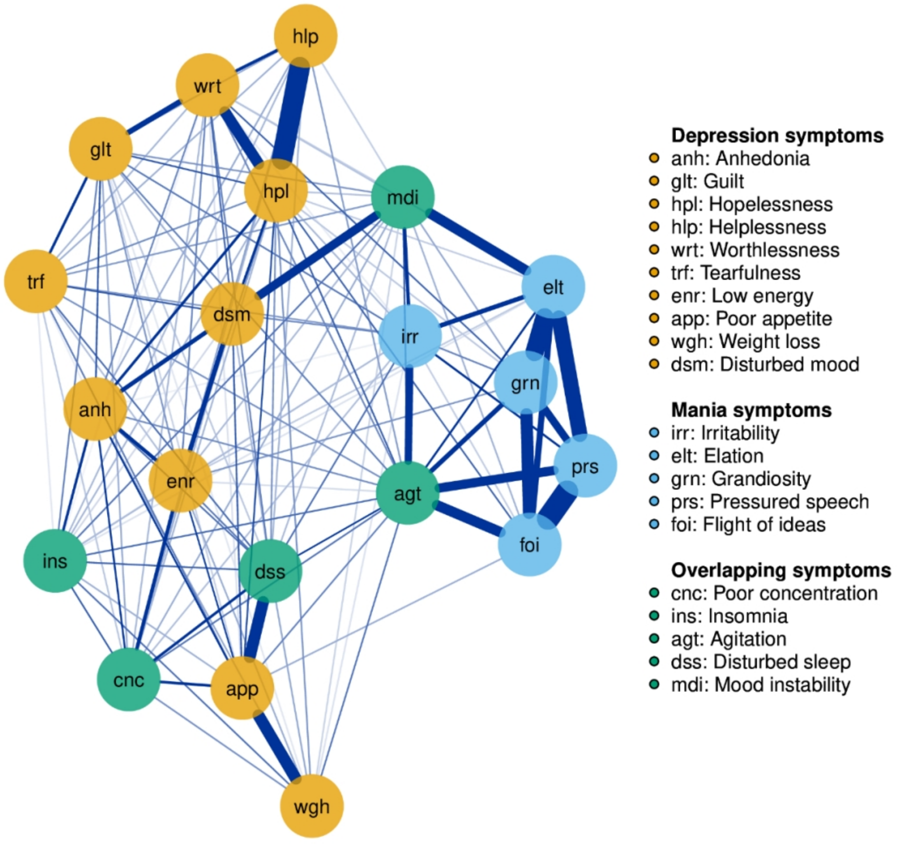

Circles represent nodes (symptom items) and lines represent edges. Each edge denotes a partial correlation between two symptoms (i.e., the correlation between two symptoms when controlling for all other symptoms in the network); edge thickness represents correlation magnitude. We used the Fruchterman-Reingold algorithm, which places the most central nodes visually centre in the network graph, to determine node placement.[18]

159x159mm (220 x 220 DPI)

SUPPLEMENTARY MATERIAL

Associations of presenting symptoms and subsequent bipolar/psychotic disorder onset in people with unipolar depression: a prospective natural language processing, transdiagnostic, network analysis of electronic health record data

Table of Contents

Table of Contents 1

eMethods 1: Network analysis 2

eTable 1: Diagnosis inclusion criteria 2

eTable 2: Ethnicity classification 3

eTable 3: Categorisation of symptoms 4

eTable 4: "TRANSd"iagnostic research recommendations 5

eTable 5: Centrality definitions[2] 6

eTable 6: Frequency of recorded ICD-10 unipolar depression diagnoses 7

eTable 7: Multivaribale Cox regression models examining factors associated with subsequent bipolar disorder diagnosis 8

eTable 8: Multivariable Cox regression models examining factors associated with subsequent psychotic disorder diagnosis 9

eTable 9: Multivariable Cox proportional hazards regression examining factors associated with compulsory psychiatric hospital admission 10

eTable 10: DSM-5 mixed features specifier of depression[3] 11

eFigure 1: Cohort definition and missing data 12

eFigure 2: Centrality indices for overall network 13

References 14

For peer review only - http://bmjopen.bmj.com/site/about/guidelines.xhtml

eMethods 1: Network analysis

The Enhanced Least Absolute Shrinkage and Selection Operator (eLASSO) procedure implemented via the *EstimateNetwork* package is based on the Ising model which is indicated for analysis of binary data. The method combines L1-regularised logistic regression with model selection using a tuning parameter selected by minimizing the Extended Bayesian Information Criterion (EBIC) to achieve sparsity. Sparse symptom networks have much fewer links between symptoms than the possible maximum number of links within that network (where every symptom would be connected). The use of LASSO regularization creates a more parsimonious network model that returns only the most prominent associations between symptoms, and excludes spurious associations. This in turn maximises model interpretability.[1]

In terms of centrality indices, we calculated *strength, closeness* and *betweenness* defined in eTable 4. Centrality indices measure how likely a symptom is to mediate associations between other symptoms or be strongly connected to other symptoms, regardless of that symptom's prevalence. In brief, nodes of high *strength* are likely to be associated with most members of the network, low *closeness* centrality indicates that a node is closely connected with most other nodes in the network (less likely to be peripheral) and *betweenness* centrality indicates the amount of influence a node has over the flow of information in a graph.[2]

We used a case-dropping bootstrapping procedure to assess network stability. In this procedure, the network is reconstructed, and symptom centrality indices are recalculated over multiple iterations in which cases are dropped in increasing proportions. This generates a Correlation Stability Coefficient, which indicates the maximum proportion of cases that can be dropped from the sample without causing the correlation between the original and bootstrapped centrality values to drop below r = 0.7. The network is deemed to be stable if the correlation stability coefficient survives above 0.5.[1]

eTable 1: Diagnosis inclusion criteria

ICD-10 code	Diagnosis
F32 – Depressive episode	F32.9 - Depressive episode, unspecified<br>F32.2 - Severe depressive episode without psychotic symptoms<br>F32.0 - Mild depressive episode<br>F32.1 - Moderate depressive episode<br>F32.8 - Other depressive episodes
F33 – Recurrent depressive disorder	F33.1 - Recurrent depressive disorder, current episode moderate<br>F33.4 - Recurrent depressive disorder, currently in remission<br>F33.0 - Recurrent depressive disorder, current episode mild<br>F33.8 - Other recurrent depressive disorders<br>F33.9 - Recurrent depressive disorder, unspecified
F41 – Anxiety disorder	F41.2 - Mixed anxiety and depressive disorder

Note: All diagnoses were drawn from the structured field Primary Diagnosis *in EHR*

For peer review only - http://bmjopen.bmj.com/site/about/guidelines.xhtml

eTable 2: Ethnicity classification

**Ethnic group**	**Ethnicity as recorded in patient electronic health records**
Black	Black or Black British - African
	Black or Black British - Caribbean
	Black or Black British - Any other Black background
White	White - British
	White - Irish
	White - Any other White background
Asian	Asian or Asian British - Bangladeshi
	Asian or Asian British - Indian
	Asian or Asian British - Pakistani
	Asian or Asian British - Any other Asian background
	Other Ethnic Groups – Chinese
Mixed	Mixed - White and Asian
	Mixed - White and Black African
	Mixed - White and Black Caribbean
	Mixed - Any other mixed background
Other	Other Ethnic Groups - Any other ethnic group
Missing	Not Stated
	Not Recorded

For peer review only

For peer review only - http://bmjopen.bmj.com/site/about/guidelines.xhtml

eTable 3: Categorisation of symptoms

Depressive symptoms	Overlapping symptoms	Mania symptoms
• Disturbed mood	• Poor concentration	• Irritability
• Anhedonia	• Insomnia	• Elation
• Guilt	• Agitation	• Grandiosity
• Weight loss	• Disturbed sleep	• Flight of ideas
• Reduced appetite	• Mood instability	• Pressured speech
• Hopelessness		
• Helplessness		
• Worthlessness		
• Tearfulness		
• Low energy		

For peer review only

For peer review only - http://bmjopen.bmj.com/site/about/guidelines.xhtml

eTable 4: "TRANSd"iagnostic research recommendations

Transparent definition of the gold standard (ICD, DSM, other), including specific diagnostic types, official codes, primary vs. secondary diagnoses, diagnostic assessment interviews.

Report the primary outcome of the study, the study design and the definition of the transdiagnostic construct in the abstract and main text.

Appraise the conceptual framework/approach of the transdiagnostic approach: across-diagnoses, beyond-diagnoses, other (explain).

Numerate the diagnostic categories, spectra and non-clinical samples in which the transdiagnostic construct is being tested and then validated.

Show the degree of improvement of the transdiagnostic approach against the specific diagnostic approach through specific comparative analyses.

Demonstrate the generalizability of the transdiagnostic construct through external validation studies.

Fusar-Poli P, Solmi M, Brondino N, et al. Transdiagnostic psychiatry: a systematic review. World Psychiatry 2019;18:192–207. doi:https://doi.org/10.1002/wps.20631

For peer review only - http://bmjopen.bmj.com/site/about/guidelines.xhtml

eTable 5: Centrality definitions[2]

Centrality	Calculation	Interpretation
**Betweenness**	The percentage of shortest paths that must go through that specific node.	Describes the amount of influence a node has over the flow of information in a graph. Useful to find nodes that bridge from one part of a graph to another.
**Closeness**	The average distance between one node and all other nodes in the network (by taking the inverse of each edge weight).	Low closeness centrality indicates that node is closely connected with most other nodes, therefore it can spread information most efficiently through a network.
**Strength**	The sum of all absolute edge weights a node is directly connected to.	Likely to be involved with most members of the network.

For peer review only

For peer review only - http://bmjopen.bmj.com/site/about/guidelines.xhtml

eTable 6: Frequency of recorded ICD-10 unipolar depression diagnoses

ICD-10 Code	ICD-10 Diagnosis	n
F32.1	Moderate depressive episode	5,242
F32	Depressive episode	3,998
F41.2	Mixed anxiety and depressive disorder	2,137
F32.0	Mild depressive episode	2,043
F32.9	Depressive episode, unspecified	1,424
F33.1	Recurrent depressive disorder, current episode moderate	1,257
F32.2	Severe depressive episode without psychotic symptoms	1,231
F33	Recurrent depressive disorder	1,123
F33.0	Recurrent depressive disorder, current episode mild	408
F32.8	Other depressive episodes	348
F33.4	Recurrent depressive disorder, currently in remission	326
F33.9	Recurrent depressive disorder, unspecified	276
F33.8	Other recurrent depressive disorders	67

For peer review only

For peer review only - http://bmjopen.bmj.com/site/about/guidelines.xhtml

eTable 7: Multivaribale Cox regression models examining factors associated with subsequent bipolar disorder diagnosis

Predictor	Risk of developing bipolar disorder (number of events = 986)			
	**Univariate Hazard Ratio (95% CI)**	**p value**	**[a]Multivariable Hazard Ratio (95% CI)**	**p value**
**Demographics**				
Age (years)	0.99 (0.98 to 0.99)	<0.001***	0.99 (0.98 to 0.99)	<0.001***
Male gender	0.73 (0.64 to 0.84)	<0.001***	0.77 (0.67 to 0.88)	<0.001***
Ethnicity				
White	*Ref*		*Ref*	*Ref*
Asian	0.86 (0.64 to 1.16)	0.356	0.82 (0.61 to 1.10)	0.229
Black	1.04 (0.88 to 1.22)	0.671	0.96 (0.82 to 1.14)	0.654
Mixed	1.35 (0.93 to 1.95)	0.137	1.21 (0.84 to 1.76)	0.331
Other	0.94 (0.75 to 1.18)	0.621	0.91 (0.72 to 1.14)	0.414
**Symptom groups**				
Mania (≥ 1 symptom)	2.38 (2.10 to 2.71)	<0.001***	2.39 (2.11 to 2.72)	<0.001***
Overlapping (≥ 1 symptom)	1.43 (1.19 to 1.71)	<0.001***	1.45 (1.21 to 1.74)	<0.001***
Depression (≥ 1 symptom)	1.55 (1.09 to 2.19)	0.021*	1.45 (1.02 to 2.05)	0.050*
**Individual symptoms**				
Irritability	1.99 (1.74 to 2.28)	<0.001***	1.99 (1.74 to 2.28)	<0.001***
Elation	4.21 (3.46 to 5.11)	<0.001***	4.21 (3.46 to 5.12)	<0.001***
Pressured speech	3.87 (2.97 to 5.05)	<0.001***	4.07 (3.12 to 5.31)	<0.001***
Flight of ideas	3.78 (2.75 to 5.20)	<0.001***	3.90 (2.84 to 5.36)	<0.001***
Grandiosity	4.14 (3.08 to 5.56)	<0.001***	4.36 (3.24 to 5.87)	<0.001***
Disturbed sleep	1.13 (0.99 to 1.28)	0.089	1.15 (1.00 to 1.31)	0.054
Poor concentration	1.42 (1.25 to 1.61)	<0.001***	1.43 (1.26 to 1.62)	<0.001***
Mood instability	2.08 (1.83 to 2.36)	<0.001***	2.03 (1.79 to 2.30)	<0.001***
Agitation	2.19 (1.93 to 2.49)	<0.001***	2.31 (2.03 to 2.63)	<0.001***
Insomnia	1.52 (1.32 to 1.77)	<0.001***	1.54 (1.33 to 1.78)	<0.001***

[a]Multivariable analysis adjusted for age, gender and ethnicity.
* p < 0.05, ** p < 0.001, *** p < 0.001

For peer review only - http://bmjopen.bmj.com/site/about/guidelines.xhtml
For peer review only

eTable 8: Multivariable Cox regression models examining factors associated with subsequent psychotic disorder diagnosis

Predictor	Risk of developing psychotic disorder (number of events = 2,150)			
	Univariate Hazard Ratio (95% CI)	p value	[a]Multivariable Hazard Ratio (95% CI)	p value
**Demographics**				
Age (years)	1.01 (1.00 to 1.01)	<0.001***	1.01 (1.01 to 1.01)	<0.001***
Male gender	1.10 (1.01 to 1.20)	0.036*	1.12 (1.03 to 1.23)	0.010*
Ethnicity				
White	*Ref*		*Ref*	*Ref*
Asian	1.73 (1.47 to 2.03)	<0.001***	1.80 (1.53 to 2.11)	<0.001***
Black	1.83 (1.65 to 2.02)	<0.001***	1.92 (1.73 to 2.12)	<0.001***
Mixed	0.93 (0.67 to 1.29)	0.671	1.00 (0.72 to 1.39)	0.999
Other	1.03 (0.89 to 1.20)	0.671	1.06 (0.92 to 1.23)	0.448
**Symptom groups**				
Mania (≥ 1 symptom)	1.87 (1.71 to 2.05)	<0.001***	1.89 (1.73 to 2.07)	<0.001***
Overlapping (≥ 1 symptom)	1.85 (1.62 to 2.11)	<0.001***	1.81 (1.59 to 2.07)	<0.001***
Depression (≥ 1 symptom)	1.29 (1.02 to 1.63)	0.038*	1.32 (1.04 to 1.67)	0.023*
**Individual symptoms**				
Irritability	1.80 (1.64 to 1.98)	<0.001***	1.82 (1.65 to 1.99)	<0.001***
Elation	2.04 (1.71 to 2.44)	<0.001***	2.17 (1.81 to 2.60)	<0.001***
Pressured speech	2.67 (2.16 to 3.32)	<0.001***	2.69 (2.17 to 3.33)	<0.001***
Flight of ideas	2.63 (2.08 to 3.32)	<0.001***	2.61 (2.07 to 3.30)	<0.001***
Grandiosity	2.59 (2.02 to 3.33)	<0.001***	2.61 (2.03 to 3.36)	<0.001***
Disturbed sleep	1.71 (1.56 to 1.88)	<0.001***	1.65 (1.50 to 1.81)	<0.001***
Poor concentration	1.56 (1.44 to 1.70)	<0.001***	1.54 (1.42 to 1.68)	<0.001***
Mood instability	1.62 (1.48 to 1.76)	<0.001***	1.70 (1.55 to 1.85)	<0.001***
Agitation	2.42 (2.22 to 2.63)	<0.001***	2.41 (2.21 to 2.63)	<0.001***
Insomnia	1.49 (1.35 to 1.65)	<0.001***	1.50 (1.35 to 1.65)	<0.001***

[a]Multivariable analysis adjusted for age, gender and ethnicity.
* p < 0.05, ** p <0.01, *** p < 0.001

For peer review only

For peer review only - http://bmjopen.bmj.com/site/about/guidelines.xhtml

eTable 9: Multivariable Cox proportional hazards regression examining factors associated with compulsory psychiatric hospital admission

Predictor	Risk of compulsory psychiatric hospital admission under the UK Mental Health Act (number of events = 516)			
	Univariate Hazard Ratio (95% CI)	p value	[a]Multivariable Hazard Ratio (95% CI)	p value
**Demographics**				
Age (years)	1.00 (0.99 to 1.01)	0.91	1.00 (0.99 to 1.01)	0.794
Male gender	1.23 (1.03 to 1.46)	0.029**	1.34 (1.12 to 1.60)	0.002**
Ethnicity				
White	*Ref*	*Ref*	*Ref*	*Ref*
Asian	1.63 (1.16 to 2.29)	0.007**	1.65 (1.17 to 2.32)	0.006**
Black	2.36 (1.95 to 2.86)	<0.001***	2.47 (2.03 to 3.00)	<0.001***
Mixed	1.22 (0.67 to 2.23)	0.560	1.27 (0.70 to 2.33)	0.465
Other	0.72 (0.51 to 1.02)	0.078	0.73 (0.51 to 1.03)	0.084
**Symptom groups**				
Mania (≥ 1 symptom)	2.29 (1.92 to 2.73)	<0.001***	2.28 (1.92 to 2.72)	<0.001***
Overlapping (≥ 1 symptom)	1.68 (1.29 to 2.19)	<0.001***	1.62 (1.25 to 2.11)	0.001**
Depression (≥ 1 symptom)	0.99 (0.63 to 1.54)	0.950	1.00 (0.64 to 1.57)	0.999
**Individual symptoms**				
Irritability	2.29 (1.91 to 2.74)	<0.001***	2.28 (1.90 to 2.73)	<0.001***
Elation	3.64 (2.75 to 4.83)	<0.001***	3.96 (2.98 to 5.25)	<0.001***
Pressured speech	4.41 (3.14 to 6.19)	<0.001***	4.37 (3.12 to 6.14)	<0.001***
Flight of ideas	3.91 (2.69 to 5.69)	<0.001***	3.80 (2.61 to 5.53)	<0.001***
Grandiosity	3.83 (2.54 to 5.77)	<0.001***	3.59 (2.38 to 5.41)	<0.001***
Disturbed sleep	1.55 (1.28 to 1.87)	<0.001***	1.47 (1.21 to 1.78)	<0.001***
Poor concentration	1.48 (1.24 to 1.76)	<0.001***	1.45 (1.22 to 1.73)	<0.001***
Mood instability	1.84 (1.54 to 2.19)	<0.001***	1.92 (1.61 to 2.29)	<0.001***
Agitation	3.75 (3.15 to 4.46)	<0.001***	3.69 (3.10 to 4.39)	<0.001***
Insomnia	1.50 (1.2 to 1.84)	<0.001***	1.51 (1.23 to 1.86)	<0.001***

[a]Multivariable analysis adjusted for age, gender and ethnicity.
* p < 0.05, ** p < 0.01, *** p < 0.001

For peer review only

For peer review only - http://bmjopen.bmj.com/site/about/guidelines.xhtml

eTable 10: DSM-5 mixed features specifier of depression[3]

**Depression with mixed features specifier**
A. At least three of the following manic/hypomanic symptoms are present nearly every day during the majority of days of a major depressive episode:<br>1. Elevated, expansive mood<br>2. Inflated self-esteem or grandiosity<br>3. More talkative than usual or pressure to keep talking<br>4. Flight of ideas or subjective experience that thoughts are racing<br>5. Increase in energy or goal-directed activity (either socially, at work or school, or sexually)<br>6. Increased or excessive involvement in activities that have a high potential for painful consequences<br>7. Decreased need for sleep (feeling rested despite sleeping less than usual; to be contrasted with insomnia)<br><br>B. Mixed symptoms are observable by others and represent a change from the person's usual behaviour.<br>C. For individuals whose symptoms meet full criteria for either mania or hypomania, the diagnosis should be bipolar I or bipolar II disorder.<br>D. The mixed symptoms are not attributable to the physiological effects of a substance (e.g., a drug of abuse, a medication or other treatment).<br><br>**Note:** Mixed features associated with a major depressive episode have been found to be a significant risk factor for the development of bipolar I or bipolar II disorder. As a result, it is clinically useful to note the presence of this specifier for treatment planning and monitoring of response to treatment.

For peer review only - http://bmjopen.bmj.com/site/about/guidelines.xhtml

eFigure 1: Cohort definition and missing data

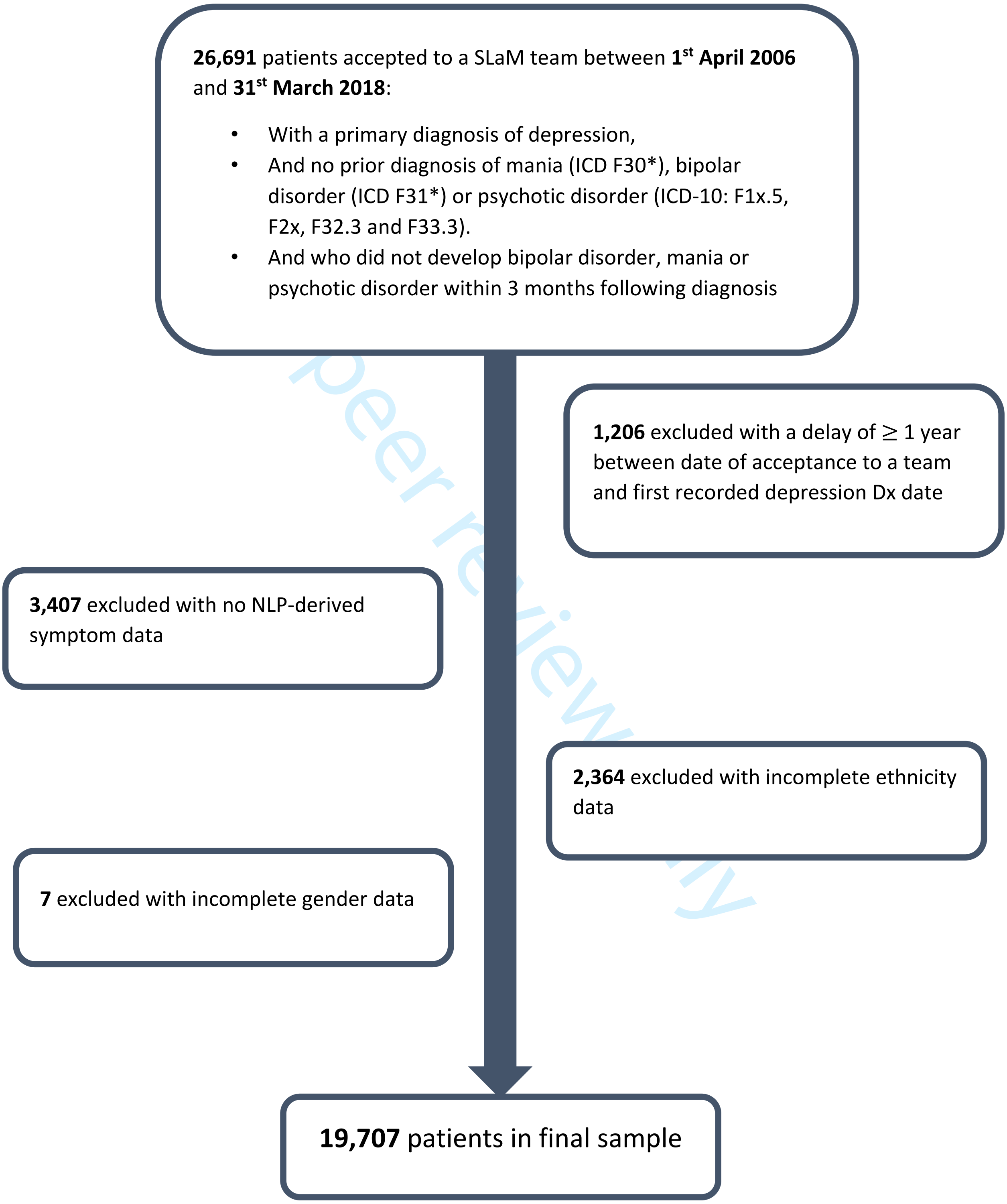

For peer review only

For peer review only - http://bmjopen.bmj.com/site/about/guidelines.xhtml

eFigure 2: Centrality indices for overall network

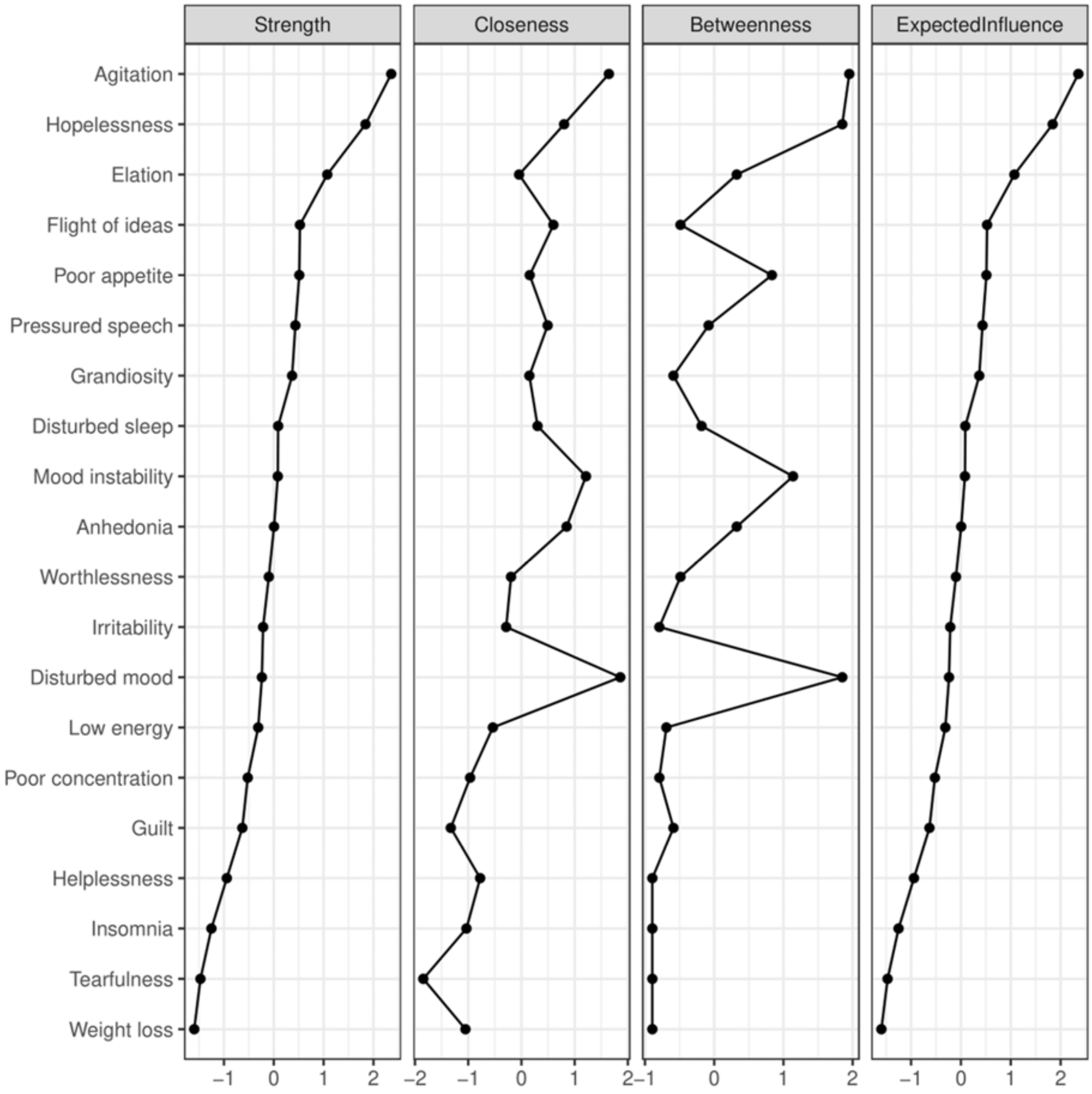

Note: Centrality indices are reported as standardised z scores to facilitate comparison.

For peer review only - http://bmjopen.bmj.com/site/about/guidelines.xhtml

References

1 Epskamp S, Borsboom D, Fried EI. Estimating psychological networks and their accuracy: A tutorial paper. *Behav Res Methods* 2018;**50**:195–212. doi:10.3758/s13428-017-0862-1

2 Golbeck J. *Analyzing the Social Web*. Elsevier Inc. 2013. doi:10.1016/C2012-0-00171-8

3 American Psychological Association. *Diagnostic and statistical manual of mental disorders : DSM-5*. Fifth edition. Arlington, VA : American Psychiatric Publishing, [2013] ©2013 2013. https://search.library.wisc.edu/catalog/9911111397702121

For peer review only

For peer review only - http://bmjopen.bmj.com/site/about/guidelines.xhtml

The RECORD statement – checklist of items, extended from the STROBE statement, that should be reported in observational studies using routinely collected health data.

	Item No.	STROBE items	Location in manuscript where items are reported	RECORD items	Location in manuscript where items are reported
**Title and abstract**					
	1	(a) Indicate the study's design with a commonly used term in the title or the abstract (b) Provide in the abstract an informative and balanced summary of what was done and what was found	Pages 1 and 3	RECORD 1.1: The type of data used should be specified in the title or abstract. When possible, the name of the databases used should be included.<br><br>RECORD 1.2: If applicable, the geographic region and timeframe within which the study took place should be reported in the title or abstract.<br><br>RECORD 1.3: If linkage between databases was conducted for the study, this should be clearly stated in the title or abstract.	Page 3<br><br>Page 3<br><br>N/A
**Introduction**					
Background rationale	2	Explain the scientific background and rationale for the investigation being reported	Page 5		
Objectives	3	State specific objectives, including any prespecified hypotheses	Page 5		
**Methods**					
Study Design	4	Present key elements of study design early in the paper	Page 6		
Setting	5	Describe the setting, locations, and relevant dates, including periods of recruitment, exposure, follow-up, and data collection	Page 6		

For peer review only

Participants	6	*(a) Cohort study* - Give the eligibility criteria, and the sources and methods of selection of participants. Describe methods of follow-up<br>*Case-control study* - Give the eligibility criteria, and the sources and methods of case ascertainment and control selection. Give the rationale for the choice of cases and controls<br>*Cross-sectional study* - Give the eligibility criteria, and the sources and methods of selection of participants<br><br>*(b) Cohort study* - For matched studies, give matching criteria and number of exposed and unexposed<br>*Case-control study* - For matched studies, give matching criteria and the number of controls per case	Page 6<br><br><br><br><br>N/A – No matching	RECORD 6.1: The methods of study population selection (such as codes or algorithms used to identify subjects) should be listed in detail. If this is not possible, an explanation should be provided.<br><br>RECORD 6.2: Any validation studies of the codes or algorithms used to select the population should be referenced. If validation was conducted for this study and not published elsewhere, detailed methods and results should be provided.<br><br>RECORD 6.3: If the study involved linkage of databases, consider use of a flow diagram or other graphical display to demonstrate the data linkage process, including the number of individuals with linked data at each stage.	Page 6<br><br><br>Page 7<br><br><br>N/A – No linkage
Variables	7	Clearly define all outcomes, exposures, predictors, potential confounders, and effect modifiers. Give diagnostic criteria, if applicable.	Pages 6 to 7	RECORD 7.1: A complete list of codes and algorithms used to classify exposures, outcomes, confounders, and effect modifiers should be provided. If these cannot be reported, an explanation should be provided.	Pages 6 to 7
Data sources/ measurement	8	For each variable of interest, give sources of data and details of methods of assessment (measurement).<br>Describe comparability of assessment methods if there is more than one group	Pages 6 to 7		

For peer review only

Bias	9	Describe any efforts to address potential sources of bias	Pages 6 to 7		
Study size	10	Explain how the study size was arrived at	Page 9		
Quantitative variables	11	Explain how quantitative variables were handled in the analyses. If applicable, describe which groupings were chosen, and why	Page 8		
Statistical methods	12	(a) Describe all statistical methods, including those used to control for confounding<br>(b) Describe any methods used to examine subgroups and interactions<br>(c) Explain how missing data were addressed<br>(d) *Cohort study* - If applicable, explain how loss to follow-up was addressed<br>*Case-control study* - If applicable, explain how matching of cases and controls was addressed<br>*Cross-sectional study* - If applicable, describe analytical methods taking account of sampling strategy<br>(e) Describe any sensitivity analyses	Pages 7 to 8<br>Page 6<br>Page 6<br>N/A<br>N/A<br>N/A<br>N/A		
Data access and cleaning methods		..		RECORD 12.1: Authors should describe the extent to which the investigators had access to the database population used to create the study population.	Page 6

For peer review only

				RECORD 12.2: Authors should provide information on the data cleaning methods used in the study.	Page 6
Linkage		..		RECORD 12.3: State whether the study included person-level, institutional-level, or other data linkage across two or more databases. The methods of linkage and methods of linkage quality evaluation should be provided.	N/A – No linkage
**Results**					
Participants	13	(a) Report the numbers of individuals at each stage of the study (*e.g.*, numbers potentially eligible, examined for eligibility, confirmed eligible, included in the study, completing follow-up, and analysed)<br>(b) Give reasons for non-participation at each stage.<br>(c) Consider use of a flow diagram	Page 9<br><br>Page 9<br><br>eFigure 1	RECORD 13.1: Describe in detail the selection of the persons included in the study (*i.e.,* study population selection) including filtering based on data quality, data availability and linkage. The selection of included persons can be described in the text and/or by means of the study flow diagram.	Page 9 and eFigure 1
Descriptive data	14	(a) Give characteristics of study participants (*e.g.*, demographic, clinical, social) and information on exposures and potential confounders<br>(b) Indicate the number of participants with missing data for each variable of interest<br>(c) *Cohort study* - summarise follow-up time (*e.g.*, average and total amount)	Page 9 and Table 1<br><br>Page 9 and eFigure 1<br><br>Page 9		
Outcome data	15	*Cohort study* - Report numbers of outcome events or summary measures over time<br>*Case-control study* - Report numbers in each exposure	Page 10<br><br>N/A		

For peer review only

		category, or summary measures of exposure<br>*Cross-sectional study* - Report numbers of outcome events or summary measures	N/A		
Main results	16	(a) Give unadjusted estimates and, if applicable, confounder-adjusted estimates and their precision (e.g., 95% confidence interval). Make clear which confounders were adjusted for and why they were included<br>(b) Report category boundaries when continuous variables were categorized<br>(c) If relevant, consider translating estimates of relative risk into absolute risk for a meaningful time period	Page 9 and 10<br>Tables 3 and 4<br>Tables 3 and 4		
Other analyses	17	Report other analyses done—e.g., analyses of subgroups and interactions, and sensitivity analyses	Page 10 and Supplementary Material		
**Discussion**					
Key results	18	Summarise key results with reference to study objectives	Page 11		
Limitations	19	Discuss limitations of the study, taking into account sources of potential bias or imprecision. Discuss both direction and magnitude of any potential bias	Page 13	RECORD 19.1: Discuss the implications of using data that were not created or collected to answer the specific research question(s). Include discussion of misclassification bias, unmeasured confounding, missing data, and changing eligibility over time, as they pertain to the study being reported.	Page 13
Interpretation	20	Give a cautious overall interpretation of results considering objectives,	Pages 11 to 13		

For peer review only

		limitations, multiplicity of analyses, results from similar studies, and other relevant evidence			
Generalisability	21	Discuss the generalisability (external validity) of the study results	Pages 12 to 13		
**Other Information**					
Funding	22	Give the source of funding and the role of the funders for the present study and, if applicable, for the original study on which the present article is based	Page 2		
Accessibility of protocol, raw data, and programming code		..		RECORD 22.1: Authors should provide information on how to access any supplemental information such as the study protocol, raw data, or programming code.	Page 2

*Reference: Benchimol EI, Smeeth L, Guttmann A, Harron K, Moher D, Petersen I, Sørensen HT, von Elm E, Langan SM, the RECORD Working Committee. The REporting of studies Conducted using Observational Routinely-collected health Data (RECORD) Statement. *PLoS Medicine* 2015; in press.

*Checklist is protected under Creative Commons Attribution (CC BY) license.

For peer review only
